# Embryonic and postnatal macrophages are necessary for proper tooth development and homeostasis

**DOI:** 10.1038/s41467-026-75576-7

**Published:** 2026-07-17

**Authors:** Marcos Gonzalez Lopez, Kaitlin A. Katsura, Vitor C. M. Neves, Ruslan Soldatov, Josef Lavicky, Maryam Azam, Michaela Kavkova, Klara Cigosova, Brian Temsamrit, Johnny Bonnardel, Hannah Gong, Kelsey M. Nemec, Frederick Christian Bennett, Haneen Tuaima, Val Yianni, Paul T. Sharpe, Marc Bajenoff, Peter Kharchenko, Mariko L. Bennett, Igor Adameyko, Jan Krivanek

**Affiliations:** 1 Department of Histology and Embryology, Faculty of Medicine, Masaryk University, Brno, Czech Republic; 2 Division of Human Genetics, Children’s Hospital of Philadelphia, Philadelphia, PA USA; 3 Institute for Translational Medicine and Therapeutics, University of Pennsylvania, Philadelphia, PA USA; 4 Department of Orofacial Sciences, University of California, San Francisco, CA USA; 5 Restorative Dentistry Unit, The School of Clinical Dentistry, University of Sheffield, Sheffield, UK; 6 Department of Epidemiology and Biostatistics, Computational Oncology, Memorial Sloan Kettering Cancer Center, New York, NY USA; 7 Division of Neurology, Children’s Hospital of Philadelphia, Philadelphia, PA USA; 8 Department of Psychiatry, University of Pennsylvania, Philadelphia, PA USA; 9 Centre d’Immunologie de Marseille-Luminy, Aix Marseille Université, INSERM, CNRS, Marseille, France; 10 Department of Neuroscience, University of Pennsylvania, Philadelphia, PA USA; 11 Centre for Craniofacial and Regenerative Biology, FoDOCS, King’s College London, London, UK; 12 Department of Biomedical Informatics, Harvard Medical School, Boston, MA USA; 13 San Diego Institute of Science, Altos Labs, San Diego, CA USA; 14 Department of Neurology, Perelman School of Medicine, Philadelphia, PA USA; 15 Department of Physiology and Pharmacology, Karolinska Institutet, Stockholm, Sweden; 16 Department of Neuroimmunology, Center for Brain Research, Medical University Vienna, Vienna, Austria

**Keywords:** Morphogenesis, Stem cells, Self-renewal

## Abstract

The role of macrophages in teeth, beyond their function in innate immunity, remains unexplored. This study demonstrates that macrophages populate dental tissues during early development and increase in number during pre-eruptive stages postnatally. In continuously growing teeth, they are associated with epithelial and mesenchymal stem cell niches throughout lifetime. To investigate their role in development, we genetically disrupt macrophage migration using neural-crest specific *Wnt1*^*Cre*^*/Csf1*^*fl/fl*^ and general *Csf1R knockout* mouse models. This results in abnormal dentin and enamel deposition, eruption defects, early tooth mispatterning, and impaired osteogenesis. Notably, the phenotype is partially rescued via bone marrow transplantation. In addition, the short-term pharmacological depletion of macrophages in wildtype adult mice causes striking disintegration of both dentin and enamel in the apical part of continuously-growing teeth, which is restored after withdrawal of clodronate. Following treatment, macrophages rapidly repopulate dental tissue and the presence of M2 reparative state phenotype is observed by single-cell RNA-seq analysis. Overall, our findings reveal essential role of macrophages in the dental development and patterning of both mesenchymal and epithelial tooth compartments. This previously unrecognized role of macrophages in teeth is reminiscent of their function in complex tissue regeneration and requires future studies to dissect precise cellular and molecular mechanisms.

## Abstract (web summary)

Our study establishes macrophages as necessary components of the developing tooth microenvironment to maintain their morphology and function. By integrating complementary experimental approaches, we show that macrophage depletion disrupts multiple aspects of tooth morphogenesis and homeostasis, providing new insight into the cellular interactions that coordinate dental tissue formation and maintenance.

## Introduction

Macrophages regulate complex cellular dynamics of tissues during development, growth, repair, and homeostasis in all tissues, as reviewed by many research teams^1–5^. Beyond their critical immune role as first-line defenders against pathogens, they initiate adaptive immune responses, clear apoptotic and senescent cells, and actively shape tissue architecture during organogenesis and regeneration^6–9^. Macrophages can be broadly classified according to their developmental origin: (i) monocyte-derived, which differentiate from bone marrow-derived monocytes that circulate in the bloodstream and enter tissue in response to tissue homeostasis or inflammation, and (ii) tissue-resident macrophages, which originate from embryonic erythro-myeloid progenitors in the yolk sac or fetal liver, which seed developing tissues during embryogenesis and can persist into adulthood^10^. Tissue-resident macrophages acquire tissue-specific functions and are present in numerous tissues, including, e.g., Kupffer cells in liver, microglia in brain, osteoclasts in bone, alveolar macrophages in lungs, Hofbauer cells in placenta, Langerhans cells in skin and many others^1,7^. Importantly, there is increasing evidence that macrophages are also crucial for the maintenance of stem cell niches in various tissues, including intestines, muscles, mammary gland, the hematopoietic niche and others^11–15^. In this study, we focus on “tooth macrophages” as a broad term for the macrophage population located in the mice's teeth regardless of their primary origin (monocyte-derived or tissue-resident). Despite the advances in studying macrophages in different organs, the role of tooth macrophages in the context of the stem cell niche of incisor and molar morphogenesis still remains a completely unexplored area.

Teeth are highly mineralized structures located at the beginning of most vertebrate digestive tracts and they vary in their form, replacement, and growth across species. Despite these differences, vertebrate teeth share (with some degree of variation) essentially two parts: (1) the crown composed primarily of two mineralized tissues, dentin and enamel, which are respectively formed by odontoblasts and ameloblasts; and (2) the root, which is comprised of dentin and cementum. These hard tissues surround and protect the highly vascularized and innervated dental pulp where dentin-forming odontoblasts are located^16,17^. While the ameloblasts are lost during the eruption of the crown, the dental pulp allows teeth to function as a living organ capable of responding to stimuli from the external environment. Teeth cannot be considered as isolated organs but should be understood as an inseparable part of a complex system in which the surrounding alveolar bone, along with other periodontal structures (gingiva, periodontal ligaments and cementum), participate in tooth development and function.

Mice's teeth have proven to be an excellent model for studying tooth development, particularly the incisor, due to its ever-growing capacity. This growth pattern is unusual because it relies on a permanently active stem cell niche, allowing its continuous regeneration, unlike the molars of mice or all human teeth^18–22^. In addition, studies using the rodent dentition have demonstrated the process of alveolar bone resorption, making it a traditional model for understanding tooth eruption^23,24^. Tooth eruption is a temporally and spatially precisely regulated process that requires the active participation of a dental follicle, leading to properly controlled alveolar bone resorption and subsequent alveolar bone formation at the base of the bony crypt^25^.

Recent studies focused on independent classification of various dental cell types via methods of single-cell RNA-sequencing (scRNA-seq) have revealed a proportionally substantial amount of immune cells present in both human and mouse teeth with diverse expression profiles^26,27^. These include neutrophils, B- and T-cells, or macrophages in both dental pulp and periodontal space^22,24–28^. Although previously, the role of immune cells has primarily been studied in the context of inflammatory conditions such as pulpitis or periodontitis ^29–32^, the above-mentioned scRNA-seq studies have worked with healthy dental tissues^33,34^. Besides immune functions, emerging studies have shown macrophages to be key players in reparative dentinogenesis^35,36^ in both human and mouse teeth. In addition to macrophages, dendritic cells have also been shown to participate in immunosurveillance and antigen presentation during pulp inflammation^34,37,38^. Historically, tissue macrophages have been categorized according to their activity into M0-like (resting state), M1-like (pro-inflammatory and M2-like (anti-inflammatory/healing) subtypes^39–41^. In human teeth, M2-like macrophages have been reported to be present in association with glial cells in both healthy and healing teeth^42^. Similarly, macrophages in mice teeth are involved in the activation of dental pulp stem cells upon tooth damage and participate in tooth healing during tertiary dentinogenesis, which encompasses both reactionary and reparative dentinogenesis^35^. Such functions in tooth repair, combined with the high occurrence of macrophages identified in unerupted continuously growing incisors^26^, highlight a key functional role in homeostasis and maintenance, beyond those of the innate immune system and healing.

This study investigates the role of tooth macrophages independently of their origin (this term does not distinguish between monocyte-derived or tissue resident) during tooth development, growth and interaction with the stem cell niche during continuous tooth growth. We employed a comprehensive methodological approach to uncover hitherto unknown roles of macrophages in dental tissues, and the consequences of their induced dysfunction. This included both genetic and pharmacological ablation of macrophages at different developmental stages, followed by phenotype rescue by bone marrow transplantation. The genetic ablation strategy was based on perturbation of *Csf1/Csf1r* signaling, which is essential for macrophage differentiation, proliferation and survival. This was achieved through two complementary approaches: (1) depleting *Csf1* ligand in neural crest derivatives using the Wnt1-Cre driver (*Wnt1*^*Cre*^*/Csf1*^*fl/fl*^), and (2) global knockout of *Csf1r (Csf1r KO)*. In addition, we temporarily induced a reduction of the macrophage population by clodronates.

Our results show that macrophages first appear during early stages of tooth development—in the condensed dental mesenchyme. Postnatally, prior to eruption, macrophage numbers expand sharply within the dental pulp while the teeth are still developing. Accordingly, we investigated the temporal dynamics of macrophage and osteoclast localization in key regions essential for tooth eruption and root formation, namely the superior wall of the alveolar crypt and the dental follicle. Using *Wnt1*^*Cre*^*/Csf1*^*fl/fl*^ and *Csf1r KO* mouse strains, we demonstrate that depletion of macrophages results in pronounced abnormalities in molar development and incisor growth. Our results show that depletion of macrophages leads to multiple developmental abnormalities, including disrupted tooth morphology, malformations in hard dental tissues, delay of tooth growth, or improper early tooth patterning, which in one case was manifested by the presence of a supernumerary tooth. Additionally, we show that the induced phenotype can be partially rescued by transplantation of bone marrow from healthy mice, which express a fluorescent tag to track the engraftment. To demonstrate the role of macrophages during the maintenance of stem cell niche homeostasis and to test the reversibility of this process, we performed pharmacologic ablation of macrophages in young and adult mice. This procedure led to temporary and reversible disruption of incisor growth affecting enamel and dentin patterning. Subsequent scRNA-seq analysis revealed a rapid acquisition of M2-like macrophage phenotype in pharmacologically treated animals compared to untreated controls.

Taken together, our study reveals a previously unrecognized role of macrophages during tooth formation and homeostasis. Our findings demonstrate that macrophages are essential for proper tooth development and, in parallel, are closely associated and interact with the epithelial stem cell niche in continuously growing teeth. Our results using a tooth as a model system may provide insights into the cellular mechanisms governing the development, growth and regeneration of other tissues.

## Results

### Tooth macrophages populate early embryonic developing teeth and expand postnatally before eruption

**Fig. 1:**
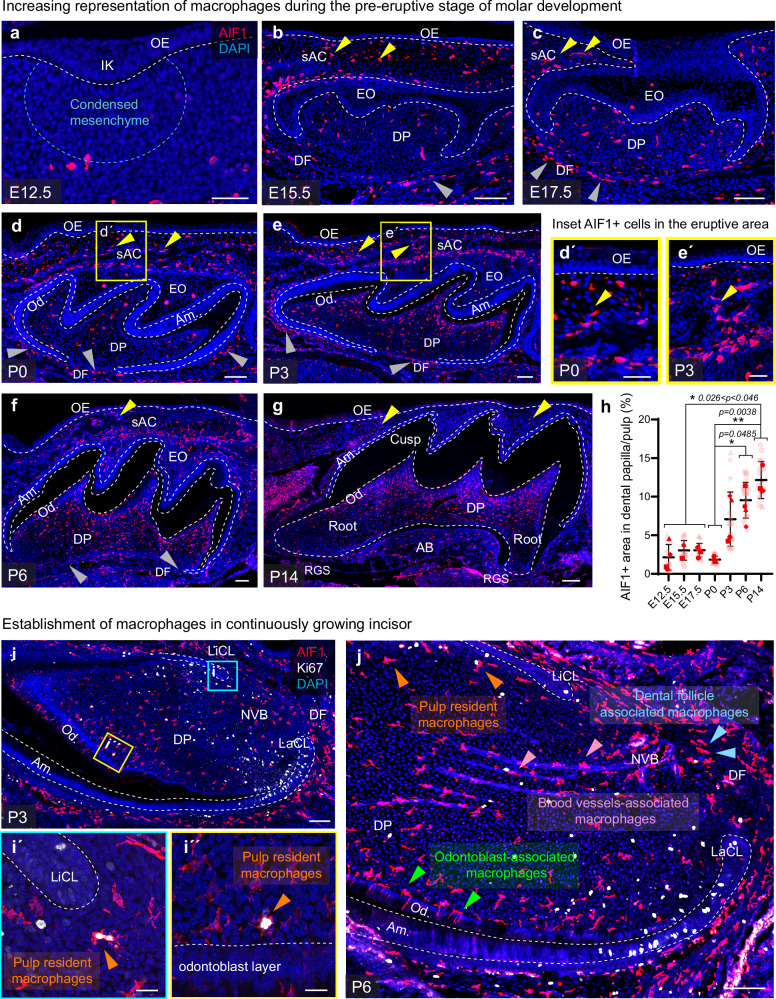
Dynamics and distribution of macrophages during tooth development. Macrophages are present during the development of molars from their early prenatal stages (**a**–**c**). Subsequent early postnatal pre-eruptive developmental stages (P0, P3, P6, and P14) show a progressive increase of macrophages colonizing the dental pulp, the eruption space at the superior wall of the alveolar crypt (yellow arrowheads) and around the dental follicle (grey arrowheads) (**d**–**g**). Quantification of AIF1-positive cells across selected developmental timepoints demonstrates a significant rise in macrophage numbers in dental papilla/pulp, particularly during postnatal stages (**h**). Each symbol represents one biologically independent animal (*n* = 4 animals per developmental timepoint). Data are presented as mean ± SD. Statistical significance was assessed using a two-sided one-way ANOVA followed by Tukey’s multiple-comparisons test. Exact *p* values are shown in the graph; ^*^p < 0.05, ^**^p < 0.01, ^***^p < 0.001, ^****^p < 0.0001. Similarly, in early postnatal (P3) pre-eruptive stage of continuously-growing incisor, macrophages are widely present in the pulp, where they are ubiquitously distributed including region around epithelial cervical loops and inside odontoblast layer (**i**). Macrophages also proliferate directly inside the developing pulp as shown by AIF1/mKI67 (orange arrows) co-staining (**i´**, **i´´**). **j** Heterogeneous distribution of macrophages in early postnatal (P6) continuously growing incisor which are associated with blood vessels (pink arrowheads), odontoblasts (green arowheads), dental follicle (cyan arrowheads) or pulp-resident (orange arrowheads). All scale bars = 100 μm; insets (**i´**, **i´´**) = 50 μm. AB alveolar bone, Am. ameloblasts, DF dental follicle, DP dental papilla/pulp, E embryonic day, EO enamel organ, IK initiation knot, LaCL labial cervical loop, LiCL lingual cervical loop, NVB neurovascular bundle, Od. odontoblasts, OE oral epithelium, P postnatal day, RGS root growing space, sAC superior wall of alveolar crypt.

Macrophages constitute a significant portion of the cellular composition of dental tissue^26,27^. Although it is well known that they act as the first line of immunological defense, it is unclear how macrophages act during pre-eruptive stages of tooth development. To explore how they populate developing teeth, we investigated the distribution and the dynamics of establishment of their population during the mouse molar development (Fig. 1a–h). To investigate this aspect, we detected AIF1 (allograft inflammatory factor 1, also known as IBA1), which is highly and specifically expressed in the monocytic lineage, including macrophages and microglia^43^. AIF1 is a calcium-binding protein that participates in several intracellular processes (phagocytosis, membrane ruffling and F-actin remodeling) as well as activation of macrophages upon inflammation^44^. Starting from E12.5 (Embryonic day 12.5, *post coitum*), we detected AIF1+ macrophages in the periphery of the condensed mesenchyme (Fig. 1a). Later, by E15.5 and E17.5, their expansion gradually increased throughout the dental papilla/pulp. Macrophages appeared homogenously spread in the dental papilla/pulp both before and after birth. This included the apical and central part of the pulp, pulp horns and the odontoblast layer (Fig. 1a–c). The abundance of AIF1+ cells in the dental papilla/pulp dramatically increased shortly after birth (Fig. 1d–g). At embryonic timepoints E12.5-E17.5 and P0 (postnatal day 0), the mean of macrophages-positive area ranged from 0.75 to 2%, while during later, still pre-eruptive P3-P14 stages it ranged from 9.5 to 13% (Fig. 1h). Besides the dental papilla/pulp mesenchyme, the presence of AIF1+ cells was also observed in the close surroundings of the tooth, including the superior wall of the alveolar crypt (the space between the enamel organ and the oral epithelium) and the dental follicle at different time points (Fig. 1b–g, and Supplementary Fig. 1a–c). The presence of AIF1+ cells was also detected during later root elongation process at P14 in the dental follicle root-growing space (Fig. 1g). AIF1+ cells as monocyte-lineage derivatives, share a common developmental history with osteoclasts. These cells, characterized by expression of Cathepsin K (CTSK)^45,46^, are essential for bone resorption, allowing root elongation and tooth eruption (Supplementary Fig. 1d–f).

Similar to the molars, macrophages populated the dental pulp of the continuously growing mouse incisor (Fig. 1i, j). Co-staining of macrophages with mKI67, a marker of proliferating cells, revealed their proliferative capacity within the teeth, suggesting that these cells are maintained through local proliferation. (Fig. 1i). The spatial distribution of macrophages in this tooth was not stochastic, but showed an association with other cell types and histological structures such as the neurovascular bundle, the odontoblast (and sub-odontoblast) layer, dental pulp cells or dental follicle region (Fig. 1j).

### Cellular and molecular interactions of macrophages with other cell types and stem cell niche

**Fig. 2:**
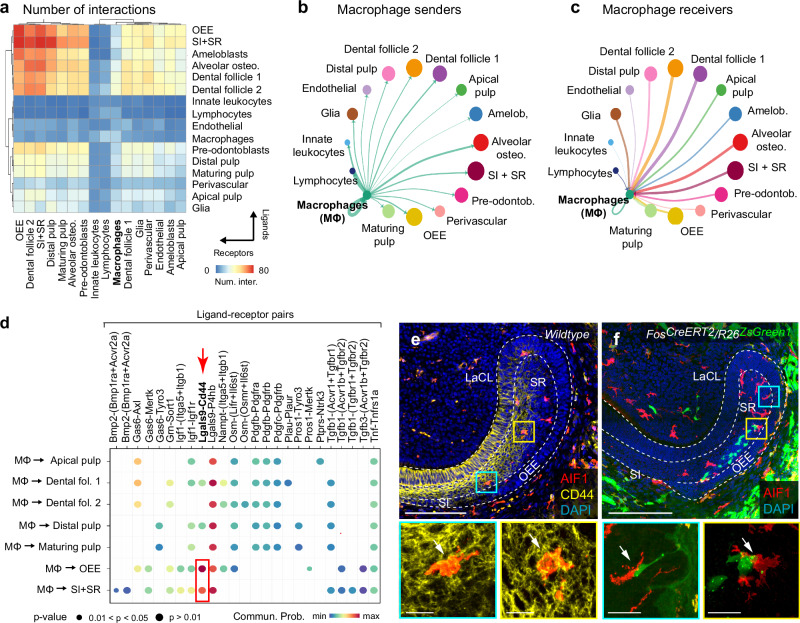
Interaction potential of macrophages with other cell types in tooth. Quantitative heatmap showing the number of interaction pairs among different cell clusters of continuously growing tooth (**a**). Circle plots show the numbers of potential interactions of macrophages and other cell types as senders (**b**) and receivers (**c**). Bubble plot suggests the communication probability of each ligand-receptor interaction pair between macrophages and dental pulp, dental follicle and dental epithelium (**d**). Bubble color indicates communication probability, while bubble size reflects statistical significance as indicated in the graph (small bubbles, 0.01 < *P* < 0.05; large bubbles, *P* < 0.01). Potential intercellular interactions were inferred using the CellChat workflow based on cell-type annotations and gene expression profiles from previously published single-cell RNA-sequencing data^26^. Overexpressed ligand–receptor pairs were prioritized using an absolute fold-change cutoff of 0.1 and *P* < 0.01. This analysis suggested that macrophages potentially communicate with the dental epithelium of labial cervical loop via CD44-Lgals9 axis. Based on this, the expression of CD44 was proved using immunohistochemistry in the stellate reticulum and stratum intermedium of labial cervical loop (white arrows pointing at macrophages) (**e**). Macrophages (white arrows) on the edge of the stellate reticulum and outer enamel epithelium are in direct physical contact with transient epithelial progenitors which were lineage traced using the *Fos*^*CreERT2*^*/R26*^*ZsGreen1*^ mouse line (**f**). Scale bars = 100 µm, insets = 20 µm. LaCL labial cervical loop, OEE outer enamel epithelium, SI stratum intermedium, SR stellate reticulum.

In the tooth, similar to other organs such as the mammary gland or salivary gland, macrophages are in close contact with heterogenous cell populations as an integral part of the microenvironment^13,47^. Based on previous single-cell transcriptomics data^26^, we delved into the interaction and cell communication between macrophages and the rest of the cell clusters present in the mouse incisor. Although, macrophage cluster is not among the most interactive cell groups, a range of 25–50 interaction partners mainly with dental follicle, stellate reticulum (SR), stratum intermedium (SI) and outer enamel epithelium (OEE) was detected (Fig. 2a). The subsequent analysis of separate signals revealed the interaction probability between macrophages as senders (expression of ligand) and receivers (expression of receptor) and other cell types (Fig. 2b, c). To understand these interaction pairs not only on a statistical level, but on the level of concrete ligand-receptor pairs, we performed the CellChat analysis^48^. This tool quantitatively infers intercellular communication networks and detected particular ligand-receptor pairs, including the communication probability (Fig. 2d). To better understand the role of macrophages in the dental epithelial stem cell niche, we further focused on the macrophage-dental epithelium cluster interactions. Here we found a specific *Lgals9-Cd44* pair with high communication probability. The expression of CD44 was validated on the protein level in the progenitor region of dental epithelium^49^ and it co-localized with position of AIF1+ macrophages (Fig. 2e). Additionally, in the stellate reticulum of labial cervical loop (LaCL), we identified macrophages closely associated with previously discovered *Fos*^*CRERT2*^*/R26*^*ZsGreen1*^-lineage traced transient progenitors of OEE (Fig. 2f). Taken together, these findings suggest that macrophages might play a role in epithelial stem cell niche homeostasis.

### Neural crest derivatives-specific *Csf1*-genetic disruption leads to severe congenital dental defects

The *Csf1/Csf1r* system is central to macrophage survival, proliferation, differentiation, and migration (chemotaxis) in various organs^50,51^. By investigating potential homing factors of macrophages using previously published scRNA-seq data, we noted that the cell cluster previously identified as macrophages expresses colony-stimulating factor 1 receptor (*Csf1r*)^26,52,53^ (Supplementary Fig. 2a-c). To confirm the identity of *Csf1r+* cell cluster as macrophages, we performed a differential gene expression (DGE) analysis, which proved the co-expression of other well-established molecular identifiers of macrophages (e.g., *F4/80*, *Aif1*, *Mrc1*, *Cxcl16* and others) (Supplementary Fig. 2d)^54–56^. In parallel, the expression of the corresponding *Csf1* ligand in dental pulp cells suggested that the presence of macrophages in this region can be controlled by CSF1-CSF1R interaction axis (Supplementary Fig. 2a–c). The expression of the *Csf1* in the dental tissue was not restricted to the adult stages only, but both *Csf1* and *Csf1r* were present in the developing molars and incisors within the dental papilla/pulp, dental follicle, odontoblast layer and inside the dental epithelium (Supplementary Fig. 3).

**Fig. 3:**
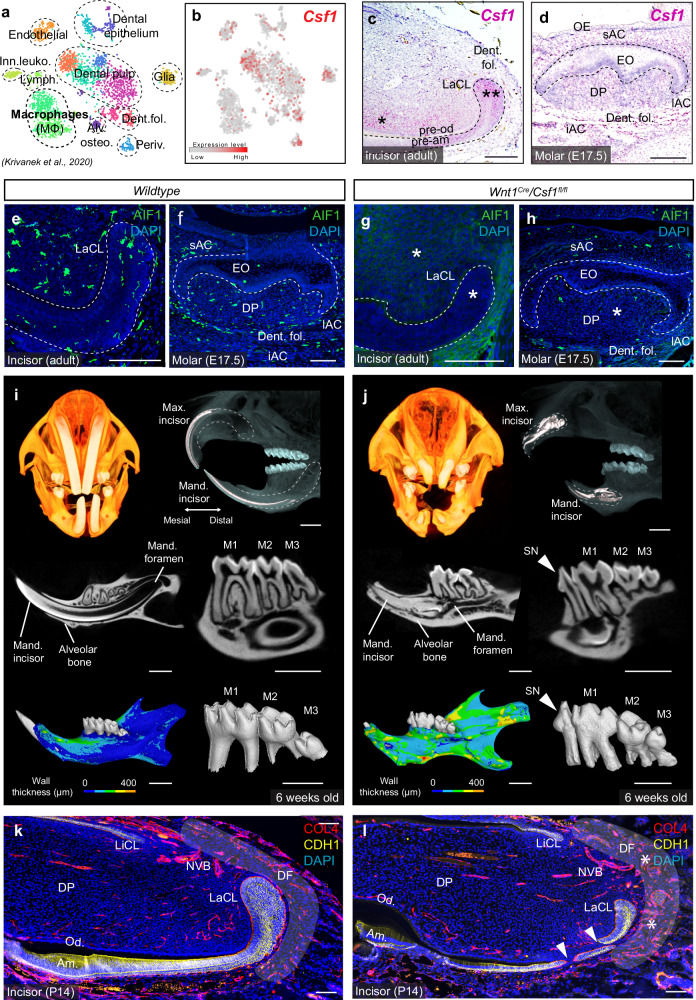
Wnt1^Cre^/Csf1^fl/fl^ induced depletion of macrophages and associated defects on adult dentition. *Csf1* (ligand) is present during both incisor growth and molar development, as predicted by scRNA-seq analysis obtained from adult mouse incisor dataset (**a**–**d**). The highest expression of *Csf1* ligand is in the adult incisor aggregated in the dental pulp within the sub-odontoblastic area (asterisk) and in epithelium (double asterisk), mostly in most apical part of labial cervical loop (**c**). Genetic ablation of macrophages using *Wnt1*^*Cre*^*/Csf1*^*fl/fl*^ mouse strain cause almost complete depletion of macrophages in both mesenchymal and epithelial compartments of an apical part of continuously-growing tooth (asterisk) as well as in the developing mouse molars (**e**–**h**). MicroCT of *Wnt1*^*Cre*^*/Csf1*^*fl/fl*^ mice revealed large dental defects including shortened incisors, reduction pulp cavity, dentin and enamel malformations or the presence of a supernumerary tooth along with mandibular alveolar thickening (wall thickness analysis) while preserving its architecture compared to control mice (**i**, **j**). *Wnt1*^*Cre*^*/Csf1*^*fl/fl*^ mice display an altered cervical loop shape, disruption of the epithelial layer (white arrowheads), and increased vascularization (asterisks) in the dental follicle space (**k**, **l**). Scale bars (**c**–**h**, **k**, **l**) = 100 µm; (**i**, **j**) = 1 mm. Alv. Osteo. alveolar osteocytes, Am. ameloblasts, Dent. fol./DF dental follicle, DP dental pulp, EO enamel organ, iAC inferior wall of alveolar crypt, Inn. Leuko. innate leukocytes, lAC lateral wall of alveolar crypt, LaCL labial cervical loop, LiCL lingual cervical loop, Lymph. lymphocytes, Mand. mandibular, Max. maxillary, M1/2/3 molar 1/2/3, NVB neurovascular bundle, Od. odontoblasts, OE oral epithelium, Periv. perivascular, pre-am pre-ameloblasts, pre-od pre-odontoblasts, sAC superior wall of alveolar crypt, SN supernumerary tooth.

To investigate the role of macrophages in dental tissue, we employed the following strategies. Firstly, we crossed the *Wnt1*^*Cre*^ mouse strain, which specifically expresses a *Cre-*recombinase in neural crest-derived cells, to a *Csf1*^*fl/fl*^ strain. Our results suggest that macrophages first colonize the neural-crest-derived ectomesenchyme and later migrate into the epithelial compartment. Therefore, genetically-modified mouse allowed us to investigate the role of macrophages in teeth after targeted ablation of *Csf1* in dental mesenchyme and other associated neural crest derivatives (Fig. 3). We performed in situ hybridization (ISH) to validate the expression of *Csf1* predicted by scRNA-seq (Fig. 3a–d). This revealed *Csf1* expression in the apical region (progenitor area) of adult incisors (Fig. 3c) and developing molars (Fig. 3d). In mutant *Wnt1*^*Cre*^*/Csf1*^*fl/fl*^ mice, AIF1+ macrophages are almost absent in incisors and molars compared to wild-type animals (Fig. 3e–h). Interestingly, although the conditional deletion of *Csf1* by *Wnt1*^*Cre*^*/Csf1*^*fl/fl*^ was targeted to the mesenchymal part of the tooth, macrophages were not observed either in the epithelial compartment of growing teeth (Fig. 3g). Although AIF1+ cells in the dental pulp and LaCL may morphologically resemble dendritic cells, we further investigated this by examining the expression of Dcstamp (characteristic molecular marker of cells derived from the common myeloid precursor, such as dendritic cells and osteoclasts) using a single-cell mouse incisor dataset^26^ and we did not observe co-expression of *Dcstamp* in *Aif1*+ cluster (Supplementary Fig. 2e).

MicroCT analysis revealed that the incisors of *Wnt1*^*Cre*^*/Csf1*^*fl/fl*^ animals exhibited an abnormal morphology, with generally reduced size, a hook-like shape and asymmetrical growth (Fig. 3i, j). Additionally, we observed local enamel defects and reduced dental pulp chamber filled with dentinous tissue (Fig. 3j). Interestingly, the apical part of affected incisor was shifted anteriorly, starting at the level of the first molar instead of its physiological posterior position close to the temporomandibular joint (Fig. 3j). In contrast to the incisors, the molars of *Wnt1*^*Cre*^*/Csf1*^*fl/fl*^ animals exhibited a less drastic phenotype, with mild morphological alterations of root and crown. Notably, in a single case of the *Wnt1*^*Cre*^*/Csf1*^*fl/fl*^ animals, a supernumerary tooth was observed (Fig. 3j). Variability of all observed dental phenotypes of incisors and molars is summarized in detail in the Supplementary Tables 1 and 2.

Besides the defects in hard matrices, the induced absence of macrophages triggered severe morphological abnormalities in both epithelial and mesenchymal compartments of the continuously growing incisors (Fig. 3k, l, and Supplementary Fig. 4a, b). Histological analysis of the apical tooth region uncovered various defects affecting both teeth and associated structures. This included (i) misshapen and smaller epithelial LaCL, (ii) increased vasculature in the neurovascular bundle region and dental follicle or (iii) discontinuation of pre-ameloblast and ameloblast layers resulting in ectopic folding of the epithelial layer (Fig. 3k, l, and Supplementary Fig. 4a, b). In the developing molars, we observed increased distance between the residual enamel epithelium and the oral epithelium (Supplementary Fig. 4c, d). Our findings indicate that *Wnt1*^*Cre*^*/Csf1*^*fl/fl*^ mutant mice display delayed eruption yet show no defects in epithelial formation or maintenance during the pre-eruptive stages.

**Fig. 4:**
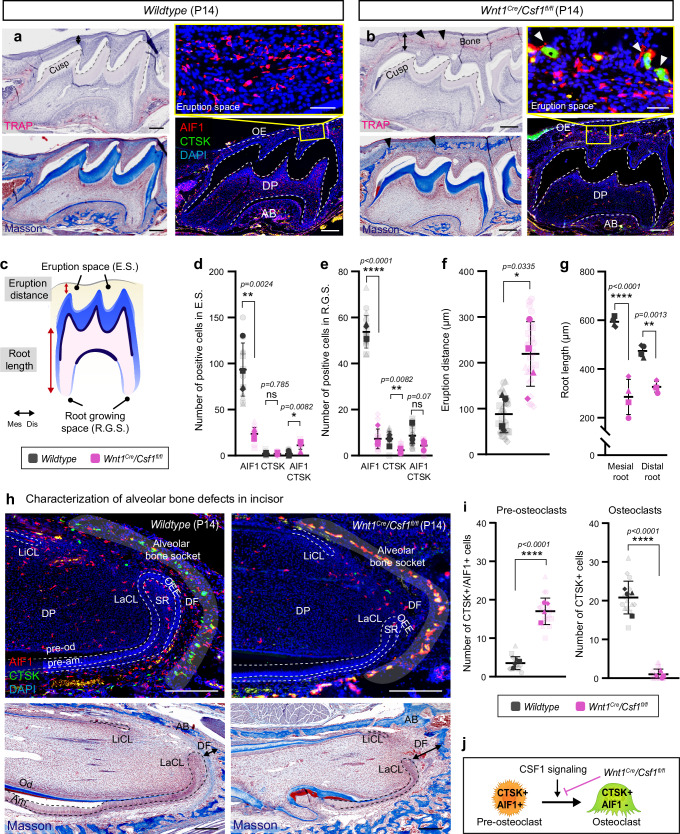
Wnt1^Cre^/Csf1^fl/fl^ mice exhibit delayed molar eruption, delayed root elongation and alveolar bone resorption-associated defects. Molar eruption in *Wnt1*^*Cre*^*/Csf1*^*fl/fl*^ animals is delayed, evidenced by remnants of bone above the molar cusps (blue, Masson´s Trichrome staining) undergoing resorption by osteoclasts in the eruption space marked by Cathepsin K (CTSK+) (white arrowheads) which osteolytic activity is emphasized by TRAP staining (pink signal, marked by black arrowheads) (**a**, **b**). Schematic view of molar P14 (**c**), followed by quantification of number of single AIF1+ cells, single CTSK+ cells and double-positive AIF1+/CTSK+ cells in the eruption space (E.S.) (**d**) and in the root growing space (R.G.S.) (*n* = 4 animals per genotype condition represented by different symbols, all females) (**e**). The measured distance of the eruption space reveals delayed molar eruption (**f**), and root length is significantly reduced (*n* = 4 animals per genotype condition, all females) (**g**). Alveolar bone is impacted by *Csf1* perturbation, resulting in failure to properly resorb the alveolar bone socket, evidenced by a widened of the dental follicle space (black arrows) and the presence of increased nubmer of AIF1+/CTSK+ (pre-osteoclast) cells than only CTSK+ (mature osteoclasts) cells (**h**), with quantification shown in **i** (*n* = 4 animals per genotype condition represented by different symbols, equally distributed by sex). **d–g**, **i** Data are presented as mean ± SD. Statistical significance was assessed using a two-sided unpaired Student’s *t*-test. Exact *P* values are shown in the graphs; ^*^P < 0.05, ^**^P < 0.01, ^***^P < 0.001, ^****^P < 0.0001. Schematic representation of CSF1 signaling guiding correct osteoclast differentiation and function (**j**). Scale bars (**a**, **b**, **h**) = 250 µm, insets = 50 µm. AB alveolar bone, Am. ameloblasts, DF dental follicle, DP dental pulp, LaCL labial cervical loop, LiCL lingual cervical loop, Od. odontoblasts, OE oral epithelium, OEE outer enamel epithelium, pre-am pre-ameloblasts, pre-od pre-odontoblasts, SR stellate reticulum.

In addition, *Wnt1*^*Cre*^*/Csf1*^*fl/fl*^ mutants display an affected bone resorption (Fig. 3i) phenotype. Bone wall thickness analysis (microCT data) revealed that the mandibular alveolar bone was thicker in *Wnt1*^*Cre*^*/Csf1*^*fl/fl*^ mice, suggesting altered osteoclast activity. To further elucidate this phenotype, we conducted a detailed characterization of the jawbones in these animals (Fig. 4). Molar eruption and root formation were developmentally delayed (Fig. 4a, b). To quantify these defects, we measured the number of AIF1+, CTSK+ and AIF1+/CTSK+ positive cells in the eruption space and root growth space (Fig. 4c–e), as well as morphometric parameters including eruption distance and root length (Fig. 4f, g). Our results show a decrease of AIF1+ cells (macrophages) in mutant mice; however, no significant difference was observed in the number of CTSK+ (osteoclasts) or AIF1+/CTSK+ (pre-osteoclasts) cells between mutant and wild-type animals (Fig. 4d, e). The observed delayed molar eruption and root elongation are a direct bone consequence, as disrupting Csf1 signaling impaired correct osteoclast differentiation and slowed bone resorption compared to controls. Subsequently, we examined the alveolar bone socket of the mouse incisor. In contrast to molars, the incisors of mutant mice exhibited a significant increase of AIF1+/CTSK+ cells (pre-osteoclasts) and in parallel a significant decrease of CTSK+ cells (osteoclasts) (Fig. 4h, i) along with a notably wider dental follicle space (Masson Trichrome images of Fig. 4h). These findings explain more prominent phenotype observed in incisors compared to molars (Fig. 3i, j) and suggest the important role of CSF1 signaling for proper differentiation of osteoclasts^57,58^ (Fig. 4j). Thus, perturbations in bone resorption during tooth development can influence its correct formation.

To study if the observed incisor elongation abnormalities are a secondary cause of disrupting osteoblast lineage or early bone matrix deposition, we evaluated matrix secretion on *Wnt1*^*Cre*^*/Csf1*^*fl/fl*^ embryos at E14.5 by Masson Trichrome staining (Supplementary Fig. 5a–c). The extent of collagen-rich osteoid deposition and spatial organization of early bone matrix were not visibly changed. Complementary, we detected RNA in situ hybridization of *Sp7* (Osterix), a key transcription factor for osteoblast commitment and differentiation, and did not reveal altered patterning of the osteogenic domain (Supplementary Fig. 5d, e). Therefore, Csf1 deletion in neural crest–derivatives does not disrupt the establishment of the osteoblast lineage or early bone matrix deposition at the developmental stage preceding incisor elongation

### Bone marrow transplantation partially rescues dental phenotype of *Csf1r* knockout

**Fig. 5:**
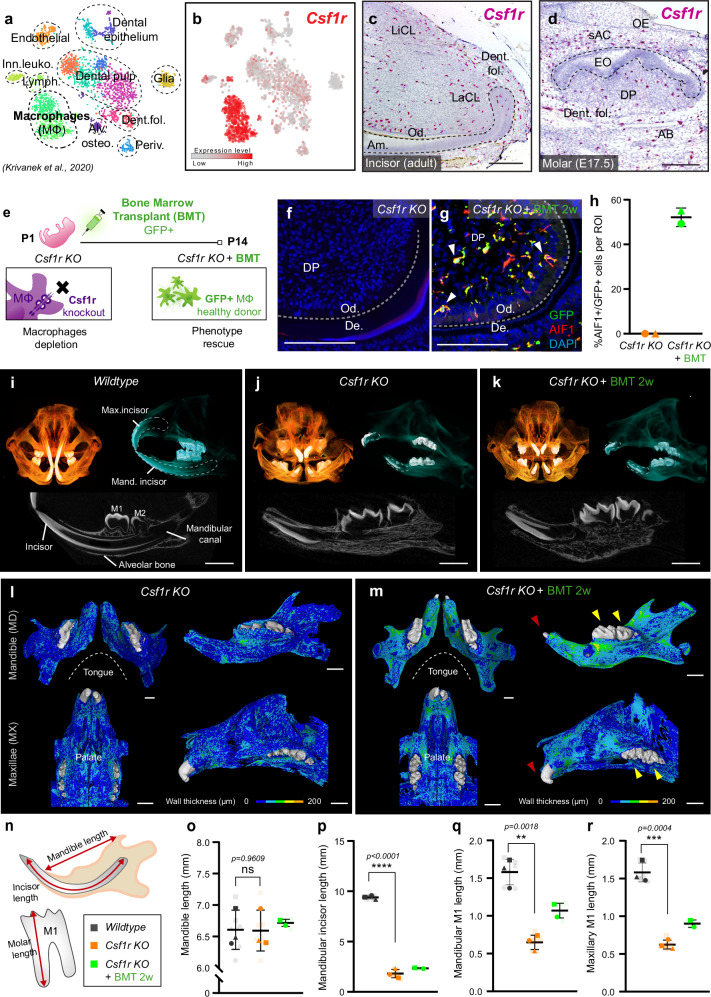
Csf1r KO induced depletion of macrophages exhibits dental phenotype, partially rescued by bone marrow transplantation. Single-cell RNA-seq analysis from adult mouse incisor dataset shows that whole macrophages cluster in teeth is positive for *Csf1r* (**a**, **b**). In situ hybridization proves presence of *Csf1r-*positive macrophages in adult incisors and developing molars (**c**, **d**). Experimental design for bone marrow transplantation (BMT) rescue with GFP+ macrophages from a healthy mouse donor (**e**), followed by immunostaining verification of depleted macrophages in *Csf1r* KO and repletion of macrophages (AIF1) that co-localize with GFP+ donor cells (**f**, **g**), with quantification shown in **h** (*n* = 2 mice per condition, females). MicroCT scans of *Csf1r KO* reveal a severe dental and bone phenotype (**i**, **j**), which is partially rescued by the BMT from a healthy mouse donor performed at P0 and analyzed two weeks (2w) later (**k**). 3D-visualizations from microCT scans show thickening of mandibular and maxillary jawbones, along with improved eruption of incisor (red arrowhead) and molars (yellow arrowhead) following transplantation (**l**, **m**). Rescue phenotype was assessed via morphometric analysis of the schematized areas (**n**), including mandible size (**o**), mandibular incisor length (**p**) and lengths of the mandibular and maxillary molars (**q**, **r**) (*n* = 3 animals wildtype and *Csf1r KO* [sex distribution: 2 females, 1 males], 2 animals BMT rescued [sex distribution: both females], represented by different symbols). Data are presented as mean ± SD. Statistical significance was assessed between WT and Csf1r KO groups using a two-sided unpaired Student’s *t*-test. Exact *P* values are shown in the graphs; ^*^P < 0.05, ^**^P < 0.01, ^***^P < 0.001, ^****^P < 0.0001. No statistical testing was performed for the BMT-rescued group because *n* < 3. Scale bars (**c**, **d**, **f**, **g**) = 100 µm; (**i**–**m**) = 1 mm. Alv. Osteo. alveolar osteocytes, Am. ameloblasts, Dent. fol.dental follicle, DP = dental pulp, De. dentin, M1/2 molar 1/2, Inn. leuko. innate leukocytes, LaCL labial cervical loop, LiCL lingual cervical loop, Lymph. lymphocytes, Mand. mandibular, Max. maxillary, Od. odontoblasts, OE = oral epithelium, Periv. perivascular cells.

Next, we examined changes in tooth development by using a global *Csf1-receptor* knockout (*Csf1r* KO) animal (Fig. 5). Analysis of scRNA-seq data suggested strong and very specific expression of *Csf1r* in the macrophage cluster (Fig. 5a, b). To prove this pattern, we conducted ISH validation and demonstrated the presence of *Csf1r*+ macrophages in both the apical part of the incisor and the developing molar (Fig. 5c, d). To investigate the effect of ablation of macrophages and to highlight the importance of *Csf1/Csf1r* pathway for dental macrophages, we examined *Csf1r* knockout mice (*Csfr1 KO*) (Fig. 5e–h). IHC analysis of teeth from *Csfr1 KO* animals showed a complete loss of AIF1+ macrophages in this tissue (Fig. 5f). This loss resulted in various morphological defects associated with tooth development and growth, further supporting the hypothesis that macrophages play a critical role in tooth formation (Fig. 3i–k). The phenotype of *Csf1r KO* animals (2 weeks old) resembled several features observed in the *Wnt1*^*Cre*^*/Csf1*^*fl/fl*^ mice, including the “hook-like” shape of incisors, flattened molar cusps, malformed incisor growth, and delayed initiation of incisor formation beyond M1 with delayed eruption compared to wildtypes (Fig. 5i–k).

Next, we examined the epithelial alterations observed in *Csf1r KO* animals. Histological analysis of the incisors revealed multiple folded dental epithelium (Supplementary Fig. 4e) that retained the expression CALB1, characteristic marker of differentiating ameloblasts^59^ (Supplementary Fig. 4f). In the unerupted molars of *Csf1r KO* mice, we observed a hypervascularized superior wall of the alveolar crypt and a disorganized epithelial structure above the developing cusps (Supplementary Fig. 4g). These epithelial abnormalities in both the continuously growing incisors and molars were associated with a marked reduction in enamel formation in mutant mice (Supplementary Fig. 4h, i).

Young *Csf1r KO* animals (in contrast to the data for *Wnt1*^*Cre*^*/Csf1*^*fl/fl*^ adult ones) were used to cover the whole developmental process of teeth to match the phenotype rescue experiment explained below. To demonstrate that the absence of macrophages is the causative factor leading to malformations of teeth, we performed a phenotype rescue experiment. We conducted a bone marrow transplantation (BMT) via single intraperitoneal (*i.p*.) administration of healthy mouse GFP+ whole bone marrow into P1 mice (Fig. 5e–h). To assess the success rate of BMT in relation to dental tissue, we quantified the engraftment of the transplanted cells by IHC co-staining of AIF1 and GFP. Although in the dental tissue of *Csf1r KO* animals we did not observe any AIF1+/GFP+ cells, after BMT the number of AIF1+/GFP+ cells increased to 52.15 ± 4.13 cells per region of interest (ROI) in the dental pulp. This demonstrated the efficient infiltration and establishment of transplanted cells within the dental pulp and their contribution to the phenotype rescue (Fig. 5h).

Further analysis by microCT demonstrated partial dental and bone phenotype rescue by 14 days (Fig. 5l, m), which included recovery of molars and incisors eruption and a general restoration of mandibular and maxillary bone thickness in BMT compared *Csf1r KO* animals. Furthermore, to assess morphometric features of wildtype versus *Csf1r KO* and *Csf1r KO* + BMT animals, we measured the mandibular, molar and incisor lengths (Fig. 5n–r). Although the overall mandibular length remained unchanged among the three groups (Fig. 5o), the incisor length showed a slight but significant increase following BMT (Fig. 5p). Notably, the first maxillary and mandibular molars displayed a significant and more pronounced increase in length in *Csf1r KO* + BMT mice compared *to Csf1r KO* controls (Fig. 5q, r). Data obtained from a phenotype rescue experiment thus show that BMT-derived cells promptly reach the dental tissues and provide in situ support to save a phenotype of malformed teeth.

To reconcile the phenotypic variations observed between the conditional knockout (*Wnt1*^*Cre*^*/Csf1*^*fl/fl*^*)* and the global knockout *(Csf1r KO)*, we summarized the findings for incisors and molars in Supplementary Table 1 and Supplementary Table 2, respectively. Overall, both variations of genetic ablation of macrophages with subsequent phenotype rescue experiments indicate the important role of macrophages for proper development, growth and patterning of teeth and their close surroundings.

To address the implication of macrophage depletion during mandibular alveolar bone formation, we performed morphometric analyzes using microCT datasets and quantitatively assessed mandibular anteroposterior lengths and vertical heights in control, *Wnt1*^*Cre*^*/Csf1*^*fl/fl*^ and *Csf1R KO* animals. Three-dimensional microCT reconstructions showed that the overall mandibular organization is preserved in *Wnt1*^*Cre*^*/Csf1*^*fl/fl*^ mutants, with clearly identifiable alveolar regions (incisor and molar alveoli), a properly positioned mandibular ramus, and formation of the major coronoid, condylar, and angular processes (Figs. 3i, j and 5l, m). To quantitatively assess the changes in the mandibles of *Wnt1*^*Cre*^*/Csf1*^*fl/fl*^ and *Csf1R KO* animals, we performed quantitative morphometric analyzes using defined anatomical landmarks (Supplementary Fig. 5f–i). The measurements of *Wnt1*^*Cre*^*/Csf1*^*fl/fl*^ animals (both 6-weeks or 8-weeks old animals) showed minor, however statistically significant differences in the anteroposterior dimensions. For 6-weeks *Wnt1*^*Cre*^*/Csf1*^*fl/fl*^ mutants, A-B and A-C distances were 7.92 ± 0.17 mm and 6.21 ± 0.19 mm, respectively; and for controls, 8.57 ± 0.62 mm and 7.09 ± 0.13 mm. In contrast, no statistically significant differences were observed in the vertical height. For 6-week-old *Wnt1*^*Cre*^*/Csf1*^*fl/fl*^ mutants, B-C and D-E distances were 4.65 ± 0.04 mm and 2.86 ± 0.06 mm, respectively; and for controls 5.22 ± 0.62 mm and 3.32 ± 0.23 mm (Supplementary Fig. 5g, h). On the other hand, 2-week-old *Csf1R KO* animals exhibit pronounced variations in mandibular sizes, affecting both anteroposterior and vertical dimensions. Anteroposterior A-B and A-C distances in *Csf1R KO* jaws were 6.14 ± 0.22 mm and 5.21 ± 0.47 mm compared to age-matched controls, which displayed 6.94 ± 0.14 mm and 6.77 ± 0.10 mm. Vertical dimensions B-C and D-E distances in *Csf1R KO* jaws were 3.45 ± 0.31 mm and 2.41 ± 0.13 mm compared to controls which sized 4.02 ± 0.06 mm and 2.55 ± 0.05 mm (Supplementary Fig. 5i). Together, our data indicates that disruption of Csf1-Csf1r signaling axis can impact the mandible size, which is rather minor in *Wnt1*^*Cre*^*/Csf1*^*fl/fl*^ animals and more notable in *Csf1R KO* animals.

### Pharmacological depletion of macrophages in adults leads to reversible disruption of dentin and enamel formation in continuously-growing teeth

Clodronate liposomes (belonging to a larger group of bisphosphonates) are widely used drugs that specifically eliminate macrophages and some of their tissue derivatives in living organisms^60,61^. This drug is engulfed by metabolically active phagocytic cells, such as macrophages, and converted into a toxic ATP analogue that inhibits mitochondrial translocase, resulting in cell death^62,63^.

**Fig. 6:**
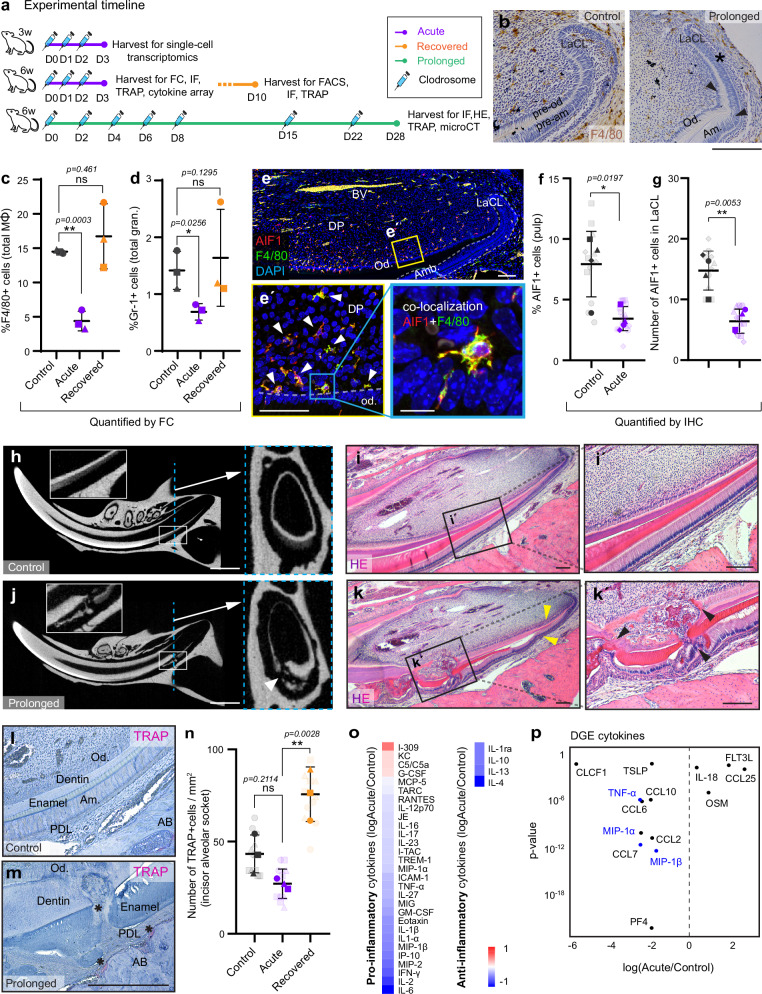
Pharmacological disruption of macrophage homeostasis causes reversible defects in continuously growing teeth. Experimental design for bisphosphonates administration and tissue collection is shown in (**a**). Immunohistochemistry for F4/80+ demonstrates reduced macrophages in the tooth apical region, abnormalities of epithelial labial cervical loop (asterisk), and altered (pre-)odontoblast and (pre-)ameloblast morphology (arrowheads) (**b**). Flow cytometry revealed reduced F4/80+ macrophages (**c**) and Gr-1+ granulocytes (**d**) in pulps of control, acute and recovered conditions. Each symbol represents one pooled pulp sample from two mice (mixed sex, *n* = 3 samples per condition). Data are mean ± SD. Statistical significance was determined by two-sided one-way ANOVA with Tukey’s multiple-comparisons test. AIF1 and F4/80 co-staining confirm marker co-localization in tooth macrophages (**e**, **e´**). Immunofluorescence quantification showed reduced AIF1+ cells after acute treatment in dental pulp (**f**) and epithelial compartment (**g**). Each symbol represents one animal (*n* = 3-4 males per condition). Data are mean ± SD. Statistical significance was assessed using a two-sided unpaired Student’s *t*-test. Bisphosphonates disrupted macrophage homeostasis and impaired dentin and enamel visible from microCT scans (white arrowhead) and hematoxylin and eosin (HE) staining (black arrowheads) (**h**–**k**). These defects were resolved after treatment withdrawal (yellow arrowheads). Absence of TRAP+ osteoclasts in the dentin-enamel lesion interphase after prolonged bisphosphonates indicates that the defect is not driven by elevated osteoclast activity (**l**, **m**). Recovery after clodrosome was associated with osteoclast activity in the incisor alveolar bone socket (**n**). Each symbol represents one animal (*n* = 3 per condition; 2 females and 1 male). Data are mean ± SD. Statistical significance was determined by two-sided one-way ANOVA with Tukey’s multiple-comparisons test. *P* values are shown; ^*^P < 0.05, ^**^P < 0.01, ^***^P < 0.001, ^****^P < 0.0001. Cytokine array revealed downregulation of both pro-inflammatory and anti-inflammatory factors (**o**), consistent with differential gene expression analysis of single-cell RNA-sequencing data from clodrosome-treated animals *versus* controls (**p**). Scale bars: (**e**, **i**, **k**–**m**) = 100 µm; (**h**, **j**) = 1 mm. AB alveolar bone, Am. ameloblasts, BV blood vessels, DP dental pulp, LaCL labial cervical loop, Od. odontoblasts, PDL periodontal ligament, pre-am. pre-ameloblasts, pre-od. pre-odontoblasts.

To investigate the role of macrophages in the growth of continuously developing incisors in fully adult organisms, we *i.p*. administered Clodrosome^®^ (liposome-encapsulated form of bisphosphonate clodronate drug) to pharmacological ablate macrophages (Fig. 6). To investigate the role of macrophages in maintaining incisor homeostasis, we implemented different Clodrosome delivery regimens and examined the dental tissue adaptive response following macrophage depletion (Fig. 6a). Clodrosome was delivered at specific time intervals to understand macrophages contribution upon different conditions: (1) acute response where tissue was harvested 1 day after 3 consecutive days of administration, (2) recovered status where tissue was harvested 1 week after 3 consecutive days of administration, (3) long-term effect where tissue was harvested after pulsed administration every other day for a week, and maintained once a week for 3 more weeks (Fig. 6a).

Immunohistochemical and flow cytometric (FC) analyzes confirmed partial depletion of macrophages following Clodrosome treatment, with a recovery of macrophage numbers after a 1-week break from Clodrosome treatment (Fig. 6b–g). Validation was based on the detection of the surface macrophage marker F4/80 and the intracellular marker AIF1, which were found to co-localize within the same cell population (Fig. 6e, and Supplementary Fig. 2d). Immunostaining of F4/80 further supported a reduction of F4/80+ cells in the dental mesenchyme surrounding the cervical loop, as well as in the stellate reticulum of dental epithelium (Fig. 6b). We observed morphologically abnormal shape of LaCL with prematurely differentiated odontoblasts and ameloblasts in Clodrosome-treated condition (Fig. 6b). In acute response experiment we detected a significant drop of amount of F4/80+ cells from 15.23 ± 0.25% (Mean ± standard deviation) (untreated condition) to 4.97 ± 1.46% in acute condition. The ratio of F4/80+ macrophages was restored after the recovery period to 17.5 ± 4.81% 1 week after the last Clodrosome delivery (Fig. 6c). Additionally, we analyzed the ratio of Gr-1+ cells (a common marker of granulocytes, differentiating monocytes and macrophages) after the Clodrosome treatment. FC analysis showed a similar trend to that observed for F4/80 with ratio of Gr-1+ cells of 1,43 ± 0.35% in controls, 0.66% in acute-response and 1.66% in the recovered group (Fig. 6d). Likewise, quantification of AIF1 expression by immunohistochemistry on serial paraffin sections revealed a reduced number of AIF1+ cells in both the dental pulp (Fig. 6f) and the epithelial compartment of LaCL (Fig. 6g).

To assess the effect of transient macrophage depletion on the structure of dental mineralized tissues, we performed microCT analysis. Our results revealed a transient disruption of dentin and enamel (Fig. 6h, j), which we further showed in detail at the histological level (Fig. 6j, k) by discontinuous odontoblast and ameloblast layers, creating anisotropically deposited dentin specifically in the incisor.

Notably, the transient effect of macrophage depletion was specifically observed after prolonged Clodrosome treatment followed by 1-week recovery time (Fig. 6k). The impacted area was located approximately in distance of 1.5 mm from the LaCL. This distance corresponds to a weekly growth rate of mouse incisor, which was previously shown to be around 1.61 mm in adult mice^64^. Following cessation of treatment (days 22–28), the physiological growth and tissue morphology were completely restored, resulting in restoration of the odontoblast and ameloblast layer morphology and the dentin and enamel formation (Fig. 6k). This suggests a reversible effect of macrophage depletion and a remarkable buffering capacity of these cells to restore tissue homeostasis. Importantly, there is no evidence that other tooth-resident cells, such as dental pulp cells or odontoblasts, internalize or resorb Clodrosome, supporting the specificity of this approach for targeting macrophages within the tooth.

Clodronate is commonly used to treat diseases associated with improper bone metabolism, whereby its mechanism of action is to affect osteoclasts, cells derived from macrophages that have the ability to resorb hard tissue. To exclude the association of observed dental lesions with ectopic activation of osteoclasts, we performed TRAP (Tartrate-resistant acid phosphatase) assay to detect the osteolytic activity of osteoclasts (Fig. 6l–n). Osteoclasts in association with the alveolar bone of the continuously growing incisor are physiologically associated with permanent growth and associated periodontal tissue remodeling^65^. We detected a normal distribution of osteoclasts in this region and a complete absence of any TRAP+ cells or traces of TRAP staining (both indicating osteoclast activity)^66^ at the site of the enamel and dentin damage (Fig. 6l, m). Thus, the lesion is presumed to have been formed during dentin and enamel patterning and not secondarily. To further investigate this, we analyzed the dynamics of osteoclast activity in the jawbone following Clodrosome administration by assessing TRAP+ staining in the incisor alveolar bone socket. While acute treatment caused a slight but insignificant reduction of osteoclast activity, a significant increase was observed 1-week after treatment cessation (Fig. 6n). The absence of changes in osteoclast activity at short-term Clodrosome administration is consistent with previous studies showing that clodronates primarily inhibit osteoclast function over prolonged treatment rather than immediately^67,68^. This delayed response likely reflects a compensatory upregulation of osteoclast activity during the recovery phase. Importantly, given the relatively slow turnover of alveolar bone in adult animals and the absence of detectable bone defects around incisors at the time of analysis, these findings further support a tooth-autonomous origin of the observed lesions, consistent with the rapid self-renewal dynamics of the incisor

Bisphosphonate administration has been reported to influence the production of both pro- and anti-inflammatory cytokines, likely through apoptotic macrophage clearance and subsequent immune modulation. In the short term, bisphosphonates reduce pro-inflammatory cytokine production, whereas in the long term, this effect is reversed^69^. To investigate this phenomenon in our Clodrosome-treated mice, we employed two complementary approaches. First, pro- and anti-inflammatory cytokine levels were analyzed in isolated dental pulps of incisors using a targeted array profiling. This analysis revealed an overall downregulation of both cytokine classes (Fig. 6o). We then correlated these findings with scRNA-seq transcriptomic data from Clodrosome-treated mice, which similarly showed downregulation of key pro-inflammatory markers, including MIP1β, MIP1α, and TNFα (Fig. 6p), which was consistent with the cytokine assay at the protein level.

**Fig. 7:**
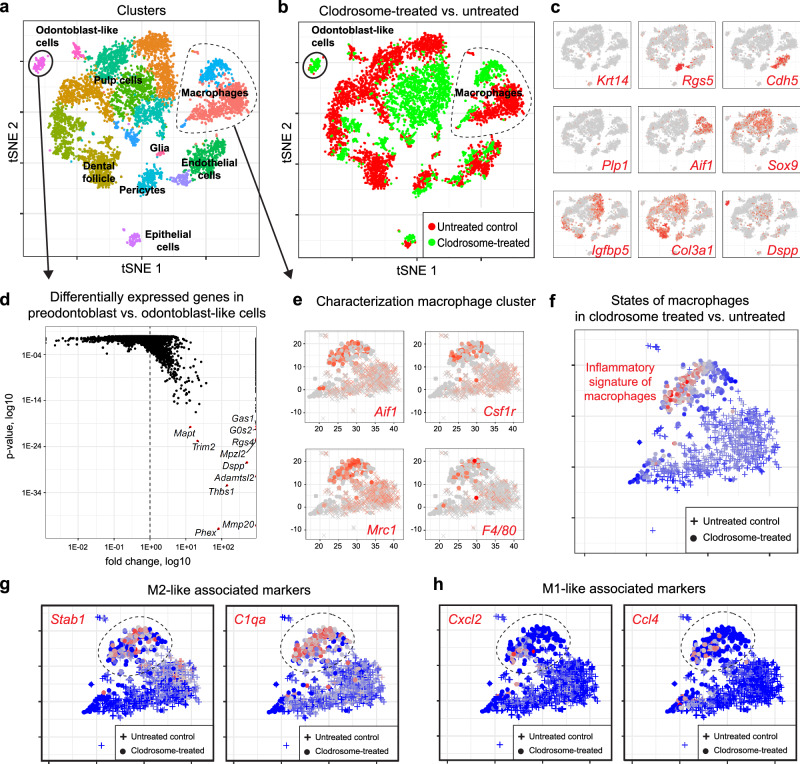
Phenotype change of dental macrophages after bisphosphonates treatment analyzed by single-cell RNA-sequencing. Projection of identified dental cell clusters into tSNE plot (**a**), sorting of populations based on clodrosome-treated or untreated conditions (**b**). Panel of characterization of cell clusters by identification of typical markers (**c**). Analysis on Clodrosome-treated mice incisor revealed an emerging odontoblast-like cell population expressing differential gene expression (**d**). Analysis of macrophage cluster characterized by general macrophage markers (**e**) reveals a change in the state of macrophages after clodrosome administration (**f**), featured by acquisition of M2-like phenotype potentially responsible for the tissue healing (**g**) when compared with M1-like phenotype markers (**h**).

Finally, to better understand the origin of the observed temporary dental phenotype at the cellular and molecular levels, we conducted scRNA-seq analysis of clodronate-treated and non-treated (Fig. 7a–c). Characterization of cellular composition showed an increased presence of cells with an odontoblast-like phenotype (*Dspp*+, *Phex*+) in the Clodrosome-treated condition (Fig. 7d). This result correlates with the regionalized, immature and distorted dentin structure (Fig. 6h–k) where we observed higher numbers of odontoblast-like cells. These cells did not yet fully develop odontoblast processes, anchoring them in dentin matrix and thus can be more easily isolated from dentin^70^.

**Fig. 8:**
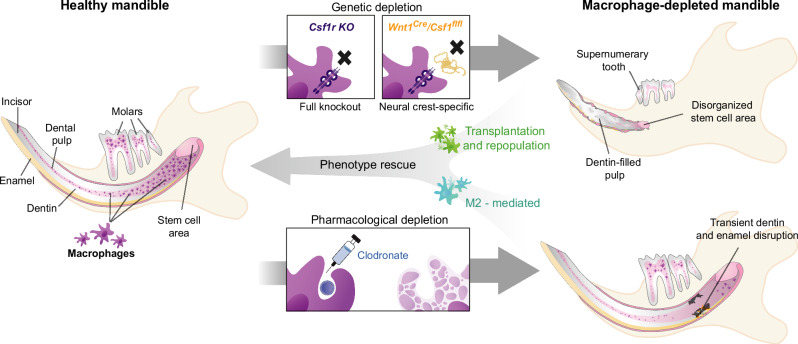
Graphical abstract. Macrophages populate developing dental tissues and remain associated with epithelial and mesenchymal stem cell niches throughout life. Their genetic or pharmacological depletion disrupts tooth patterning, dentin and enamel formation, and eruption, whereas macrophage repopulation promotes tissue restoration through an M2-associated reparative response. These findings identify macrophages as essential regulators of tooth development, homeostasis, and regeneration.

Finally, and most importantly, scRNA-seq analysis revealed a phenotype shift of macrophages in the macrophage cluster (Fig. 7e) from M0-like (resting) into M2-like (healing) after acute-Clodrosome treatment. This newly emerged macrophage subset (Fig. 7f) exhibited elevated expression of M2-like-associated markers, including *Stab1*, *C1qa* genes, rather than M1-like (*Cxcl2* and *Ccl4* genes) (Fig. 7g, h). These findings indicate rapid adaptive response abilities of macrophages, which instantly react to dental tissue damage and facilitate a fast recovery of homeostasis, thereby restoring function of the incisor — an organ critical for the survival of this species (Fig. 8).

## Discussion

Teeth are vital organs that have undergone various evolutionary modifications to accommodate diverse feeding strategies and ecological adaptations. In mice (and many other species), this evolutionary process has resulted in the acquisition of continuously growing teeth (incisors) that provide an excellent model to study development, regeneration, and stem cell niche behavior^71,72^. Tooth morphogenesis initiates during early embryonic development and continues postnatally, being a highly orchestrated event resulting in the acquisition of several tooth-specific cell types and comprehensively organized hard tissues^73^.

Throughout these developmental stages, tooth macrophages emerge as an abundant component of the tooth microenvironment. Although macrophages are well recognized for their roles in immune surveillance and tissue homeostasis in various organs^1,74,75^, their specific contribution to dental morphogenesis has remained unexplored. Recently, macrophages have been identified as critical regulators of these developmental functions in other organs^1,74,75^. In this work, we uncovered a previously understudied area of macrophages as regulators of tooth development, patterning and eruption, expanding their known role in adaptive immune function under dental trauma and infection^76–79^.

We observed macrophages as early as the bud stage (E12.5) of tooth development, localized exclusively within the dental mesenchyme but absent from the epithelium. During the development through the late bud, cap, and bell stages (E13.5–E17.5), macrophages increasingly populated the dental papilla, follicle, and enamel organ. Interestingly, macrophage abundance increased markedly after birth, resembling patterns seen in other organ systems^80^. However, further lineage-tracing studies will be required to confirm this relationship.

In the incisor, we additionally mapped macrophage distribution according to specific regions and associations to diverse cell populations. We identified circulating macrophages associated with blood vessels, proliferating tissue macrophages in dental pulp, macrophages interspersed among odontoblasts, those occupying the dental follicle, and macrophages that migrated into the dental epithelium—particularly in the labial cervical loop where the epithelial stem cell niche is located^49,72^. The compartment-specific distribution implies that macrophage functions are context-dependent, modulating the microenvironment according to the local developmental or regenerative demands of each region.

Noticeably, we observed that macrophages are in direct physical contact with transient dental epithelial progenitors. We hypothesize that macrophages play a role in this particular location, maintaining stem cell niche homeostasis, as suggested in other tissues in recent studies^11^. The mechanisms by which macrophages can exert their influence may be either in providing support for proliferation^81^, producing growth factors (such as VEGFs)^82^, or regulating inflammation^9^. Ligand–receptor analysis identified the macrophage Lgals9 ligand and epithelial CD44 receptor as a putative communication axis, suggesting that macrophage–epithelium crosstalk may mediate the regulation of proliferation and differentiation. However, while this data provides an initial insight into macrophage-mediated signaling within the epithelial niche, the precise mechanism by which macrophages influence surrounding epithelial or mesenchymal stem cell niches to ensure proper tooth development remains to be investigated in future studies.

To determine whether macrophages play a causal role in dental development, we employed both global and tissue-specific genetic ablation models. Firstly, we genetically ablated macrophages using neural crest-targeted conditional knockout of *Csf1*^*fl/fl*^ ligand by crossbreeding with *Wnt1*^*Cre*^ driver. The teeth of *Wnt1*^*Cre*^*/Csf1*^*fl/fl*^ animals lacked macrophages in both the mesenchymal and epithelial compartments, despite *Wnt1* expression being localized exclusively to the mesenchymal part (neural-crest origin). The absence of macrophages in the epithelial compartment correlates with our findings that macrophages first populate mesenchyme-related areas of the tooth and only later migrate into the enamel-forming dental epithelium. Consequently, the disruption the *Csf1* signaling affects the initial stages of tooth development when macrophages populate dental mesenchyme and results in a complete loss of macrophages even in the adjacent epithelial areas. Innate genetically-induced depletion of macrophages using *Wnt1*^*Cre*^*/Csf1*^*fl/fl*^ animals resulted in a robust dental phenotype. Although molars were able to erupt, they exhibited various morphological aberrations, such as larger pulp and shorter roots. Interestingly, throughout evolution, only three molars and a single incisor remained in each quadrant of mouse jaws and other teeth disappeared^83^. The current shape of the first molar of mice is a result of evolutionary fusion of the ancestral first molar and second premolar^84,85^. *Wnt1*^*Cre*^*/Csf1*^*fl/fl*^ genotype, in a single case, a supernumerary tooth was positioned mesially from the first molar. As induction of tooth germs is prior to bone formation in embryogenesis, the presence of a supernumerary tooth provides evidence of a tooth-autonomous phenotype stemming from impaired early tooth patterning. These results show that macrophages are not only present from early tooth development, but they also play a critical role in early tooth patterning^83^.

The mechanism by which macrophages control tooth development could involve their phagocytic activity, which may be either specifically or non-specifically targeted on recycling signaling molecules from the *Wnt*, *Bmp* or *Fgf* signaling pathways that are essential for tooth morphogenesis^86–89^. Importantly, the observed phenotype is not attributable to the *Wnt1*^*Cre*^ background itself, as animals lacking *Csf1*^*fl/fl*^ allele do not exhibit any dental defects^90^.

It is worth noting that *Wnt1*^*Cre*^*/Csf1*^*fl/fl*^ animals also exhibited additional craniofacial abnormalities. While the overall architecture and size of the upper and lower jaws were preserved, the bone displayed increased thickness, likely resulting from impaired osteoclast differentiation and function. The delayed molar eruption observed in *Wnt1*^*Cre*^*/Csf1*^*fl/fl*^ mice aligns with previous studies demonstrating that local administration of CSF-1 accelerates tooth eruption and modulates bone remodeling through osteoclastogenesis^25,91^. CSF-1 plays a critical role in supporting the recruitment of mononuclear cells (osteoclast precursors) to the dental follicle prior to the onset of eruption^23,92^. These findings emphasize the importance of CSF-1 signaling for the coordinated interaction between the dental follicle and the surrounding bone to achieve correct tooth shape. This finding is linked to the neural crest origin of the majority of cranial bones^93^. From this perspective, the dental phenotype observed in *Wnt1*^*Cre*^*/Csf1*^*fl/fl*^ animals should not be considered as an isolated defect, but rather as a part of a broader phenotypic manifestation. However, our data indicate that dental defects arise as a primary disorder rather than as a secondary effect of surrounding bone pathology.

Global knockout of the critical macrophage survival receptor (*Csf1r KO*) confirms the necessity of macrophages for proper dental development. Typically, *Csf1r KO* animals do not survive beyond the third week of postnatal development due to malocclusion-related feeding difficulties and neurologic deficits due to lack of brain microglia^94,95^. We showed that *Csf1r* can be used as a specific and reliable marker in dental tissue by single-cell transcriptomics data and validated by RNA in situ hybridization, and is critical to promote proliferation, survival and differentiation of macrophages^50,52^. Although *Csf1r KO* animals establish teeth, they display severely dysmorphic tooth phenotypes. Bone marrow transplantation by adoptive transfer replenishes macrophages in both molars and incisors, partially restoring the morphological phenotype, which indicates a cell-autonomous contribution of macrophages. Along with the dental phenotype rescue, the bone marrow transplantation partially compensates for the inefficiency of *Csf1R KO* mice to achieve correct osteoclast differentiation, visible by an overall improvement of the mandibular and maxillary jawbone shape and density.

The phenotypic differences between *Csf1R KO* and *Wnt1*^*Cre*^*/Csf1*^*fl/fl*^ animals can be reconciled by the spatial and functional specificity of CSF1-CSF1R signaling. While global loss of CSF1R disrupts systemic macrophage development and homeostasis, neural crest-specific loss of CSF1 impairs local signaling required for craniofacial bone and tooth development, without affecting other CSF1-producing tissues. Moreover, residual CSF1 or compensatory ligands such as IL-34 may support survival and function of macrophages outside the neural crest-derived domains.

To demonstrate the role of macrophages in adult dentition, we pharmacologically depleted macrophage populations in wild-type adult mice using lipophilic bisphosphonate (Clodrosome). Although bisphosphonates have been proposed as tools for studying the osteoclast-mediated events during tooth eruption, as they delay molar eruption in rats^96^, in our study Clodrosome was administered long after tooth eruption had occurred, when bone was already stable in the short term. This explains why no effects were detected in the bone next to the teeth, and therefore we do not follow this aspect further, focusing primarily on the tooth defects instead of bone. Interestingly, even after repeated Clodrosome administration, macrophages were not fully depleted in dental tissue collected 24 h after the last injection, as confirmed by FC, nor 1 week later via IHC. Despite only partial depletion of AIF1+ macrophages, the prolonged treatment resulted in severe phenotype affecting the morphology of dentin and enamel in continuously growing teeth. Notably, the affected region of the incisor was located in the apical part of the tooth, matching the timing of treatment and the pace of incisor growth^64^. These specific and transient defects in the tooth were exclusively found in incisors and not in molars, since molars are stable and do not self-renew. This highlights that Clodrosome-macrophage-driven defects arose only when cell dynamics of hard matrix generation is fast, and the stem cell niches (epithelial and mesenchymal) are active. This, coupled with our observation of actively proliferating tooth macrophages in adult teeth, suggests that macrophages quickly repopulate impacted dental tissue.

scRNAseq results from Clodrosome-treated animals vs. controls revealed not only the presence of a macrophage population but, more importantly, a phenotype shift from M0-resting macrophages to M2-healing macrophages. The rapid acquisition of M2-like macrophage phenotype in response to tissue damage aligns with previous studies showing that neural cells^97^, human-derived gingival mesenchymal cells^98^, and dental pulp stem cells^99^ promote M2 macrophage polarization to enhance wound healing. In summary, our findings suggest that after injury, the population of tooth macrophages rapidly recovers and activates an M2 healing phenotype, which leads to the swift restoration of healthy tooth morphogenesis. A limitation of this study is that dendritic cells, which also express DC-STAMP, may participate in tooth development. Although our scRNA-seq analysis showed a minuscule population expressing this molecular marker, dendritic cells were not studied more deeply in our work^100^.

In this study, our data indicate that some observed dental defects (e.g., single case appearance of supernumerary teeth or the transient disruption of dentin/enamel formation upon clodrosome treatment) originate primarily in teeth. However, in some other phenotypes (delayed molar eruption and root elongation or incisor shortened morphology), the role of surrounding bone cannot be completely excluded.

The development of alveolar bone and teeth represents a highly integrated and co-regulated process within the embryonic craniofacial complex. These tissues do not form independently but arise through continuous molecular, biomechanical, and immune system-derived interplay^101,102^. Macrophages and their CSF1-dependent lineages emerge as key mediators within this interface, orchestrating osteoclast differentiation, bone resorption, tooth eruption, as well as intra-dental epithelial and mesenchymal cell responses. This intricate coupling means that perturbations affecting bone formation and immune cell recruitment to facial tissues during development are unlikely to remain confined to one compartment. Instead, they propagate across the entire alveolo-dental complex, altering root elongation, eruption dynamics, and even enamel and dentin maturation. Therefore, a mechanistically holistic approach - one that jointly examines odontogenic and osteogenic processes and integrates the contributions of tooth macrophages and stromal cells - is essential for understanding the orchestration of orofacial development. In this specific context, studying bone and tooth formation in isolation risks overlooking the reciprocal dependencies that define this morphogenetic continuum and shape both normal development and pathological outcomes. Overall, our work does not exclude partial bone-dependent effects associated with disruption of Csf1-Csf1r signaling on tooth formation.

At present, it is not possible to clearly delineate whether all the observed phenotypical manifestations of the mutant mice (shorter incisors, pulp chamber filled with dentin, multiple folded or discontinuous dental epithelium, etc.) arise from bone-driven effects, tooth-intrinsic mechanisms, or a combination of both, as current in vivo methodologies do not allow the study of these processes in complete isolation. Further research will be required to disentangle these interdependent mechanisms.

This role of macrophages mirrors their involvement in complex tissue regeneration, for example during salamander limb regrowth or murine ear punch recovery^103,104^. In these regenerative contexts, macrophages emerge as critical agents for developing and maintaining tissue integrity beyond mere inflammation, and their perturbation often results in distorted morphogenesis. Our results provide evidence for the essential role of macrophages in dental tissues and may also highlight their broader importance in other tissues, where they have been shown to regulate permanently active stem cell niches through interactions with myogenic progenitors and by contributing to the maintenance of stem cell environments in the intestine, mammary gland, and hematopoietic system^11–15^. Although our research focused on mice, this model shares a high degree of similarity in tooth development and morphogenesis with humans^105–108^. Our findings suggest that macrophages may serve as potential therapeutic targets in disorders involving tooth malformations, including eruption failure and pulp stone formation. This hypothesis is further supported by the previously reported roles of macrophages in regulating stem cell niches^11^. In addition, specific stimulation of macrophages in the dental pulp might be used to improve the formation of tertiary dentine after tooth injury. This idea stems from previously described function of macrophages in mediating the healing of skin lesions^109^ and their role in activating quiescent stem cells after skeletal muscle injury^12^.

Taken together, our findings using multiple approaches to target macrophages—including genetic manipulation of the CSF signaling pathway (affecting both Csf1 and Csf1r), phenotype rescue experiments, and pharmacological depletion— converge on similar dental manifestations. Our results highlight the crucial role that macrophages play in tooth development and homeostasis. However, the precise mechanisms by which macrophages refine morphogenesis are still enigmatic and are a matter of future studies.

## Methods

### Animals

Animal experiments were approved either by the Ethik-Kommission der MedUni Wien zur Beratung und Begutachtung von Forschungsprojekten am Tier in Austria, Ethical Committee on Animal Experiments (Stockholm North Committee) in Sweden, Ministry of Education, Youth and Sports, Czech Republic (MSMT-6379/2022-4) or IACUC panel of Children’s Hospital of Philadelphia. Clodrosome treatments were conducted at CCRB, King’s College London, under Home Office (Project license number PPL70/7866). Animal experiments were done in accordance with institutional animal care and ethical committees and French and European guidelines for animal care under approval APAFIS#49928-2024061911572138. All mice were kept under SPF conditions. Experiments were performed according to international and local regulations. Mice were housed in 12/12 light/dark cycle, at a temperature ranging from 18 to 23 °C and 40–60% humidity. Food and water were provided for the animals *ad libitum*. Genetically modified or wild-type animals used in this study were *C57BL/6* genetic background. Mice used for all experiments were sacrificed by an isoflurane (Baxter KDG9623) overdose.

### Tissue handling and staining

Mice used for all experiments were sacrificed by an isoflurane (Baxter KDG9623) overdose; mandibles were carefully dissected out, fixed in 4% paraformaldehyde pH 7.4 for 5–15 h, decalcified in 10% EDTA pH 7.4 for 7 days at +4 °C, cryopreserved in 30% sucrose overnight at +4 °C and embedded in OCT medium (Tissue-Tek, 4583) on dry ice. Samples were cut on cryostat (Leica CM1850UV) in sagittal orientation as 14 μm sections. Before antibody staining, antigen retrieval was performed (Dako S1699). Staining with primary antibodies was performed overnight at room temperature followed by Alexa-conjugated secondary antibodies staining at room temperature for 1 h (Invitrogen, 1:1000). Used antibodies: AIF1 (Novus, NB100-1028; 1:500, previously shown as specific for macrophages^110^), COL4 (AbD Serotec, 2150-1470; 1:500), CALB1 (Swant, CB-38a, 1:200), CDH1 (BioTechne, AF748. 1:500), CTSK (ProteinTech, 11239-1-AP, 1:300), F4/80 (Abcam, ab6640; 1:200), GFP (Acris; R1091P, 1:200), MKI67 (Zytomed, RBK027-05, 1:200). Cell nuclei counterstaining was performed with DAPI (Sigma Aldrich, D9542) diluted 1:1000 in PBS + 0.1% Tween 20 (Sigma Aldrich, P9416) and slides were mounted with 87% glycerol (Merck, 104094) or Fluoromount Aqueous Mounting Medium (Sigma-Aldrich, F4680). Imaging was performed using a Zeiss LSM880 laser scanning confocal microscope. ZEN3.4 (ZEISS), Imaris (Bitplane), QuPath0.5.0 and ImageJ (Fiji) software were used for image processing and GraphPad Prism 8 for plots. Conventional histological staining after Clodrosome or Encapsome treatments was performed after 4 weeks of decalcification of dissected mandibles in 10% EDTA. Mandibles were embedded in wax blocks and sectioned using 8 μm thickness. Sections were stained using Masson’s Trichrome. For the TRAP staining, sections of adult incisors were deparaffinized in xylene and rehydrated in graded ethanol. Osteoclasts were identified by staining for tartrate-resistant acid phosphatase (TRAP) activity (Sigma-Aldrich) in 50 mM Na-tartrate, counterstained with haematoxylin and aqueous mounted. Collagen identification by Masson Trichrome staining in sections, deparaffinized in xylene and rehydrated in graded ethanol, using Weigert hematoxylin, differentiation in 3% acid alcohol, hydrated, and subsequently slides were immersed in ponceau acid fuchsin, orange G, aniline blue 2.5%, dried by ethanol series and aqueous mounted.

### Bone marrow transplantation

Bone marrow harvests of GFP-tagged donor cells were completed using adult C57BL/6-Tg (CAG-EGFP)131Osb/LeySopJ (“Osb-GFP,” Jax 006567) mice as previously described^111^, using femurs and tibias flushed with 1X PBS. Whole bone marrow was subsequently *i.p*. injected into *Csf1r* KO mice to perform rescue.

### Cytokine detection assay

Mouse incisor pulp was dissected using a scalpel to break the surrounding bone and the dentin, followed by fine tweezers to scrape out the pulp tissue^70^. Pooled pulps were lysed by using lysis buffer (1% Triton X-100, EDTA-free protease inhibitor cocktail (Roche, 04693132001) in 1X PBS), mechanically homogenized and sonicated. The suspension was spin at 4 °C, 10,000 × *g* for 5 min to discard the cellular debris. Protein concentration was measured using BCA mix kit following the manufacturer guidelines. Quantification of cytokines was performed by using Proteome Profiler Mouse Cytokine Array Kit (Biotechne, ARY006) using 200 µg of protein lysate per membrane.

### Macrophage’s depletion

Adult CD1 wild-type mice were *i.p*. injected with a macrophage depletion kit Clodrosome® and Encapsome® (Encapsula NanoSciences). Clodrosome® is a Liposomal Clodronate, and Encapsome® is the Control Liposomes. Except for macrophages depletion the Clodrosome® (clodronate) belongs to the class of drugs known as bisphosphonates which are widely used for osteoporosis treatments by mechanism of action by removing osteoclasts. Animals were injected with an amount of 200 μl of the bisphosphonate drug per 30 g of body weight. The animals underwent to different regime strategies based on the condition of study: (1) acute: 6 weeks-old mice received 3 consecutives daily *i.p*. injections (200 μl per 30 g of body weight) and harvested 1 day after; (2) recovered: 6 weeks-old mice received 3 consecutives daily *i.p*. injections (200 μl per 30 g of body weight) and harvested 1 week later; (3) prolonged; 6 weeks-old mice underwent a regime of 7 injections over 29 days, every 2 days until day 8 with 100 μl *i.p* and then, weekly with 100 μl for 2 weeks (day 15 and day 22). Exclusively, for single-cell sequencing (10×), 3 weeks-old mice were used being *i.p*. injected for 3 consecutive days and collected on fourth (200 μl per 30 g body weight), for 3 consecutive days and collected on the fourth.

### RNAscope

*C57BL/6* mice (7 days to 4 months old) were used to verify scRNA-seq candidate gene expression. Dissected mouse mandibles were fixed in 4% paraformaldehyde pH 7.4, overnight, and decalcified in 0.5 M EDTA at +4 °C for 20 days. All samples were embedded in paraffin and sectioned at 7 μm. Tissues were subsequently processed using the RNAscope 2.5 HD Assay-RED detection kit (ACD, 322350, 322360) or RNAscope Multiplex Fluorescent V2 (ACD, 323110) according to the manufacturer’s instructions. Notably, slides were boiled in the target retrieval buffer and incubated in Protease Plus solution at 40 °C for 15 min before probes were incubated at 40 °C for 2 h. The following probes were used: with *Csf1* (315621), *Csf1r* (428191) or *Sp7* (403401). Samples were counterstained with Hematoxylin Gills #2 (20% dilution) for 15 s, followed by 10 s in ammonium hydroxide. Imaging was performed using a Leica DM5000 B and Zeiss AxioScan.Z1.

### Single cell preparation and scRNA-seq

Wildtype *C56Bl6* animals were sacrificed by isoflurane overdose. Mandibles were carefully dissected and under a stereomicroscope and surrounding soft tissue was removed. Using a scalpel and scissors, mandibular bone was gradually removed to obtain separated incisors. Particularly careful handling was performed in the soft area around the most proximal part of the incisor where cervical loops are. Mouse dental pulps with dental epithelium were isolated, cut into small pieces, transferred to a 15 mL Falcon tube with 2.5 mL Collagenase P (3 μ/mL; Sigma-Aldrich, COLLA-RO ROCHE) dissolved in HBSS and incubated for 20–30 min at 37 °C, shaking (120 rpm). During enzymatic digestion, tissue pieces were homogenized 3 times using 1 ml pipet. After incubation, the suspension was finally homogenized using pipet and 10 mL of 2% FBS (ThermoFisher Scientific, 10500064) in HBSS was slowly added. The suspension was centrifuged in a 4 °C precooled centrifuge for 10 min at 300 × *g* resuspended and processed according to the 10x® single cell transcriptomics manufacturer protocol.

### Flow cytometry (FC)

Incisor pulp was extracted in ice-cold PBS and cut into small pieces using fine scissors. For each condition, a total of 6 mice were used. Mandibular and maxillary incisor pulps from two mice were pooled to generate one biological replicate, resulting in three biological replicates per condition. The pulp was then re-suspended in 5 ml of Collagenase D (0.5 μ/ml, Roche, 11088866001) and Dispase II (1.5 μ/ml, Roche, 4942078001). The tissue was allowed to dissociate by incubating the suspension in a cell culture incubator at 37 °C in 5% CO_2_ for 30 min. Following enzymatic digestion, the cell suspension was filtered through a 70 µm Falcon Cell Strainer (Falcon, 352350) and the enzyme reaction quenched using 10 ml of ice-cold PBS. Cells were centrifuged at 300 × *g* for 10 min, and resuspended in 200 μl of FC staining buffer (BioLegend, 420201). 0.10 μg of rat anti-mouse GR1—Alexa Fluor 488 conjugated (108417, BioLegend), and rat anti mouse F4/80—APC conjugated (123116/BioLegend) were added to the cell suspension. Cells were incubated with the antibodies on ice for 30 min. Excess staining buffer was added to quench the reaction, and cells were centrifuged twice as before to remove excess antibody. Following the final centrifugation, cells were resuspended in 500 µl of staining buffer, and compatible fixable viability stain FV780 (BD Bioscience, 565388) added to as a dead cell exclusion marker. Samples were then analyzed on BD FC Aria III fusion machine, and at least 10,000 events were recorded per biological replicate. Data analysis was performed on FlowJo v10 software. Cells were gated based on size using standard SSC-A and FSC-A parameters so that debris is excluded. Following gating of cells, we focused on single cells and excluded doublets using FSC-A and FSC-H parameters (Supplementary Fig. 6). Appropriate gating strategies were then used to select cells positive for the antibodies being used, as deduced from the use of unstained controls (Supplementary Fig. 6).

### X-ray microtomographic imaging (microCT)

To visualize the normal and mutant morphologies in intact mouse specimens, heads were skinned, mounted in plastic tubes with aqueous buffer, and scanned without contrast staining in a SkyScan 1174 microtomography (Bruker). Projection images were obtained over 360° rotation at 0.39° intervals (for interdigitating ray paths from opposite sides of the sample), with 4 s exposure time, 3-frame averaging, and an X-ray source (tungsten) at 50 kVp and 40 W with no beam filter. Whole heads were scanned in two parts, and stitched tomographic sections were reconstructed with the Bruker NRecon software as 16-bit TIFF image stacks with an isotropic voxel size of 12.3 µm. Volume images were visualized and renderings made using Amira 6.4 and VGStudio MAX. Mandibles of adult CD1 mice that underwent macrophage depletion treatment were collected and fixed with 4% PFA overnight. The mandibles were scanned using a Bruker Skyscan1272 micro-CT scanner. After scanning, Microview software programme (GE) was used for visualization and imaging.

### Data pre-processing of 10x Chromium samples

CellRanger- 10× Chromium software was used to perform alignment to GRCh38 human genome or mm10 mouse genome assemblies, filtering, barcode counting and UMI counting. Pre-processing was performed using CellRanger 3.0.2, followed by default CellRanger 3.0.2 filtering of cells. Additionally, a protocol of library preparation used by the facility included spike-in of Jurkat and 32D cells of human and mouse species. Spike-in cells were not used for data processing or analysis and were excluded as *Hbb*^+^ clusters; they are also easily detectable as having low complexity and forming a separate outlier transcriptional cluster. We used CellChat^48^ to estimate the number and patterns of potential intercellular interactions based on cell type annotation and gene expression profiles. We used overexpressed genes with an absolute fold change of at least 0.1 and *p*-value < 0.01 and the default CellChat workflow to prioritize over-expressed pairs of ligands and receptors.

### Comparison of Clodrosome-treated and normal incisors

Clodrosome-treated (2628 cells) and normal (4236 cells) 10× Chromium datasets were processed using PAGODA2 R package using the default procedure with plain batch correction followed by reduction to 25 principal components using 2000 overdispersed genes. Leiden clustering (resolution=1) was performed using a k-nearest neighbor graph constructed from cosine-based cell-cell distance in 25-dimensional PC space. TSNE 2d embedding was calculated using the same distance metric with perplexity=30. To assess genes differentially expressed in odontoblasts and pre-odontoblasts, clusters of odontoblasts and pre-odontoblasts were isolated and only Clodrosome-treated cells were retained. Differential expression was estimated as fold change between cluster-averaged gene expression levels, and *p*-value was estimated using *t*-test for comparison of cluster mean expression levels. To estimate heterogeneity inside Clodrosome-treated macrophages, the first principal component of variability among Clodrosome-treated macrophages, selected from macrophages Leiden clusters, was estimated in 25-dimensional PC space. Scores of the macrophage principal component in non-treated macrophages were estimated as a dot product of the component and cells' PC coordinates in 25d PC space. Biological processes associated with the identified axis of the macrophage variability were estimated for 121 genes with top loadings (>0.05) onto the principal component using the Gostats R package^112^. In the same dataset, we performed differential expression analysis between Clodrosome and control macrophages using the default Seurat FindMarkers() function.

### Statistics and reproducibility

No statistical methods were used to predetermine sample size. Sample sizes (typically *n* = 3–6 animals per group) were selected based on experimental feasibility, sample availability, and consistency with established practices in similar in vivo studies. These group sizes were sufficient to detect reproducible and biologically relevant differences. All experiments were performed with biological replicates, and technical replicates were included to show the consistency of the measurements and technique. The exact number of replicates and statistical test performed for each experiment is provided in the corresponding figure legends. The experiments were not randomized, and the investigators were not blinded to allocation during experiments and outcome assessment. Blinding was not performed in this study. For experiments involving genetically modified animals, group allocation could not be concealed due to visible phenotypic differences. For pharmacological treatments, animals were housed separately according to treatment group, and analyzes were conducted with knowledge of experimental conditions.

### Reporting summary

Further information on research design is available in the Nature Portfolio Reporting Summary linked to this article.

## Supplementary information

Supplementary Information

Reporting Summary

Transparent Peer Review file

## Source data

Source Data File

## Data Availability

The single-cell RNA-seq datasets used in this study were already published and available under accession code GSE146123. Source data are provided with this paper.
