# Supplementary material for: Embryonic and postnatal macrophages are necessary for proper tooth development and homeostasis
Source: Nat Commun. 2026 Jul 17;17:6537. doi: 10.1038/s41467-026-75576-7 (PMC13379590; doi:10.1038/s41467-026-75576-7)
Supplement: Supplementary file 1 — Supplementary Information [file 41467_2026_75576_MOESM1_ESM.pdf]

Supplementary Information

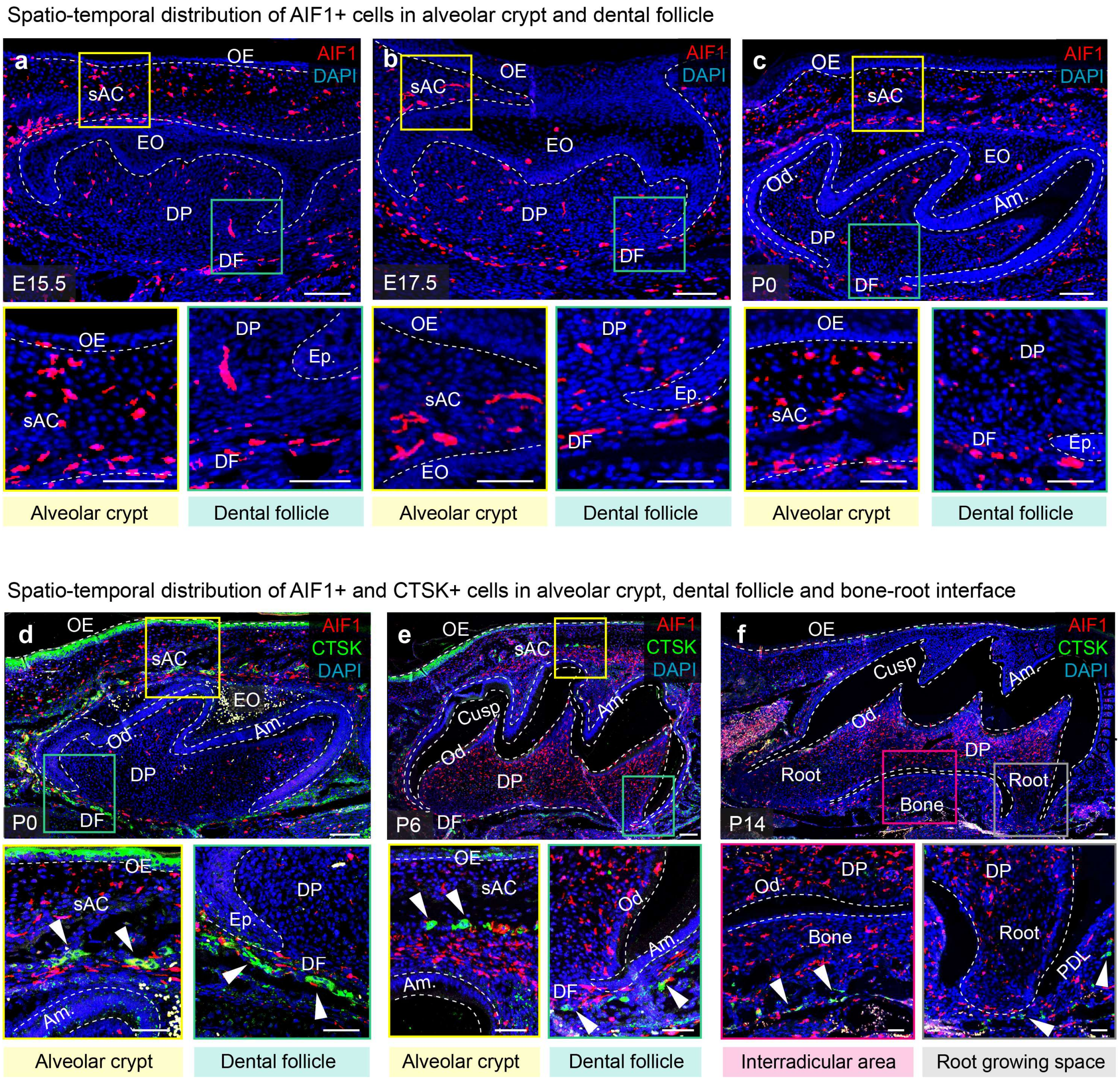

Supplementary Figure 1. Spatio-temporal distribution of AIF1- and CTSK-positive cells during molar development. At pre-/perinatal stages (E15.5, E17.5, P0), macrophages are located not only in the dental pulp but also in the superior wall of the alveolar crypt above the enamel organ/cusps (eruption space), and along the dental follicle **(a-c)**. From postnatal stages (P0, P6, and P14) alveolar bone is being resorbed as indicated by the presence of CTSK-positive cells (white arrowheads) in the superior wall of alveolar crypt (allowing molar eruption), and in the bone around the root growing space from P6 (allowing root elongation) **(d-f)**. Scale bars = 100 µm, insets = 50 µm. Am. = ameloblasts, DF = dental follicle, DP = dental pulp, E = embryonic day, EO = enamel organ, Ep. = epithelium, Od. = odontoblasts, OE = oral epithelium, P = postnatal day, sAC = superior wall of alveolar crypt.

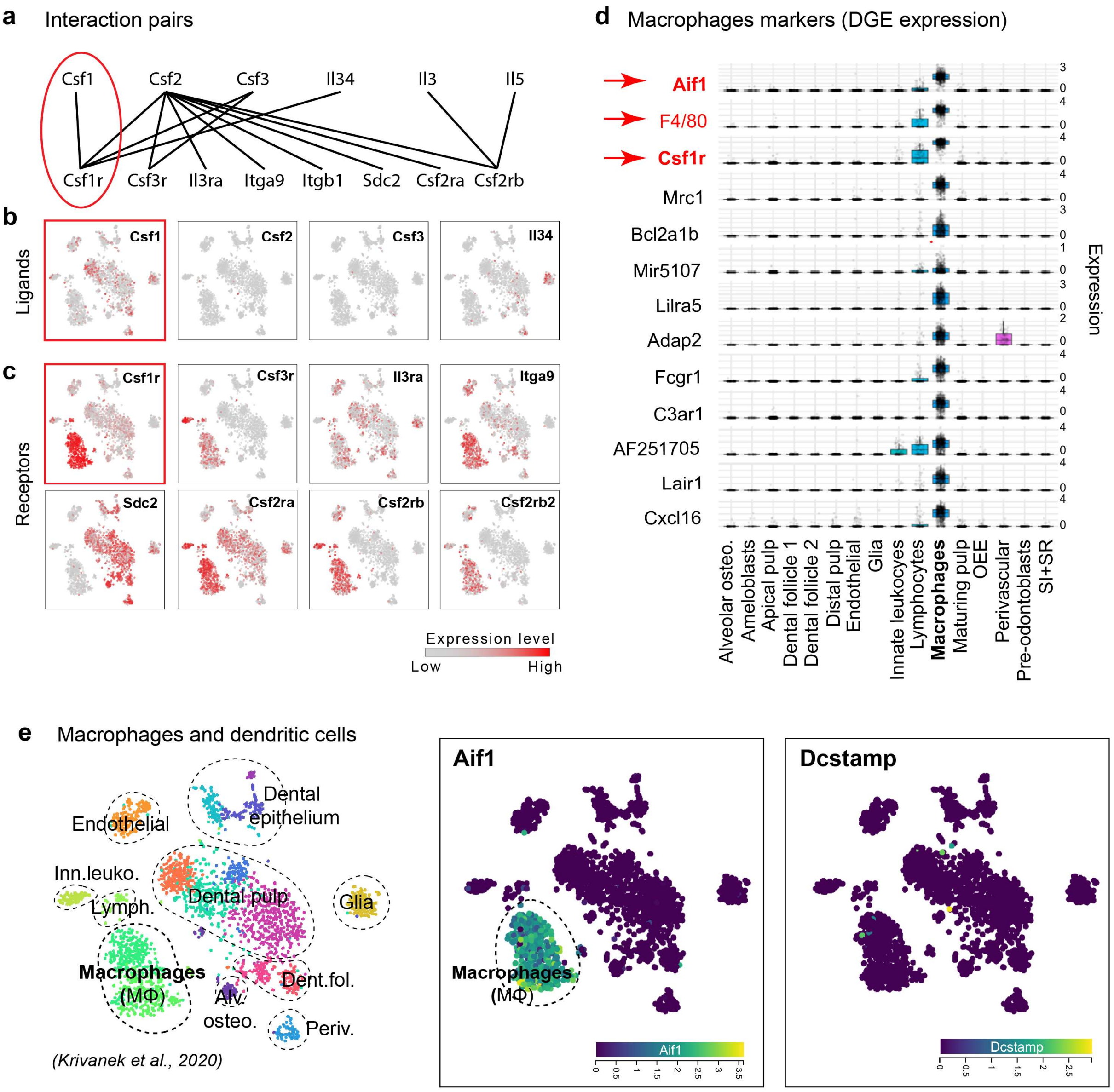

Supplementary Figure 2. Csf1 signaling system is the tooth macrophages Cross-interaction pairs of CSF signaling system **(a)**, heatmap of the expression level in mouse incisor of ligands **(b)** mostly located in the dental pulp cluster and receptors **(c)** localized in the immune system and dental pulp clusters. Highly differentially expressed genes in the macrophage cluster versus rest of cell cluster in continuously-growing incisor **(d)** reveals that *Aif1*, *F4/80* and *Csf1r* markers are co-localized within the same cell type along with other known markers of macrophages. Identification and quantitative expression of monocyte-derived lineage cells in continuously growing incisor **(e)**.

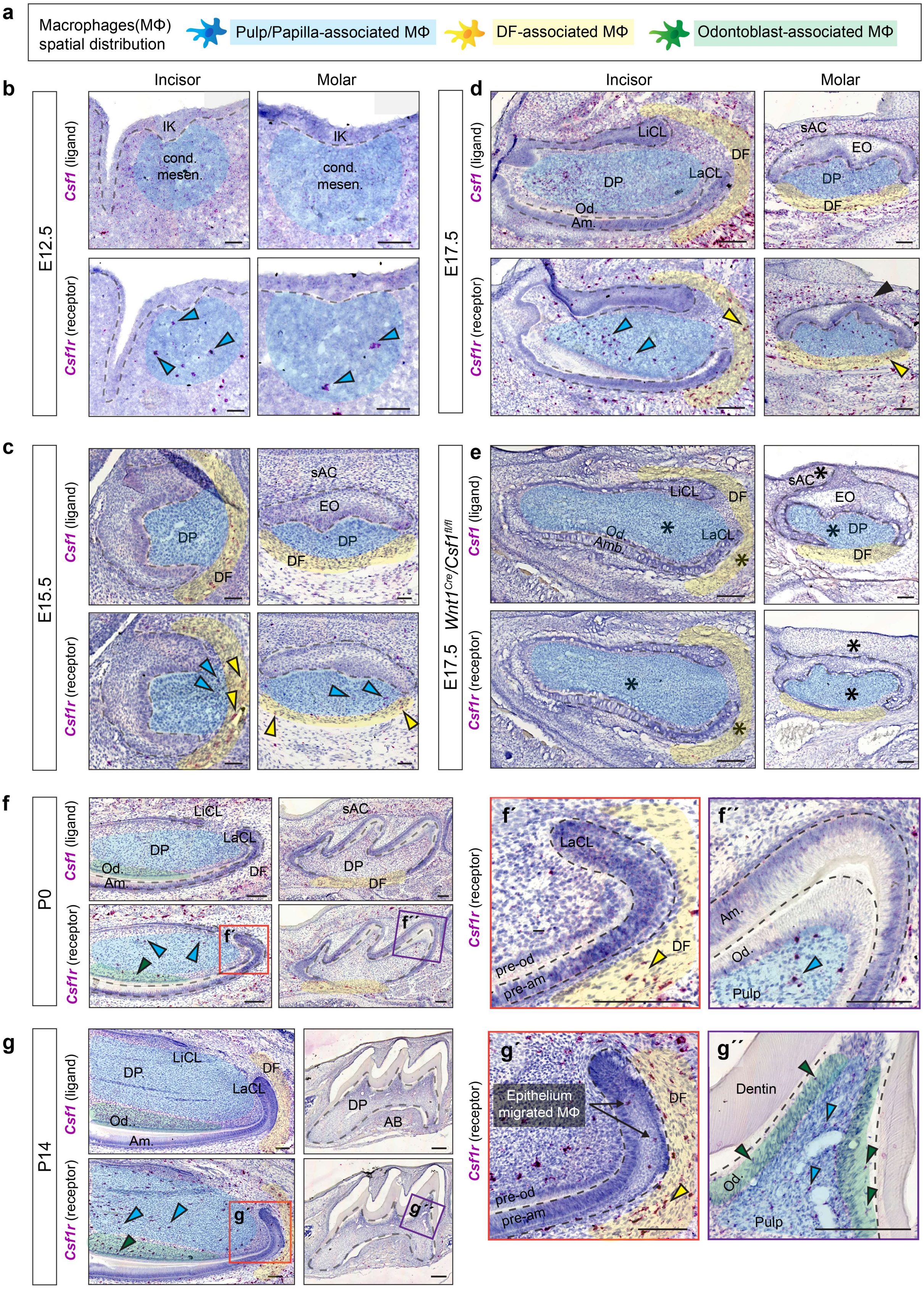

Supplementary Figure 3. Spatio-temporal distribution of *Csf1* ligand and receptor during mouse tooth development. Schematic representation of the different locations and associated cells that macrophages identified during tooth development **(a)**. *In situ* hybridization of *Csf1* and *Csf1r* expression

during the mouse incisor and molar development from early tooth development to postnatal stages shows the dynamics of *Csf1r*+ macrophage colonization of the dental tissue and the *Csf1* ligand spatial distribution **(b-g).** Macrophages populate firstly populate dental papilla/pulp (highlighted in blue and by blue arrowheads) from E12.5 **(b)**, are associated with dental follicle (highlighted in yellow and by yellow arrowheads) from E15.5 **(c)** and increase their presence at E17.5 **(d)**. *Csf1* and *Csf1r* genes are not expressed in $Wnt1^{Cre}/Csf1^{fl/fl}$ mouse teeth (asterisks) **(e)**. At postnatal stages, *Csf1* and *Csf1r* signal close to the odontoblast layer (highlighted in green and by green arrowheads) confirms that macrophages are attracted to this region where they are maintained **(f, g)**. Detection of *Csf1r* at P14 of epithelium-migrated macrophages **(g)**. Scale bars = 100 µm. Am. = ameloblasts, Cond. Mesen. = condensed mesenchyme, DF = dental follicle, EO = enamel organ, IK = initiation knot, DP = dental pulp, LaCL = labial cervical loop, LiCL = lingual cervical loop, Od. = odontoblasts, Mφ= macrophages, pre-od= pre-odontoblasts, pre-am = pre-ameloblasts.

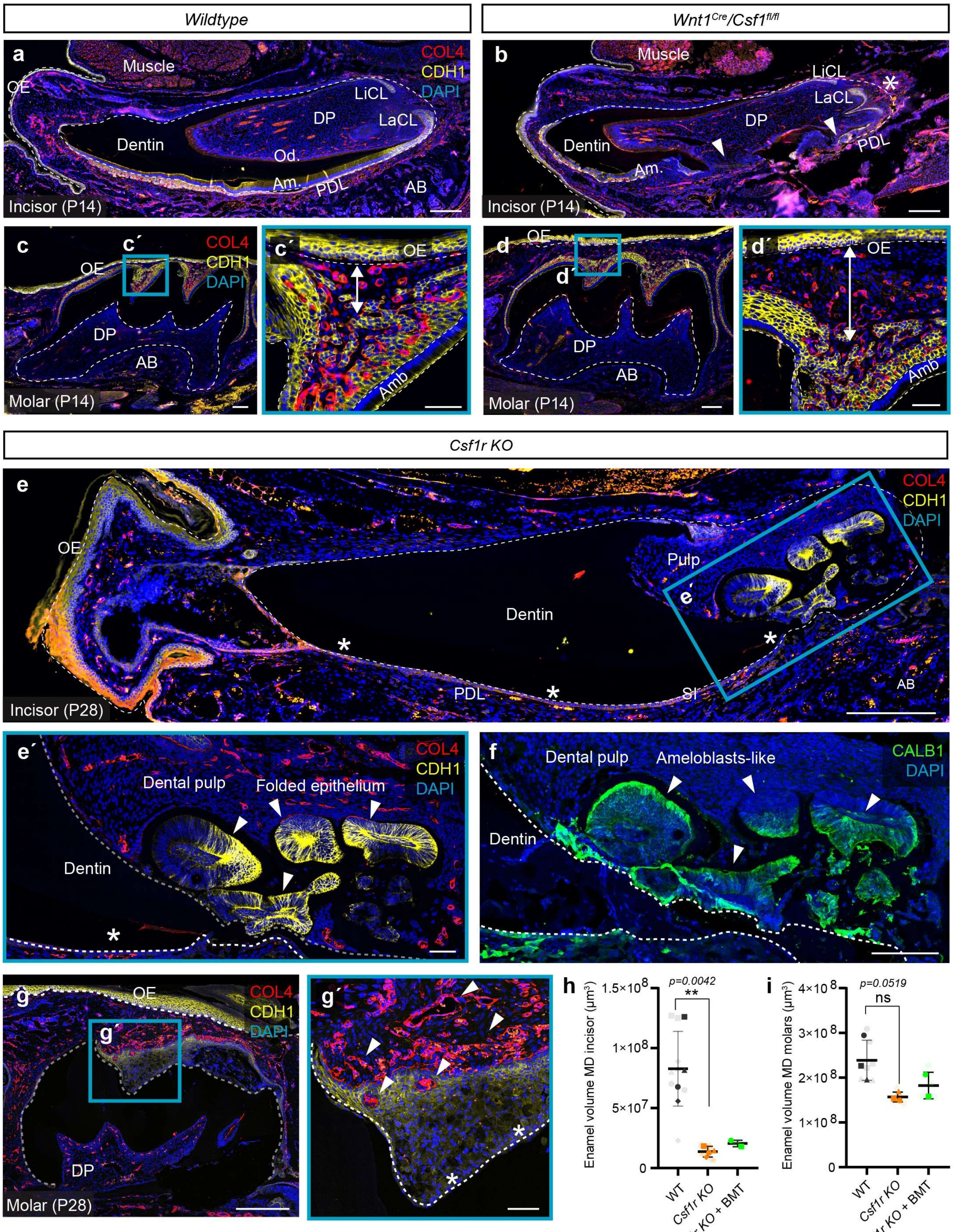

Supplementary Figure 4. Epithelial defects caused by macrophage depletion (*Wnt1^{Cre}/Csf1$^{fl/fl}$* and *Csf1R KO*). Comparison of the epithelial structure and morphology between *Wnt1^{Cre}/Csf1$^{fl/fl}$* with wildtype mice **(a,b)** reveals disruptions in the epithelial layer at various ameloblast stages, resulting in discontinuities of the pulp–periodontal ligament interface (asterisks). Molar eruption delayed in

Wnt1^{Cre}/Csf1$^{fl/fl}$ animals is visible through an increased distance between the enamel organ epithelium and the oral epithelium (white arrow) **(c,d)**. *Csf1R KO* mice present a more severe epithelial phenotype in the continuously growing incisor **(e)**, ameloblast epithelial layer appears condensed and folded towards the apical part of the tooth **(e,e´)** and missing ameloblasts along major portion of the labial side of the incisor (asterisks). Folded ameloblasts/ameloblast-like cells retain CALB1 expression in the mouse incisor **(f)**. The epithelium above the molar cusps is lost, and the eruption space is visibly more vascularized in mutant mice (white arrowheads) **(g,g´)**. The enamel matrix was quantified in control mice, *Csf1R KO,* and after bone marrow transplant rescue **(h,i),** Each symbol represents one animal. The analysis included n = 3 biologically independent animals per group for wildtype and *Csf1r KO* mice (sex distribution: 2 females and 1 male in each group) and n = 2 biologically independent BMT-rescued animals (both females). Data are presented as mean ± SD. Statistical comparisons were performed between wild-type and Csf1r KO groups using a two-sided unpaired Student's *t*-test. Exact *P* values are shown in the graphs; $P < 0.05$, P < 0.01, $P < 0.001$, P < 0.0001. No statistical analysis was performed for the BMT-rescued group because the sample size was below the predefined minimum required for statistical testing (n < 3). Scale bars = 100 µm. AB = alveolar bone, Am, = ameloblasts, DF = dental follicle, EO = enamel organ, IK = initiation knot, DP = dental pulp, LaCL = labial cervical loop, LiCL = lingual cervical loop, Od. = odontoblasts, OE= oral epithelium, PDL = periodontal ligament, pre-od. = pre-odontoblasts, pre-am. = pre-ameloblasts, SI = stratum intermedium.

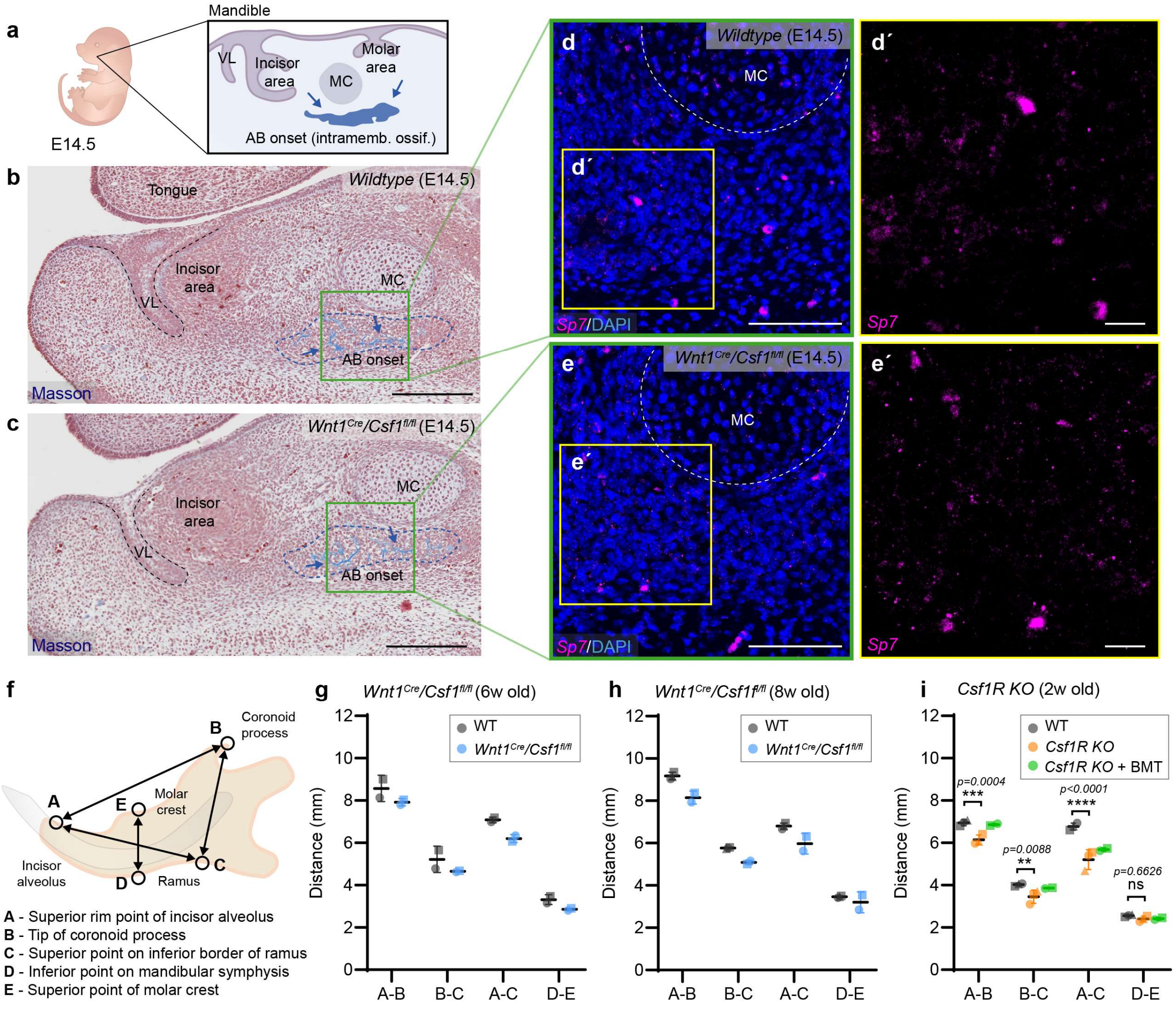

Supplementary Figure 5. Early bone formation and mandibular sizing effects caused by macrophage depletion (*$Wnt1^{Cre}/Csf1^{fl/fl}$* and *Csf1R KO*). Onset of alveolar bone formation characterized by intramembranous ossification at embryonic day 14.5 (E14.5) **(a)**. Collagen-rich osteoid deposition in the developing alveolar bone region between Meckel´s cartilage and the incisor show no major changes in the extent or spatial organization of early bone matrix in $Wnt1^{Cre}/Csf1^{fl/fl}$ compared to controls **(b,c)**. *In situ* hybridization for *Sp7* (Osterix) expression reveals comparable expression in $Wnt1^{Cre}/Csf1^{fl/fl}$ animals, suggesting no alteration in early osteoblast specification **(d,e).** Morphometric analysis using the indicated landmarks **(f)** shows that mandibles from $Wnt1^{Cre}/Csf1^{fl/fl}$ animals present a mild reduction of the jaw size, with a more pronounced decrease in the anteroposterior length than in the vertical height (**g,h**). No statistical analysis was performed for $Wnt1^{Cre}/Csf1^{fl/fl}$ because the sample size was n=2 animals per genotype and age. In contrast, *Csf1R KO* animals display most severe mandibular defects, affecting both length and height parameters (**i**). Statistical comparisons were performed only between wildtype and *Csf1r KO* animals using a two-sided unpaired Student's *t*-test. Exact *P* values are shown in the graphs; $P < 0.05$, $P < 0.01$, $P < 0.001$, $P < 0.0001$. Scale bars: (b-e) = 100 µm; (d´,e´) = 20 µm. AB = alveolar bone, MC = Meckel´s cartilage, VL = vestibular lamina.

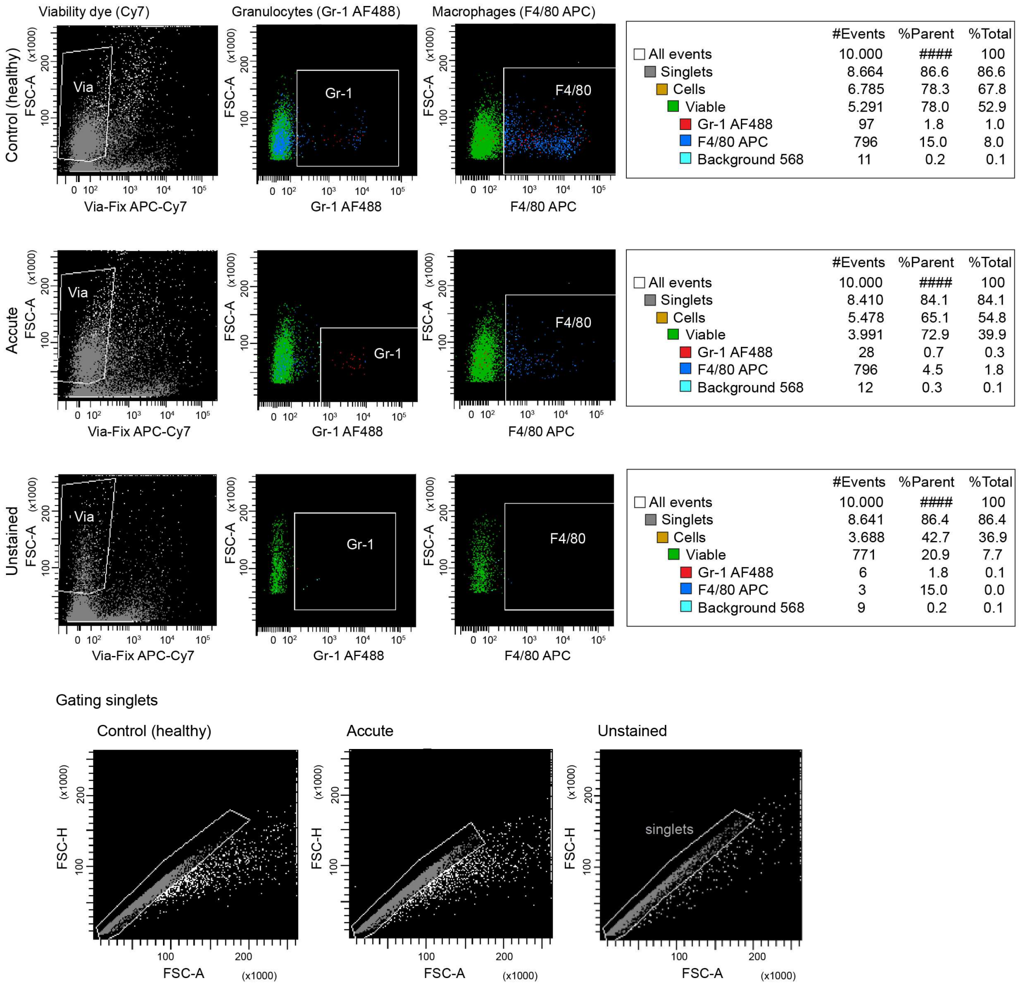

Supplementary Figure 6. Representative gating strategies followed for antibody stainings (Gr-1 and F4/80) and doublets exclusion.

Supplementary Table 1. Summary of observed incisor phenotypes in genetic models

Mandibular incisors Genotype (age)	Shorter incisor	Folded incisor	Erupted	Reduced pulp chamber	Bone-tooth fused	Enamel present
WT *Wnt1*^{Cre}/*Csf1*$^{fl/fl}$ (6 w)	0/2	0/2	2/2	0/2	0/2	2/2
WT *Wnt1*^{Cre}/*Csf1*$^{fl/fl}$(8w)	0/3	0/3	3/3	0/3	0/3	3/3
KO *Wnt1*^{Cre}/*Csf1*$^{fl/fl}$ (6w)	2/2	1/2	2/2	1/2	2/2	1/2
KO *Wnt1*^{Cre}/*Csf1*$^{fl/fl}$ (8w)	2/2	2/2	2/2	2/2	2/2	2/2
Csf1r$^{+/+}$ (2w)	0/6	0/6	6/6	0/6	0/6	6/6
Csf1r$^{+/+}$ + BMT (2w)	0/5	0/5	5/5	0/5	0/5	5/5
Csf1r$^{-/-}$ *KO* (2w)	5/5	5/5	0/5	0/5	5/5	0/5
Csf1r$^{-/-}$ *KO* + BMT (2w)	4/4	4/4	3/4	1/4	4/4	3/4
Maxillary incisors Genotype (age)	Shorter incisor	Folded incisor	Erupted	Reduced pulp chamber	Bone-tooth fused	Enamel present
WT *Wnt1*^{Cre}/*Csf1*$^{fl/fl}$ (6 w)	0/2	0/2	2/2	0/2	0/2	2/2
WT *Wnt1*^{Cre}/*Csf1*$^{fl/fl}$(8w)	0/3	0/3	3/3	0/3	0/3	3/3
KO *Wnt1*^{Cre}/*Csf1*$^{fl/fl}$ (6w)	2/2	1/2	2/2	1/2	1/2	1/2
KO *Wnt1*^{Cre}/*Csf1*$^{fl/fl}$ (8w)	2/2	2/2	2/2	2/2	1/2	2/2
Csf1r$^{+/+}$ (2w)	0/6	0/6	6/6	0/6	0/6	6/6
Csf1r$^{+/+}$ + BMT (2w)	0/5	0/5	5/5	0/5	0/5	5/5
Csf1r$^{-/-}$ *KO* (2w)	5/5	5/5	5/5	0/5	5/5	0/5
Csf1r$^{-/-}$ *KO* + BMT (2w)	4/4	4/4	3/4	2/4	4/4	1/4

Supplementary Table 2. Summary of observed molar phenotypes in genetic models

Mandibular molars Genotype (age)	Presence of 2 roots	Normal appearance of roots	Normal patterning of cusps	Supernumerary teeth	Erupted
WT *Wnt1*^{Cre}/*Csf1*$^{fl/fl}$ (6 w)	2/2	2/2	2/2	0/2	2/2
WT *Wnt1*^{Cre}/*Csf1*$^{fl/fl}$(8w)	3/3	3/3	3/3	0/3	3/3
KO *Wnt1*^{Cre}/*Csf1*$^{fl/fl}$ (6w)	2/2	0/2	0/2	1/2	2/2
KO *Wnt1*^{Cre}/*Csf1*$^{fl/fl}$ (8w)	2/2	0/2	0/2	0/2	2/2
Csf1r$^{+/+}$ (2w)	6/6	6/6	6/6	0/6	6/6
Csf1r$^{+/+}$ + BMT (2w)	5/5	5/5	5/5	0/5	5/5
Csf1r$^{-/-}$ *KO* (2w)	0/5	-	0/5	0/5	0/5
Csf1r$^{-/-}$ *KO* + BMT (2w)	1/4	-	0/4	0/4	1/4
Maxillary molars Genotype (age)	Presence of 3 roots	Normal appearance of roots	Normal patterning of cusps	Supernumerary teeth	Erupted
WT *Wnt1*^{Cre}/*Csf1*$^{fl/fl}$ (6 w)	2/2	2/2	2/2	0/2	2/2
WT *Wnt1*^{Cre}/*Csf1*$^{fl/fl}$(8w)	3/3	3/3	3/3	0/3	3/3
KO *Wnt1*^{Cre}/*Csf1*$^{fl/fl}$ (6w)	1/2	0/2	0/2	0/2	2/2
KO *Wnt1*^{Cre}/*Csf1*$^{fl/fl}$ (8w)	1/2	0/2	1/2	0/2	2/2
Csf1r$^{+/+}$ (2w)	6/6	6/6	6/6	0/6	6/6
Csf1r$^{+/+}$ + BMT (2w)	5/5	5/5	5/5	0/5	5/5
Csf1r$^{-/-}$ *KO* (2w)	0/5	-	0/5	0/5	0/5
Csf1r$^{-/-}$ *KO* + BMT (2w)	1/4	-	1/4	0/4	0/4
