# Supplementary material for: Embryonic and postnatal macrophages are necessary for proper tooth development and homeostasis
Source: Nat Commun. 2026 Jul 17;17:6537. doi: 10.1038/s41467-026-75576-7 (PMC13379590; doi:10.1038/s41467-026-75576-7)
Supplement: Supplementary file 2 — Reporting Summary [file 41467_2026_75576_MOESM2_ESM.pdf]

nature portfolio

Corresponding author(s): Jan Krivanek
Igor Adameyko

Last updated by author(s): Jun 8, 2026

Reporting Summary

Nature Portfolio wishes to improve the reproducibility of the work that we publish. This form provides structure for consistency and transparency in reporting. For further information on Nature Portfolio policies, see our Editorial Policies and the Editorial Policy Checklist.

Statistics

For all statistical analyses, confirm that the following items are present in the figure legend, table legend, main text, or Methods section.

n/a	Confirmed	
☐	☒	The exact sample size (*n*) for each experimental group/condition, given as a discrete number and unit of measurement
☐	☒	A statement on whether measurements were taken from distinct samples or whether the same sample was measured repeatedly
☐	☒	The statistical test(s) used AND whether they are one- or two-sided<br>*Only common tests should be described solely by name; describe more complex techniques in the Methods section.*
☒	☐	A description of all covariates tested
☒	☐	A description of any assumptions or corrections, such as tests of normality and adjustment for multiple comparisons
☐	☒	A full description of the statistical parameters including central tendency (e.g. means) or other basic estimates (e.g. regression coefficient) AND variation (e.g. standard deviation) or associated estimates of uncertainty (e.g. confidence intervals)
☐	☒	For null hypothesis testing, the test statistic (e.g. *F*, *t*, *r*) with confidence intervals, effect sizes, degrees of freedom and *P* value noted<br>*Give P values as exact values whenever suitable.*
☒	☐	For Bayesian analysis, information on the choice of priors and Markov chain Monte Carlo settings
☒	☐	For hierarchical and complex designs, identification of the appropriate level for tests and full reporting of outcomes
☒	☐	Estimates of effect sizes (e.g. Cohen's *d*, Pearson's *r*), indicating how they were calculated

Our web collection on statistics for biologists contains articles on many of the points above.

Software and code

Policy information about availability of computer code

Data collection	Data collection varied according to the experimental methodology:<br>- Immunohistochemistry (IHC) and fluorescence imaging: Stained sections were imaged using an Zeiss AxioScan 7 and Zeiss LSM880 with acquisition performed using ZEN Blue/ZEN Black. Image exports was performed in ZEN3.4 or Imaris Free Viewer.<br>- Micro-computed tomography (microCT):S amples were scanned using SkyScan 1174 and SkyScan 1272 (Bruker) systems.<br>- Flow cytometry:Stained single-cell suspensions were acquired using a BD FACS Aria III.<br>- Histology and RNA in situ hybridization:Conventional histological stainings (e.g., Masson's Trichrome, TRAP) and RNA in situ hybridization were performed using the RNAscope assay. Imaging was performed using brightfield and slide-scanning microscopy systems (Axioscan 7).<br>- Single-cell transcriptomics:No new sequencing data were generated. Publicly available single-cell RNA-seq datasets (GEO accession: GSE146123) originally published in Krivanek et al. 2020 were used. Data collection and sequencing procedures are described in the original publication.<br>- Cytokine array:Protein lysates from mouse tissue were analysed using the Proteome Profiler Mouse Cytokine Array Kit following the manufacturer's instructions.
Data analysis	Data analysis was performed using the following software tools, depending on the methodology:<br>- Image analysis and cell quantification: Manual quantification of AIF1+, CTSK+, and double-positive cells, and distances of eruption or root length in 2D sections were performed using ZEN3.4 Blue. Meanwhile for quantifications and analysis for area-based it was used eiter QuPath0.5.0 or ImageJ.<br>- Morphometric and microCT analysis:Three-dimensional reconstruction and morphometric analyses of microCT data were performed using VGStudio MAX. Additional visualization and reconstruction were conducted using Amira.<br>- Flow cytometry analysis:Flow cytometry data were analysed using FlowJo v10, applying gating strategies to exclude debris, doublets, and dead cells.

- Single-cell RNA-seq analysis:Pre-processing (alignment, filtering, and UMI counting) was performed using Cell Ranger. Downstream analyses were conducted using PAGODA2, Seurat, and CellChat.Dimensionality reduction (PCA, t-SNE), clustering (Leiden algorithm), and differential gene expression analyses were performed using default or specified parameters as described in the Methods.Statistical and bioinformatic analysis: Gene ontology and pathway enrichment analyses were performed using GOstats.

For manuscripts utilizing custom algorithms or software that are central to the research but not yet described in published literature, software must be made available to editors and reviewers. We strongly encourage code deposition in a community repository (e.g. GitHub). See the Nature Portfolio guidelines for submitting code & software for further information.

Data

Policy information about availability of data

All manuscripts must include a data availability statement. This statement should provide the following information, where applicable:

- Accession codes, unique identifiers, or web links for publicly available datasets
- A description of any restrictions on data availability
- For clinical datasets or third party data, please ensure that the statement adheres to our policy

All single-cell RNA sequencing datasets analysed in this study are publicly available in Gene Expression Omnibus under accession code GSE146123, originally reported in Krivanek et al. 2020.

Research involving human participants, their data, or biological material

Policy information about studies with human participants or human data. See also policy information about sex, gender (identity/presentation), and sexual orientation and race, ethnicity and racism.

Reporting on sex and gender	Not applicable, as this study did not involve human participants
Reporting on race, ethnicity, or other socially relevant groupings	Not applicable, as this study did not involve human participants
Population characteristics	Not applicable, as this study did not involve human participants
Recruitment	Not applicable, as this study did not involve human participants
Ethics oversight	Not applicable, as this study did not involve human participants

Note that full information on the approval of the study protocol must also be provided in the manuscript.

Field-specific reporting

Please select the one below that is the best fit for your research. If you are not sure, read the appropriate sections before making your selection.

☒ Life sciences ☐ Behavioural & social sciences ☐ Ecological, evolutionary & environmental sciences

For a reference copy of the document with all sections, see nature.com/documents/nr-reporting-summary-flat.pdf

Life sciences study design

All studies must disclose on these points even when the disclosure is negative.

Sample size	Sample sizes (typically n = 3–6 animals per group) were selected based on experimental feasibility, sample availability, and consistency with established practices in similar in vivo studies. These group sizes were sufficient to detect reproducible and biologically relevant differences. Exact sample sizes for each experiment are reported in the corresponding figure legends.
Data exclusions	No data was excluded
Replication	All experiments were performed with biological replicates and technical replicates were included to show the consistency of the measurements and technique. The exact number of replicates for each experiment is provided in the corresponding figure legends. Representative experiments shown are consistent with results obtained across replicates
Randomization	No randomization was performed. Animals were allocated to experimental groups based on genotype or treatment conditions.
Blinding	Blinding was not performed in this study. For experiments involving genetically modified animals, group allocation could not be concealed due to visible phenotypic differences. For pharmacological treatments, animals were housed separately according to treatment group, and analyses (e.g., flow cytometry and immunofluorescence-based quantifications) were conducted with knowledge of experimental conditions.

Reporting for specific materials, systems and methods

We require information from authors about some types of materials, experimental systems and methods used in many studies. Here, indicate whether each material, system or method listed is relevant to your study. If you are not sure if a list item applies to your research, read the appropriate section before selecting a response.

Materials & experimental systems

n/a	Involved in the study
☐	☒ Antibodies
☒	☐ Eukaryotic cell lines
☒	☐ Palaeontology and archaeology
☐	☒ Animals and other organisms
☒	☐ Clinical data
☒	☐ Dual use research of concern
☒	☐ Plants

Methods

n/a	Involved in the study
☒	☐ ChIP-seq
☐	☒ Flow cytometry
☒	☐ MRI-based neuroimaging

Antibodies

Antibodies used	The following primary antibodies were used:<br><br>Immunohistochemistry (IHC):AIF1 (Novus, NB100-1028; 1:500), COL4 (AbD Serotec, 2150-1470; 1:500), CALB1 (Swant, CB-38a; 1:200), CDH1 (Bio-Techne, AF748; 1:500), CTSK (ProteinTech, 11239-1-AP; 1:300), F4/80 (Abcam, ab6640; 1:200), GFP (Acris, R1091P; 1:200), MKI67 (Zytomed, RBK027-05; 1:200).Fluorophore-conjugated secondary antibodies (Alexa Fluor series, Invitrogen) were used at a dilution of 1:1000.<br><br>Flow cytometry: Rat anti-mouse GR1 – Alexa Fluor 488 (BioLegend, 108417; 0.10 µg per sample) and rat anti-mouse F4/80 – APC (BioLegend, 123116; 0.10 µg per sample).
Validation	All antibodies used in this study were selected based on prior validation for use in mouse tissues, as reported by the manufacturers and in the literature. Specificity was further supported by staining patterns consistent with known cellular localization and marker expression.

Animals and other research organisms

Policy information about studies involving animals; ARRIVE guidelines recommended for reporting animal research, and Sex and Gender in Research

Laboratory animals	Animal experiments were approved either by the Ethik-Kommission der MedUni Wien zur Beratung und Begutachtung von Forschungsprojekten am Tier in Austria, Ethical Committee on Animal Experiments (Stockholm North Committee) in Sweden, Ministry of Education, Youth and Sports, Czech Republic (MSMT-6379/2022-4) or IACUC panel of Children's Hospital of Philadelphia. Clodrosome treatments were conducted at CCRB, King's College London under Home Office (Project license number PPL70/7866). Animal experiments were done in accordance with institutional animal care and ethical committees and French and European guidelines for animal care under approval APAFIS#49928-2024061911572138. All mice were kept under SPF conditions. Experiments were performed according to international and local regulations. Mice were housed in 12/12 light/dark cycle, at temperature ranging from 18 to 23 °C and 40–60% humidity. Food and water were provided to the animals ad libitum. Genetically modified or wildtype animals used in this study were C57BL/6 genetic background. Mice used for all experiments were sacrificed by an isoflurane (Baxter KDG9623) overdose.
Wild animals	Not wild animals were involved in the study.
Reporting on sex	Experiments were conducted using both male and female mice. Subsequent analyses were performed without stratification by sex, and results are reported collectively
Field-collected samples	Not field-collected samples were used in the study.
Ethics oversight	All animal experiments were conducted in accordance with institutional and national guidelines for animal welfare and were approved by the respective ethics committees listed above.Animals were housed under specific pathogen-free conditions with a 12-hour light/dark cycle, at a temperature of 18–23 °C and 40–60% humidity, with food and water provided ad libitum.Euthanasia was performed by isoflurane overdose.

Note that full information on the approval of the study protocol must also be provided in the manuscript.

Plants

Seed stocks | Not applicable

Novel plant genotypes | Not applicable

Authentication | Not applicable

Flow Cytometry

Plots

Confirm that:

☒ The axis labels state the marker and fluorochrome used (e.g. CD4-FITC).

☒ The axis scales are clearly visible. Include numbers along axes only for bottom left plot of group (a 'group' is an analysis of identical markers).

☒ All plots are contour plots with outliers or pseudocolor plots.

☒ A numerical value for number of cells or percentage (with statistics) is provided.

Methodology

Sample preparation | Incisor dental pulp was isolated from control and clodrosome-treated mice and processed into single-cell suspensions as described in the Methods section. For each condition, six mice were used, with mandibular and maxillary incisor pulps from two mice pooled to generate one biological replicate (n = 3 replicates per condition). Tissues were mechanically dissociated, enzymatically digested, filtered, and stained with fluorophore-conjugated antibodies against Gr-1 (Alexa Fluor 488) and F4/80 (APC). A fixable viability dye (FV780) was used to exclude dead cells.

Instrument | BD FC Aria III

Software | FlowJo v10

Cell population abundance | Cell populations were quantified as percentages of parent populations following sequential gating. Gr-1+ and F4/80+ populations were calculated as a percentage of the total population. At least 10,000 events were recorded per biological replicate, and gating strategies were applied across all samples.

Gating strategy | Cells were initially gated based on forward scatter (FSC-A) and side scatter (SSC-A) to exclude debris. Doublets were excluded using FSC-A versus FSC-H. Viable cells were identified by exclusion of FV780-positive events. Gates for Gr-1 (Alexa Fluor 488) and F4/80 (APC) positivity were defined based on unstained controls and applied consistently across all samples. The full gating strategy is shown in Supplementary Figure 6.

☒ Tick this box to confirm that a figure exemplifying the gating strategy is provided in the Supplementary Information.
