# Supplementary material for: Embryonic and postnatal macrophages are necessary for proper tooth development and homeostasis
Source: Nat Commun. 2026 Jul 17;17:6537. doi: 10.1038/s41467-026-75576-7 (PMC13379590; doi:10.1038/s41467-026-75576-7)
Supplement: Supplementary file 3 — Transparent Peer Review file [file 41467_2026_75576_MOESM3_ESM.pdf]

nature portfolio

Peer Review File

Embryonic and postnatal macrophages are necessary for proper tooth development and homeostasis

Corresponding Author: Dr Jan Krivanek

This file contains all reviewer reports in order by version, followed by all author rebuttals in order by version.

Version 0:

Reviewer comments:

Reviewer #1

(Remarks to the Author)
December 13, 2024
Manuscript: NCOMMS – 24 - 74495 Title: Embryonic and postnatal macrophages are essential for dental morphogenesis and self-renewal Reviewers' comments:
As evidenced in the literature, the development of dentition involves a complex interplay of transcription factors, growth factors and extracellular matrix molecules whose coordinated activities control the position, shape and size of teeth. Signaling pathways also control the formation of specialized matrices and the eruptive pathways that allow for an encased tooth to erupt from within its bony crypt into the oral cavity. In this manuscript the authors draw attention to a relatively unexplored role for macrophages during early to late phases of tooth development Using multi-pronged analyses, they propose that resident macrophages a sub-population of macrophages that are non-circulating in nature, do play key roles in tooth morphogenesis, homeostasis and regeneration. The well characterized murine molar and (continuously erupting) incisor are the models in these studies that utilize scRNA-seq and FACs analyses, genetic ablation using traditional and conditional approaches as well as pharmacological interventions aimed at depleting macrophages in vivo. The authors conclude that long-lived or resident macrophages are present in both epithelial and mesenchymal compartments of tooth organs and are likely to also modulate homeostasis within stem cell niches at the apical end of growing incisors. They further postulate a non-immune role for these macrophages during the process of tooth eruption.
Areas of major concern:
1.
As written, the manuscript suffers from a (perceived) lack of understanding of the biological systems that drive the formation of teeth and their subsequent eruption. Such an in-depth understanding is needed for a more meaningful interpretation of the results and must lead to an improved quality of the data sets presented.
2.
Taken together, the distinction between tissue-resident macrophages and those derived from circulation (monocytes) is not clearly established in these studies. Moreover, the results fall short in demonstrating that these cells serve non-immune functions.
3.
While the study focuses on CSF-1 and its receptor with the intent of unraveling their roles in modulating the activation, migration and phagocytosis activities of macrophages, such a delineation of CSF-1 stage-specific functions is not provided.
4.
It is unclear why these studies focus on CSF-1 alone when it is well known that multiple cytokines such as the interleukins, TGF-beta, TNF, PTH and RANKl also influence osteoclastogenesis, bone resorption and bone remodeling that are essential in late phase of tooth development during root elongation and tooth eruption. Furthermore, the question of how resident macrophages interact with T lymphocytes and dendritic cells that are present within
2
dental pulp and the periodontal ligament
and that also play an important role in immunosurveillance and homeostasis. scRNA-seq methods should make it possible to include a more holistic approach to understanding the micro-environment that dictates the formation, function and fate of resident macrophages throughout the life cycle of a tooth.
5.
As specified below, the authors narrow their interpretation to developing molar and incisor
organs as well as the effects of genetic and pharmacological alteration on pre-eruptive and eruptive phases. A major gap in

analyses relates to the effects of CSF-1/r levels on the state of resident macrophages within alveolar bone, both at the base of growing roots and the bone that overlies a fully formed crown. Such an oversight leads to the conclusion that the biology of root formation/elongation as well as tooth eruption is not well leveraged or explored.

6.

Authors fail to provide insight into their findings of what roles resident or circulating macrophages may play in dental epithelium and the enamel organ, for example, the stellate reticulum and cervical loop area. While the phenotypic changes in dentition are striking, the manuscript can clearly benefit from a more thoughtful interpretation of these descriptive findings.

7.

Figure panels should be attended to, in terms of labeling all relevant structures, correct cross-referencing in the text and alignment with the description provided in the legends. The latter will benefit from a more detailed explanation of what symbols like arrowheads and arrows signify.

Specific suggestions (By Section) :

Title: Should be revised to more accurately reflect the data and conclusions of these studies.

8.

For example, embryonic macrophages can only be described as essential in tooth development if there is an arrest in morphogenesis as seen in the failure to progress from the bud to the cap stage.

9.

The term ‘tooth’ is more universally accepted when compared to ‘dental’.

10.

Perhaps the authors will consider using ‘development’ rather than ‘morphogenesis’ as the former is more inclusive of enamel and dentin formation while morphogenesis typically refers to developmental stages that precede matrix deposition.

11.

Unclear as to what the term ‘self-renewal’ refers to in the title. Is this a reference to data on the effect of the lack of CSF-1/r on incisal growth or the finding of a supernumerary molar? Perhaps the term ‘homeostasis’ may be preferred.

Abstract: Modifications needed based on how the manuscript is shaped after revision and should also accurately reflect the limitations of these studies.

12.

Mention of the use of mouse models of tooth development and eruption is needed as ‘bone marrow transplantation in ‘healthy adults’ can be interpreted as for humans, at first read.

13.

Specifying that the genetic disruption models were used to study macrophage activation is not accurate as tracing studies were not performed.

14.

The concluding statement that these data reveal a non-immune role of dental macrophages is inaccurate based on the present data sets. Clearly, the rise in AIF-1+ cells in pre-eruptive and eruptive phases may suggest a role of circulating monocytes/macrophages and the likely formation of osteoclasts in bone resorption, which implies a role within the inflammatory cytokine pathways.

Introduction: Revise to include literature that will better orient the readership to the role of macrophages in tooth development, eruption and homeostasis

15.

Scaling back the background on macrophage biology by citing recent review/s will allow for the addition of other relevant background.

16.

A brief overview of the presence of other inflammatory cells in a developing and adult dental pulp as well as of macrophages that are also present in the periodontal ligament will be useful to include.

17.

Also important to orient the reader better to the theories of tooth eruption where positive pressure of an elongating root creates osteoclastic activation in the superior wall of the alveolar crypt and the eruption pathway. Classic literature citations should be included along with excellent reviews by Gary Wise along with his work on CSF-1 and tooth eruption.

18.

Information on what is known about dendritic cells that also play a role in immunosurveillance and antigen-presentation during pulpal inflammation is important to conclude.

19.

Tooth morphology should be accurately referred to. For example, the hard outer shell of a tooth is not composed of dentin and enamel (2nd para). It is the anatomical crown that is covered by enamel while dentin that is akin to bone forms the bulk of a mature tooth (crown ad root).

20.

Briefly explain the mechanism by which clodronate liposomes deplete macrophages as it can provide essential background for readers unfamiliar with it.

21.

The concluding paragraph is too speculative and should be revised to accurately reflect the results of these studies.

Results and Figures: This section draws the most criticism and needs a major revision. Key concerns are highlighted as follows.

22.

In the first series, the temporo-spatial profiles of macrophage distribution in molar and incisor organs is described using the marker of phagocytotic macrophages, AIF-1/IBA1. Details of this cytoplasmic protein that is expressed as important for macrophage function will be useful for the unfamiliar reader. It is also unclear whether AIF-1 is expressed by macrophages

that are recruited as part of an inflammatory/immune response.
23.
Figure 1 panels need to be more thoroughly labeled and should capture the superior wall of the alveolar crypt, in low and high magnification views. . This is a critical areas as it is expected that circulating monocytes/macrophages will dominate along with osteoclasts whose resorptive activities become evident during the pre-eruptive phases (onset at PN Day 8 for molars and incisors).
24.
The presence of macrophages in the dental follicle and gingival mucosa seem to be ignored as is the rise in cell density that is visible within alveolar bone within the interradicular area of molar organs.
25.
Page 5, line 18 - How did they identify which are Csf1 ligand and Csf1r receptor that are shown in supplementary Fig 1d?
26.
In result, Page 4, line 33 and also in Page 5, line 3-7, Fig 1jand k is mentioned but these figures are not labeled or referred to in the legend.
27.
The specificity of immunostaining is not convincing as seen in Figure 1i where background staining of odontoblastic processes and (pre) dentin is visible.
28.
The area labeled in panel 1j and described in the text (para 3 of Results) is the collapsed intermediary layers of the enamel/dental organ and should be identified as such. It is curious that the morphology of the immunostained cells so closely resembles that of dendritic cells.
29.
From this panel it is hard to delineate resident macrophages from those that are immunogenic and derived from circulating monocytes.
30.
Figure 2a and 2b should identify the remaining cell clusters as it will be useful to know how the environment that macrophages are exposed to in the presence of Csf1 and also how its absence affects other cell populations.
31.
The developmental stage of tissues used for scRNA-seq should be mentioned in the legend for panels 2a and 2b as well as for 2c and 2d.
32.
As for Figure 1, it is essential for photomicrographic views to cover the superior, inferior and lateral walls of the alveolar crypt.
33.
Figure 2 panels should be labeled accurately with developmental stages clearly marked.
34.
Page 6 line 7: the authors discuss developing molars, but the Figure refers to incisors. The stem cell niche referred to in the figure is only present in developing incisors not molars.
5
35.
Page 6 line 7-8, it should be Fig 2m also lines 10-15 are describing Fig 2 l and Fig 2m but is written as Fig. 2k
36.
Page 6, line 7 and Figure 2j,k – They mentioned that they observed developmental malformations in the developing molars (Fig. 2j, k). Maybe they meant incisors?
37.
Page 5 line 24-25 is confusing as the authors suddenly start talking about Fig. 3 while describing Figure 2.
38.
The presence of a supernumerary tooth placed mesial to the first mandibular molar is intriguing. How often was this observed? What were the total number of samples retrieved for analysis. Was this ever observed in the maxilla? If not, what hypothesis can be framed to explain regional differences in phenotype?
39.
Figure 3 describes the effects of depleting Csf1r and the subsequent changes in molar and incisor organs. These appear strikingly similar to that seen with the conditional depletion of the Csf1 ligand. How can differences in the level of the ligand and receptor for Csf1 be reconciled?
40.
Again, developmental stages are not specified in Figure 3 panels
41.
Page 6, line 27 should be Fig 3g instead of Fig 3i and Fig 3j
42.
With the BMT rescue experiments why are regional differences noted? Specific reference to earlier work of Wise et al., that documented how local administration of Csf1 corrected the failure to erupt should be included in the discussion
43.
Figures 4 and 5 describe results of a pharmacological intervention using a form of bisphosphonate drug to reduce/deplete macrophages, presumably the resident population of cells. It is unclear how the rationale for the delivery regimen was derived. Several details related to the exact dosage are also missing.
44.
Page 8 lines 3-4 should be Fig 4e instead of Fig 4c
45.
Page 8, line 3 – Which treatment and which result depicts that physiological growth and tissue morphology were completely

restored? In Fig 4, there is no mention about panel c in the figure legend.
46.
"Importantly, after the treatment was stopped, the physiological growth and tissue morphology were completely restored (Fig. 4c)". It is not clear which image shows the restored morphology. Also please mention how long after treatment stopped, the recovery was observed and whether this timeline is consistent across different specimens.
6
47.
It is unclear why general macrophage markers – F4/80 and GRI were used instead of AIF1. Conversely, why were these markers that characterize all cells in the monocyte/macrophage lineage not used in studies described in Figures 1, 2 and 3.
48.
Was a power analysis performed to determine the number of samples in each treatment group Encapsome and Clodrosome? FACS analysis results should be evaluated statistically as differences in F4/80 and GRI positive cells do not appear to be significant.
49.
Several other questions persist about these experiments as it is confounding to find a localized defect in dentin and enamel that the authors claim are not related to osteoclastic resorption. Since bisphosphonates are known to suppress osteoclastic activities these results are confounding indeed. Furthermore, the authors may consider the phenomenon of internal resorption that is the result of resorptive cell activity that reside within dental pulp.
50.
Taken together, the concluding studies described in Figures 4 and 5 do not add much value to the manuscript as they are inadequately designed and poorly interpreted.
51.
Figure labeling is erroneous in multiple figure panels. This needs to be clarified for proper interpretation of findings.
52.
Did the authors notice any difference in spatial expression pattern of Csf1ligand and Csflr receptor in dental epithelium vs dental mesenchyme (Supplementary Figure 1d)?
53.
Statistical results for the TRAP assay should be added along with the clarification of whether the observed absence of osteoclast activity aligns with prior studies on clodronate.
Discussion: A revision of this section is recommended and should be done by providing as many mechanistic insights as possible into the role of tissue-bound macrophages present in tooth organs and surrounding tissues. As written, the authors repeat what is already presented in the Results while leaving out many interesting facets of these studies.
54. What is the relevance of this research to other tissues (e.g., bone, cartilage, skin), and what are the potential applications?
55. The current study is primarily based on mouse models, which raises questions about its applicability to human teeth. Could you elaborate on any potential clinical implications or translational outlook?
56. The paper discusses the transition of macrophages from the M0 (quiescent state) to the M2 (repair state), but the role of M1 (pro-inflammatory state) is not explored in detail. Could this be addressed further?
Animals: It is important to include the ratio of male to female embryos, young adult and adult mice used in these studies. Numbers of animals used in each group for each approach also need to be added.
7
Methods:
57.
Regarding the use of bisphosphonate drugs as a means to study pharmacologic macrophage depletion: Given the mixed therapeutic targets of bisphosphonates, it is difficult to determine that the drugs are directly linked to macrophage function in this system. Thus, it may be a stretch to attribute the transient phenotype observed after injection of these drugs to the 'depletion of macrophages' around the teeth. Even though TRAP staining was performed, proving the existence of some osteoclasts (but not necessarily their functional state), it would be more useful to study multiple other bone cells (osteoblasts, osteocytes) over a time course (pre-treatment, immediate post-treatment, x-days post treatment, etc.) alongside reliable markers of macrophages. This would allow one to better decipher the direct cellular link to this phenotypic change post treatment.
58.
Authors have mentioned that Clodrosome treatment led to severe aberrations in enamel and dentin morphology-please clarify specific aberrations such as changes in thickness, mineralization, or cellular organization. Include quantitative data.

Reviewer #2

(Remarks to the Author)
In this study, Lopez et al. sought to study the non-immune role of macrophages in dental development. Using growing teeth of a few mouse models as study models, the authors found that macrophages are associated with both epithelial and mesenchymal stem niches throughout lifetime. The mouse models used in this study include Wnt1cre; Csf1 fl/fl, Csf1R KO and Clorodonate-treated mouse as the pharmacological depletion of macrophages model. The teeth of these animals exhibit abnormal dentin and enamel deposition, eruption defects and tooth mispatterning. The phenotypes in the Csf1R KO can be partially rescued by bone marrow transplantation. The depletion of macrophages by clorodonate causes striking but temporary defects to the dental tissues. From these results, the authors propose that macrophages have essential roles in dental development and patterning of both epithelial and mesenchymal parts of the tooth.

I found this study is highly comprehensive and significant. The concept of the non-immune role in tooth development is extremely novel. Additionally, the manuscript is well-written. However, there are some points regarding the study's rigor that I would like to point out as listed below;

Major points:

Overall, all the animal models used in this study seem to have significant limitations to target macrophage cell population only directly and specifically. The evidence shown in this manuscript showing that targeted cells are specifically macrophages are not particularly strong and need further experiments to support the conclusion.

The authors started with the identification of the Csf1 ligand and its receptor Csf1R within the developing teeth and found that they are ubiquitously express in both mesenchyme and epithelium. (sup fig.1) They selected to use Wnt1cre driver mouse to remove Csf1 in the mesenchymal cell lineage. The rationale is unclear. Will removing Csf1 in epithelial lineage give rise to the comparable results? Are these cells limited to macrophages? As dental development is epithelial-mesenchymal interaction, changes in one compartment can affect the other compartment.

For example, in fig.2 Are the cells that co-express Csf1 and derived from Wnt1-cre express markers for macrophages only or other cells markers too? Another interesting point in the fig.2 is that AIF1+ cell shows different residing locations between incisor and molar, do you have any thoughts on these different phenomena?

In fig. 3b, other populations such as glia, epithelium, and non-labelled population in the middle of the panel also express Csf1R too.

The rescued result of transplantation experiment is very exciting. Are populated AIF1+ cells from the donor or the host? The quantification of these cells will enhance the strength of the study.

Data in the fig.4 is also remarkably interesting and exciting. However, I hope to see stronger evidence that this result is directly caused by the depletion of macrophage population. What are the mechanistic insights occurring at the macrophages- does the apoptotic macrophage cell number increase vs mesenchymal/epithelial apoptotic cell number, etc.?

Minor point

a. Page 6 line 15, do you mean fig 2i instead of 2k?

Reviewer #3

(Remarks to the Author)
-

Version 1:

Reviewer comments:

Reviewer #2

(Remarks to the Author)
I am satisfied with the revision and additional experiments and data the authors provided to my previous concerns and questions. The manuscript was significantly improved with stronger evidence and reads really well, currently. This study is very exciting and will help advance the knowledge of research field.

I also appreciate the opportunity to re-evaluate this manuscript alongside the detailed concerns raised by Reviewer #3. This study addresses an important and underexplored aspect of tooth biology by examining the role of macrophages in tooth development and homeostasis. The work proposes a new axis in which macrophages contribute to tooth development, expanding the traditional view that has largely focused on epithelial–mesenchymal interactions. While I agree with several of Reviewer #3's scientific concerns and believe certain interpretations require more cautious framing and clarification, I also find the overall observations to be novel, conceptually interesting, and potentially impactful for the field.

1.The delineation between bone-dependent and tooth-dependent effects - I do observe alteration in mandibular bone morphology, so I think that bone-dependent effects cannot be completely excluded. The current data support a strong

association between macrophage depletion and tooth pathology; however, the temporal and causal separation between bone changes and tooth defects remains incompletely resolved.

2. Resident vs circulating macrophages effect -The distinction between resident and circulating macrophages remains unclear. The manuscript emphasizes the importance of resident macrophages, yet the rescue experiment suggests that these cells are circulation-derived. I agree that this issue has not been adequately addressed and requires clearer explanation and terminology.

3. Supernumerary tooth - It appears that the authors emphasized this phenotype in response to Reviewer #1's previous comment. However, because this was observed in only 1 of 4 mandibles, I agree that it is overemphasized. The authors perhaps should explicitly state that this is a rare observation and present it as an isolated, interesting finding rather than evidence of a generalized patterning defect.

4. Reliance on histology and immunostaining instead of flow cytometry - I agree with Reviewer #3 that flow cytometry would provide a more quantitative assessment of macrophage populations. However, when performed with a well-validated and specific antibody, careful IHC quantification on serial sections can still provide reliable and biologically meaningful information, particularly in preserving spatial tissue architecture that flow cytometry cannot capture. In this context, the IHC analyses, if properly controlled and quantified, are sufficient to support the conclusions regarding macrophage presence and distribution in dental tissues.

5. Concern about descriptive nature and lack of mechanism - – I agree the work is largely descriptive and does not provide deep mechanistic dissection at the molecular level.

Despite these valid concerns, I believe this manuscript remains well suited for publication in Nature Communications. The study provides the first compelling evidence that macrophages are essential regulators of incisor epithelial architecture and continuous growth, revealing an unrecognized role for macrophages in tooth development. This expands the current paradigm beyond a purely epithelial–mesenchymal framework. The findings are conceptually novel, extend beyond dentistry into stem cell biology and epithelial morphogenesis, and open an important new direction for the field. With more cautious interpretation of certain phenotypes and clearer framing of macrophage origin and bone contributions, the work represents a significant and broadly relevant advance appropriate for Nature Communications.

Reviewer #3

(Remarks to the Author)
After reading the revised manuscript and an extensive response to reviewers comments, I am not able to confirm that manuscript is improved. This is mainly because the authors have missed many of the important points and gave inconclusive, indirect, or in some places incorrectly interpreted evidence to support their hypotheses.
The issue of suitability of the experimental models in delineating tooth-dependent effects from the bone dependent effect has not been resolved. Bone and tooth are intricately intertwined and interdependent, both during embryonic development and postnatal homeostasis. Alveolar bone differentiation and matrix secretion commence prior to elongation of the incisor, around E14(.5), and there are reports of various mouse mutants that display premature and accelerated bone differentiation associated with altered phenotype of the incisors. The authors provide no information on the timing of bone formation in Wnt1Cre; Csf1 flf/fl embryos, and the correlation with the onset of malformations in the developing incisors is missing. Therefore, the possibility that bone phenotype precedes and conditions tooth development and that changes observed in the incisor are secondary changes cannot be dismissed. I disagree with their statement that the jaw in these mutants is of correct shape and the overall fine structure of the jaw is maintained, because it is not supported by any evidence: Figure 3j shows mutant mandible of significantly decreased size and altered shape when compared to control (3i). Without quantification analysis there is no proper evidence to support their conclusion, particularly in the light of insufficient sample size (two 6 weeks old and two 8 weeks old samples for Wnt1Cre; Csf1fl/fl line).

Further, the importance of the supernumerary tooth formation is overstated and inappropriately emphasized considering it is observed in only one sample out of 4. This conclusion requires bigger sample size, high frequency of the phenotype and deeper analyses of the embryonic tooth development in these mutants. For example, it is critical to know whether it is unilateral or bilateral change, and also whether macrophage distribution at appropriate developmental stages reflects differences between the normally toothless diastema region where they observe the extra tooth and the rest of the dentition. Without these analyses, this phenotypic change should be considered an observation, rather than a conclusive evidence of the impacted early development and patterning.
Overall, judging by the more penetrant phenotypic changes of their mutant lines, as well as the treated animals, it is very likely that they are secondary to the alterations in mutant mandibular bone (i.e. bone turnover) that hamper continuous growth of the incisor, hence the folding.

Discerning the role of resident macrophages separately and independently of circulatory macrophages is also not resolved in the resubmitted manuscript. It seems that the authors refer to resident macrophages as the population found in the dental tissues. However, their rescue experiment shows that this population derives fully from the circulation, which is in line with the Csfr1 knockout that shows more penetrating phenotype than the Csf1 conditional knockout.

I was disappointed to see that the authors remained fixed on using histological sections and unreliable quantification in immunostained sections to analyse the effect of mutations or treatments on macrophage population in dental tissues. The

credibility and accuracy of their data would be significantly increased if they used flow cytometry, particularly in case of overexposure and high background which exist in their immunostainings: many of the cells show nuclear staining for what should be surface marker and co-localization studies are not of best quality. Flow cytometry would provide a great depth to their study. Similarly, lack of mechanistic studies undermines their conclusions, and computational modelling of cell-cell and molecular interactions does not provide direct or conclusive evidence. These downsides emphasise the overall descriptive quality of the study and speculative character of the conclusions, which in my opinion is not suitable for publication in Nature Communications.

Reviewer #4

(Remarks to the Author)
Per the editor’s request, I am commenting on the authors’ response to Reviewer #1. In my view, most of the major concerns raised by Reviewer #1 have been adequately addressed in the revised manuscript.
1. The understanding of the complex biological systems driving tooth formation and eruption has been improved with revised text and additional experiments.
2. I agree with the authors that the main focus of this study is to elucidate the role of macrophages in tooth, leveraging mouse models with disruptions in the Csf1-Csf1r axis and pharmaceutical depletion of macrophage; the exact action mechanisms of individual molecules or cytokines are not the focus of this study.
3. However, the distinction between tissue-resident macrophages and monocyte-derived macrophages remains unclear in the revised manuscript. In the field, tissue-resident macrophages typically refer to cells originate during embryonic development and primarily maintain organ homeostasis under steady-state conditions. Monocyte-derived macrophages arise from circulating monocytes, are recruited to tissues, and generally function in pathogen defense or tissue repair. Distinguishing whether a macrophage population is tissue-resident or monocyte-derived is indeed challenging. Nevertheless, several established approaches can help address macrophage origin, including lineage tracing, marker expression (may be tissue-dependent), monocyte depletion/transfer, and scRNA-seq identity and trajectory analyses. While the authors have provided additional data, the manuscript does not interpret these data with sufficient clarity regarding macrophage origin. This likely reflects an incomplete understanding of the definitions and experimental approaches used to distinguish tissue-resident from monocyte-derived macrophages. The interpretations of the data regarding this point should be made cautiously and clearly based on the presented results and existing literature, to avoid the confusion to the readers.
4. Minor suggestion: The abstract could be more informative, particularly in the pharmacological depletion section, and would benefit from more clearly specifying the role of macrophages in teeth.

Version 2:

Reviewer comments:

Reviewer #4

(Remarks to the Author)
The authors have thoroughly addressed my comments, and the manuscript's quality has improved.

Open Access This Peer Review File is licensed under a Creative Commons Attribution 4.0 International License, which permits use, sharing, adaptation, distribution and reproduction in any medium or format, as long as you give appropriate credit to the original author(s) and the source, provide a link to the Creative Commons license, and indicate if changes were made.
In cases where reviewers are anonymous, credit should be given to 'Anonymous Referee' and the source.
The images or other third party material in this Peer Review File are included in the article’s Creative Commons license, unless indicated otherwise in a credit line to the material. If material is not included in the article’s Creative Commons license and your intended use is not permitted by statutory regulation or exceeds the permitted use, you will need to obtain permission directly from the copyright holder.
To view a copy of this license, visit https://creativecommons.org/licenses/by/4.0/

Thank you again for submitting your manuscript "Embryonic and postnatal macrophages are essential for dental morphogenesis and self-renewal" to Nature Communications. We have now received reports from 3 reviewers and, after careful consideration, we have decided to invite a major revision of the manuscript.
As you will see from the reports of Reviewer #1 and Reviewer #2 copied below, the reviewers raise important concerns. We find that these concerns limit the strength of the study, and therefore we ask you to address them with additional work. Without substantial revisions, we will be unlikely to send the paper back to review. If you feel that you are able to comprehensively address the reviewers' concerns, please provide a point-by-point response to these comments along with your revision. Please show all changes in the manuscript text file with track changes or colour highlighting. If you are unable to address specific reviewer requests or find any points invalid, please explain why in the point-by-point response.

Reviewer #3 provided brief comments to the editor only which included general remarks on the manuscript followed by a few technical remarks. This reviewer pointed out that the analysis of the macrophage population in the developing tooth is based on a single marker and that expanding the marker selection and provision of quantitative data would strengthen the results. They also expressed the same concerns as Reviewer #2 about limitations the animal models used in this study. Lastly, they pointed to discrepancies in the analysis of pharmacological model of macrophage depletion as they found that the results and M&M sections differ in description of the age, administration strategy and dosage of the drug used for different analyses.

Regarding the remarks to the editor provided by Reviewer #3, editorially, we are providing them to you for transparency and request that at resubmission you include a summary of revisions that address the technical concerns of this reviewer in the cover letter.

We are grateful to all three reviewers for their valuable feedback, which has substantially improved the quality of our manuscript. We have carefully addressed all comments and provide here a detailed response letter along with a thoroughly revised version of the manuscript. More specifically, we introduced significant changes in all figures, accordingly rewrote introduction, results, discussion and methods, produced 4 completely new figures (Fig. 2 and 4; Supplementary Fig. S1 and S4) and 2 new tables. Our updated version includes fundamentally new data and findings, as well as extensive supporting evidence that further expands and strengthens our original conclusions.

**MAJOR NEW EXPERIMENTS PERFORMED**			
**Research question addressed**	**Summary description**	**Figure**	**Method**
**Improved visualization of macrophages colonization in tooth-associated structures adjacent**	Lower- magnification images of embryonic and postnatal stages of tooth development to include key regions involved in eruption and root formation.	Figure 1a-g	Immunohistochemistry (AIF1) and RNA *in situ* hybridization (*Csf1, Csf1r)*

**during pre-eruptive and post-eruptive stages**	Including detailed insets in critical in the eruption space, dental follicle, alveolar bone and interradicular area.	Figure 1d´,e´, Supplementary Figure 1	Immunohistochemistry (AIF1, KI67, CTSK) and RNA *in situ* (*Csf1, Csf1r*) hybridization
	Spatial delineation of macrophages in mouse incisor according to different cell types or structures associated	Figure 1i,j Supplementary Figure 3	Immunohistochemistry (AIF1, KI67) and RNA *in situ* (*Csf1, Csf1r*) hybridization
**Interaction network between macrophages and other dental cell types**	Quantification of intercellular communication between cell clusters	Figure 2a	Bioinformatic analysis: CellChat (Jin et al, 2025)
	Analysis of macrophages interacting with other cell types as SENDERS	Figure 2b	Bioinformatic analysis: CellChat (Jin et al, 2025)
	Analysis of macrophages interacting with other cell types as RECEIVERS	Figure 2c	Bioinformatic analysis: CellChat (Jin et al, 2025)
	Identification of ligand-receptor pairs based on prediceted communication probabilities between macrophages and other cell types	Figure 2d	Bioinformatic analysis: CellChat (Jin et al, 2025)
	Validation in tissue sections of proposed Cd44-Lgals9 pair (stellate reticulum/stratum intermedium ↔ macrophages)	Figure 2e	Immunohistochemistry: co-localization CD44 + AIF1
**Delineation of the cause of the observed tooth phenotypes as tooth-autonomous, bone-driven or combination**	Characterization of mandibular bone thickening in $Wnt1^{Cre}/Csf1^{fl/fl}$ mice	Figure 3	MicroCT
	Assessment of epithelial and vascular defects in incisors and molars of $Wnt1^{Cre}/Csf1^{fl/fl}$	Figure 3, Supp. Fig. 4	Immunohistochemistry: co-staining COL4 + CDH1
	Evaluation of bone remodelling abnormalities in the alveolar bone-tooth interface in $Wnt1^{Cre}/Csf1^{fl/fl}$	Figure 4	TRAP, Masson, immunohistochemistry (co-staining AIF1 + CTSK)
**Investigation of the dental and bone phenotypes induced by bone marrow transplantation (BMT)**	Assessment of successful engraftment and macrophage repopulation following BMT	Figure 5f-h	Immunohistochemistry (co-staining GFP + AIF1)
	Evaluation of the dental rescue morphology and mineralization	Figure 5l,m,n,p,q,r	MicroCT
	Evaluation of epithelial integrity and enamel production in *Csf1r KO* for correct enamel production	Supp. Figure 4e-i	MicroCT and immunohistochemistry (COL4, CDH1, CALB1)
	Characterization of jawbone structure and morphology recovery after BMT	Figure 5l,m,n,o	MicroCT
**Elucidation of the consequences of macrophage depletion by bisphosphonates on tooth, bone, and inflammation**	Validation of macrophage population reduction in mouse incisors following Clodrosome treatment, including pulp and epithelium.	Figure 6b-g	Flow cytometry (F4/80, Gr-1), immunohistochemistry (AIF1, F4/80)
	Characterization of the cytokine and interleukins profile after Clodrosome	Figure 6o-p	Cytokine array assay, single cell transcriptomics
	Quantification of short- and long-term changes in osteoclast activity after Clodrosome treatment.	Figure 6n	TRAP assay
**Validation of Aif1 and Csf1r as specific markers of macrophages in dental tissue**	Identification and characterization of Csf1r+ and Aif1+ cells co-expressing general macrophage markers in dental tissues.	Supp. Fig 2d, Supp. Fig 3, Figure 6e	Single cell transcriptomics, RNA *in situ* hybridization, immunohistochemistry: AIF1, F4/80

Reviewer 1

As evidenced in the literature, the development of dentition involves a complex interplay of transcription factors, growth factors and extracellular matrix molecules whose coordinated activities control the position, shape and size of teeth. Signaling pathways also control the formation of specialized matrices and the eruptive pathways that allow for an encased tooth to erupt from within its bony crypt into the oral cavity. In this manuscript the authors draw attention to a relatively unexplored role for macrophages during early to late phases of tooth development Using multi-pronged analyses, they propose that resident macrophages a sub-population of macrophages that are non-circulating in nature, do play key roles in tooth morphogenesis, homeostasis and regeneration. The well characterized murine molar and (continuously erupting) incisor are the models in these studies that utilize scRNA-seq and FACs analyses, genetic ablation using traditional and conditional approaches as well as pharmacological interventions aimed at depleting macrophages *in vivo*. The authors conclude that long-lived or resident macrophages are present in both epithelial and mesenchymal compartments of tooth organs and are likely to also modulate homeostasis within stem cell niches at the apical end of growing incisors. They further postulate a non-immune role for these macrophages during the process of tooth eruption.

Areas of major concern:

1. As written, the manuscript suffers from a (perceived) lack of understanding of the biological systems that drive the formation of teeth and their subsequent eruption. Such an in-depth understanding is needed for a more meaningful interpretation of the results and must lead to an improved quality of the data sets presented.

We thank the reviewer for their valuable feedback, and we agree that a more in-depth understanding and additional experimental results are needed to properly understand the role of macrophages during pre-eruptive and post-eruptive stages as well as during root elongation.

For this reason, we largely focused this experimental revision towards addressing which part of the phenotype is dependent on arising tooth eruption problems and alveolar bone remodelling issues, which part is tooth autonomous, and where we observe the joint action of bone and teeth generating the abnormality driven by the loss of macrophages. Sharing the same affection for the bone, we listened carefully to the reviewer's arguments and thank the reviewer for pushing us towards investigating the bone part much deeper. We hope the reviewer agrees that in the end our manuscript became stronger. Everywhere, where it was possible, we opted for designing and performing the additional experiments.

This line of new investigations helped us to delineate the parts of the observed CSF-1 depletion phenotype, which are tooth-independent in a clear way. Those examples include: the integrity of enamel and dentin in adult animals (when the phenotype is induced already after eruption), as well as early dental patterning (at the level of induction of supernumerary teeth). There are several points why we state that such observed dental defects are a tooth-autonomous phenotype and not a result of problems in bone development:

1. Alveolar bone of the mouse jaws from *Wnt1*^{Cre}/*Csf1*$^{fl/fl}$ mice can develop in embryos into a correct shape maintaining the overall fine jaw structure (shown in microCT scans in **Figure3i,j**).

2. Another supporting reasoning is the observation of supernumerary teeth (marked and labelled in **Figure 3j**) next to the first molar. This observation confirms that the lack of macrophages actually leads to a dysregulation directly into the normal **early tooth development and patterning (before the bone development)**, and not as a consequence of a bone-related defect.
3. Moreover, the pharmacological induced macrophage depletion triggering transient odontoblast and epithelial disruptions in adult mice sustains that macrophage cluster is key to maintain tooth cell integrity and functionality (**Figure 6h-k**).
4. The bone transplantation experiment in adult animals results in partial rescue of the phenotype.

Taking together, we are confident now that the supporting bone surely has a contribution to the observed tooth-phenotype (delayed eruption and root elongation in *Wnt1*^{Cre}*/Csf1*$^{fl/fl}$ and *Csf1r KO*, dismorphic cusps) but taking in consideration the above-mentioned findings during this revision, we conclude that the lack of macrophages in the dental tissue is also the cause of the specific and tooth-autonomous dental defects, which differ from defects induced by bone-related issues. We thank the reviewer for helping to figure this out.

In the main text, we formulate this conclusion in the following way:

Page 16, lines 18-34; Page 17, lines 1-7: *"In this study, we provide strong evidence that macrophage depletion leads to tooth-autonomous phenotypes, such as the formation of supernumerary teeth in Wnt1Cre/Csf1fl/fl mice and transient disruptions in enamel and dentin following prolonged Clodrosome treatment. However, we also observed that disruption of CSF signaling alters normal bone formation, resulting in delayed tooth eruption and root elongation in both molars and incisors. The development of alveolar bone and teeth represents a highly integrated and co-regulated process within the embryonic craniofacial complex. These tissues do not form independently but arise through continuous molecular, biomechanical, and immune system-derived interplay (Yuan & Chai, 2019; Mori et al, 2013). In particular, macrophages and their CSF1-dependent lineages emerge as key mediators within this interface, orchestrating osteoclast differentiation, bone resorption, tooth eruption, as well as intra-dental epithelial and mesenchymal cell responses. This intricate coupling means that perturbations affecting bone formation and immune cell recruitment to facial tissues during development are unlikely to remain confined to one compartment. Instead, they propagate across the entire alveolo-dental complex, altering root elongation, eruption dynamics, and even enamel and dentin maturation. Therefore, a mechanistically holistic approach - one that jointly examines odontogenic and osteogenic processes and integrates the contributions of resident immune and stromal cells - is essential for understanding the orchestration of orofacial development. In this specific context, studying bone and tooth formation in isolation risks overlooking the reciprocal dependencies that define this morphogenetic continuum and shape both normal development and pathological outcomes. At present, it is not possible to clearly delineate whether all the observed phenotypical manifestations of the mutant mice (shorter incisors, pulp chamber filled with dentin, multiple folded or discontinuation dental epithelium etc.) arise from bone-driven effects, tooth-intrinsic mechanisms, or a combination of both, as current in vivo methodologies do not allow the study of these processes in complete isolation. Further research will be required to disentangle these interdependent mechanisms."*

Below we additionally list the experiments that we are providing during this revision for building a more in-depth view of the alveolar bone in CSF1 depletion, wild type animals and other experiments: First, in this revision, we provide a more in-depth visualization and description in wildtype animals about the spatio-temporal progression/establishment of AIF1+ cells in the zones critical for tooth development and eruption (superior wall of the alveolar crypt, dental follicle) during embryonic development (E15.5, E17.5) and later postnatal stages (P0, P6, P14) as seen in the new **Figure 1a-g** and **Supplementary Fig.1 a-c.** We comment on this in the text as follows:

Page 5, lines 26-31*: "…Besides the dental papilla/pulp mesenchyme, the presence of AIF1^{+} cells was also observed in the close surroundings of the tooth, including the superior wall of the alveolar crypt (the space between the enamel organ and the oral epithelium) and the dental follicle at different time points (Fig. 1b–g; Supplementary Fig. 1a–c). The presence of AIF1+ cells was also detected during later root elongation process at P14 in the dental follicle root-growing space (Fig. 1g)."*

Moreover, as tooth formation cannot be addressed as an isolated event, we characterized in detail the surrounding alveolar bone by detecting the presence of osteoclasts using cathepsin K (CTSK) antibody at postnatal stages (P0, P6, P14) in the location above the cusps (eruption space) and in the root growing space. These results are presented in the revised version of manuscript in **Supplementary Fig.1**, and commented in the manuscript, as follows:

Page 5, lines 31-34: *"…AIF1+ cells as monocyte-lineage derivatives, share common lineage with osteoclasts. These cells, characterized by expression of Cathepsin K (CTSK) (Dai et al, 2020; Wilson et al, 2009) are essential for bone resorption allowing root elongation and tooth eruption (Fig. Supplementary 1d-f)."*

The advice of the reviewer helped us to realize that the alveolar bone surrounding the teeth should not be unnoticed and does not remain passive when affecting CSF-1 signalling. Our newly produced data presented in the new **Figure 4** shows that the delay in molar eruption and root formation is a consequence of disrupted monocyte-to-osteoclast differentiation and migration.

Crucially, we expanded our study utilizing *$Wnt1^{Cre}/Csf1^{fl/fl}$* mice model with a postnatal day 14 developmental stage, which was not included in the previous version of the manuscript. The postnatal day 14 was chosen to showcase timing to characterize changes in the bone and epithelium present above the first maxillary incisor and the root growing zone, as well as the changes in the alveolar bone resorption surrounding the apical part of the incisor. **Our new results emphasize the crucial role of CSF signalling in monocyte-to-osteoclast differentiation for proper alveolar bone resorption above the cusps as well as for root elongation – processes that are vital for tooth eruption.** Schematized in the **Figure 4j**.

These new conclusions are based on newly performed co-staining and quantifications for osteoclasts (CTSK+) and macrophages (AIF1+) in the above-mentioned locations and showed side-to-side with conventional histological staining, such as TRAP and Masson Trichrome, presented at the new version of **Figure 4a,b**. Finally, we analysed morphometric parameters of the molars (measurement of the distance from cusps to oral epithelium and root length) shown **Figure 4c-g**. We commented on this in the manuscript, as follows:

Page 8, lines 13-26: *"…To further elucidate this phenotype, we conducted a detailed characterization of the jawbones in these animals (Fig. 4). Molar eruption and root formation were developmentally delayed (Fig. 4a,b). To quantify these defects, we measured the number of AIF1+, CTSK+ and AIF1+/CTSK+ positive cells in the eruption space and root growth space (Fig. 4c–e), as well as morphometric parameters including eruption distance and root length (Fig. 4f,g). Our results show decrease of AIF1+ cells (macrophages) in mutant mice, however no significant difference was observed in the number of CTSK+ (osteoclasts) or AIF1+/CTSK+ (pre-osteoclasts) cells between mutant and wildtype animals (Fig. 4d,e). Subsequently, we examined the alveolar bone socket of mouse incisor. In contrast to molars, the incisors of mutant mice exhibited a significant increase of AIF1+/CTSK+ cells (pre-osteoclasts) and in parallel a significant decrease of CTSK+ cells (osteoclasts) (Fig. 4h,i) along with a notably wider dental follicle space (haematoxylin-eosin images of Fig. 4h). These findings explain more prominent phenotype observed in incisors compared to molars (Fig. 3i,j) and suggest the important role of CSF1 signaling for proper differentiation of osteoclasts (Kim & Kim, 2016; Mun et al, 2020) (Fig. 4j)".*

Our new data indicate that the observed developmental tooth phenotype results rather from delayed root growth rather than from disorders associated with alveolar bone resorption during tooth eruption. We now discuss this as follows:

Page 14, lines 27-36; Page 15, lines 1-4: *"…It is worth noting that $Wnt1^{Cre}/Csf1^{fl/fl}$ animals also exhibited additional craniofacial abnormalities. While the overall architecture and size of the upper and lower jaws were preserved, the bone displayed increased thickness, likely resulting from impaired osteoclast differentiation and function. The delayed molar eruption observed in $Wnt1^{Cre}/Csf1^{fl/fl}$ mice aligns with previous studies demonstrating that* ***local administration of CSF-1 accelerates tooth eruption and modulates bone remodeling through osteoclastogenesis*** *(Cielinski et al, 1995; Wise, 2009). CSF-1 plays a critical role in supporting the recruitment of mononuclear cells (osteoclast precursors) to the dental follicle prior to the onset of eruption (Wise et al, 2002, 2005). These findings emphasize the importance of CSF-1 signaling for the coordinated interaction between the dental follicle and the surrounding bone to achieve correct tooth shape. This finding is linked to the neural crest origin of the majority of cranial bones (Santagati & Rijli, 2003). From this perspective, the dental phenotype observed in $Wnt1^{Cre}/Csf1^{fl/fl}$ animals should not be considered as an isolated defect, but rather as a part of a broader phenotypic manifestation. However, our data indicate that the dental defects arise as a primary disorder rather than as a secondary effect of surrounding bone pathology."*

Finally, we allocated more space in the manuscript to introduce and discuss the importance of the alveolar bone and eruption process for the correct dental shape, also providing additional references. We additionally introduce a discussion passage into the main text, highlighting that alveolar bone and teeth develop in an intricate and highly integrated complex, and they shall be investigated jointly to avoid a less-holistic and isolated understanding of the orofacial development:

Page 15, lines 23-34; Page 16, lines 1-2: *"The development of alveolar bone and teeth represents a highly integrated and co-regulated process within the embryonic craniofacial complex. These tissues do not form independently but arise through continuous molecular, biomechanical, and immune system-derived interplay. In particular, macrophages and their CSF1-dependent lineages emerge as key mediators within this interface, orchestrating osteoclast differentiation,*

bone resorption, tooth eruption, as well as intra-dental epithelial and mesenchymal cell responses. This intricate coupling means that perturbations affecting bone formation and immune cell recruitment to facial tissues during development are unlikely to remain confined to one compartment. Instead, they propagate across the entire alveolo-dental complex, altering root elongation, eruption dynamics, and even enamel and dentin maturation. Therefore, a mechanistically holistic approach - one that jointly examines odontogenic and osteogenic processes and integrates the contributions of resident immune and stromal cells - is essential for understanding the orchestration of orofacial development. In this specific context, studying bone and tooth formation in isolation risks overlooking the reciprocal dependencies that define this morphogenetic continuum and shape both normal development and pathological outcomes."

2. Taken together, the distinction between tissue-resident macrophages and those derived from circulation (monocytes) is not clearly established in these studies. Moreover, the results fall short in demonstrating that these cells serve non-immune functions.

We appreciate the reviewer´s comment. Indeed, the distinguishing the tissue-resident and monocyte-derived macrophages is not an easy task. Our new data (in particular the bone marrow transplantation part) show that the population of dental tissue resident macrophages can be promptly re-established after bone marrow transplantation (**Fig. 5e-h**). This points out at the remarkable cellular plasticity of the whole monocyte/macrophage system and importance of its tissue/organ role. We address this, in revised manuscript we provide better clarification as follows:

Page 9, lines 13-23: "…*Young Csf1r KO animals (in contrast to shown data for Wnt1^{Cre}/Csf1$^{fl/fl}$ adult ones) were used to cover the whole developmental process of teeth to match the phenotype rescue experiment explained below. To demonstrate that the absence of macrophages is the causative factor leading malformations of teeth, we performed a phenotype rescue experiment. We conducted a bone marrow transplantation (BMT) via single intraperitoneal (i.p.) administration of a healthy mouse GFP+ whole bone marrow into P1 mice (Fig. 5e-h). To assess the success rate of BMT in relation to dental tissue, we quantified the engraftment of the transplanted cells by IHC co-staining of AIF1 and GFP. Although in the dental tissue of Csf1r KO animals we did not observe any AIF1+/GFP+ cells, after BMT the number of AIF1+/GFP+ cells increased to 52.15 cells per region of interest (ROI) in the dental pulp. This demonstrated the efficient infiltration and establishment of transplanted cells within the dental pulp and their contribution in the phenotype rescue (Fig. 5h).*"

We discussed about these results in the main text accordingly:

Page 15, lines 11-17: "...*Although Csf1r KO animals establish teeth, they display severely dysmorphic tooth phenotypes. Bone marrow transplantation by adoptive transfer replenishes macrophages in both molars and incisors, partially restoring the morphological phenotype, which indicates a cell-autonomous contribution of macrophages. Along with the dental phenotype rescue, the bone marrow transplantation partially compensates the inefficiency of Csf1R KO mice to achieve correct osteoclast differentiation, visible by an overall improvement of the mandibular and maxillary jawbone shape and density".*

Additionally, to better clarify the population of tooth-resident macrophages in the updated version of the **Figure 1** we included a new panel (**Figure 1k**) where we delineated and labelled the different macrophages found in the incisor based on their specific spatial distribution at early postnatal stage (day 6) utilizing AIF1 marker staining. This result shows in detail that macrophages exhibit various associations with different histological compartments: blood vessels-associated, odontoblast-associated, dental follicle-associated, dental epithelium-associated and others, some having proliferative potential giving arise to new progeny of macrophages within the dental tissue (**Figure 1i,j**). These results are commented in the main text of the manuscript as follows:

Page 6, lines 1-7: *"… Similar to the molars, macrophages populated the dental pulp of the continuously growing mouse incisor (Fig 1i-j). Co-staining of macrophages with mKI67, a marker of proliferating cells, revealed their proliferative capacity within the teeth, suggesting that these cells are pulp-resident and maintained through local proliferation. (Fig. 1i). The spatial distribution of macrophages in this tooth was not stochastic, but have shown an association with other cell types and histological structures such as neurovascular bundle, the odontoblast (and sub-odontoblast) layer, dental pulp cells or dental follicle region (Fig. 1j)."*

To further spatially delineate the distribution of *Csf1* and *Csf1r* during incisor and molar development we updated the **Supplementary Figure 3** which now better visualize spatio-temporal distribution of *Csf1* and *Csf1r* in the dental tissue from pre-natal to postnatal stages with emphasis on annotation of spatial distribution with color-coded semi-transparent regions, arrowheads pointing on the positive region and new details on the presence of macrophages in progenitor area of incisor and pulp horn the molar at P0 and P14 developmental stages. We adapted the text as follows:

Page 7, lines 6-9: *"… The expression of the Csf1 in the dental tissue was not restricted to the adult stages only, but both Csf1 and Csf1r were present in the developing molars and incisors within dental papilla/pulp, dental follicle, odontoblast layer and inside of the dental epithelium (Supplementary Fig. 3)."*

Naturally, the specific role/function of the macrophages is highly dependent on their spatial distribution and microenvironment. The data presented in this manuscript clearly shows evidence that macrophages are fundamental cells for reaching correct tooth development and its homeostasis during adulthood. This idea is supported by the pharmacological depleted experiments (**Figure 6**) in which the time-controlled depletion of macrophages created a transient disruption of the odontoblast and ameloblast layer implying defects in dentin and enamel (shown by microCT and histology – **Figure 6h-k**.

Another supporting argument are the epithelial defects observed in the genetically-modified mice models (**Figure 3k,l** for *Wnt1/Csf1*$^{fl/fl}$, in **Supplementary Fig. 4a,b** and *Csf1r KO* in **Supplementary Figure 4e,f**) supporting the idea that macrophages do not serve simply as immune cells but are essential for the maintenance of the microenvironment of tooth cells.

In our new manuscript we adapted the discussion as follows:

Page 14, lines 21- 26:*"…The mechanism by which macrophages control tooth development could involve their phagocytic activity, which may be either specifically or non-specifically targeted on recycling signalling molecules from the Wnt, Bmp or Fgf signaling pathways that are essential*

for tooth morphogenesis (Liu et al, 2008; Raju et al, 2024; Aberg et al, 1997; Liu et al, 2022). Importantly, the observed phenotype is not attributable to the Wnt1^{Cre} background itself, as animals lacking Csf1$^{fl/fl}$ allele do not exhibit any dental defects (Lewis et al, 2013)."

In the Discussion section of the paper, we commented the possible ways how macrophages can modulate the microenvironment in the dental context – via engulfing cellular debris or apoptotic cells, or releasing growing factors or cytokines essential to maintain the dental cell niche healthy and functional- and connected with existing literature in other tissue types.

3. While the study focuses on CSF-1 and its receptor with the intent of unravelling their roles in modulating the activation, migration and phagocytosis activities of macrophages, such a delineation of CSF-1 stage-specific functions is not provided.

We thank the reviewer for this comment. Indeed, although Csf1-Csf1r signalling is an important component of this manuscript, our goal was not to study in detail the mechanisms of macrophage modulation, migration, and phagocytosis. Instead, we took advantage of the availability proved functionality of these selected mouse models to investigate the consequences of macrophage depletion in teeth, without further focus on the exact mechanism of action. Unfortunately, a detailed studying of these mechanisms was substantially beyond the scope of this project.

We employed mouse models failing in the Csf1-Csf1r signalling axis (either neural crest-specific tissues or global knock out) as a tool to elucidate the role of macrophages on the tissue level since these mice have been shown in the literature to fail in the correct recruitment and establishment of monocyte-derived cells in other tissues, such as the case of microglia in the brain (Bennett *et al*, 2018).

Although the goal of this study was not unravelling the role of Csf1-Csf1r signalling, we improved on bone marrow transplantation experiments to observe how macrophages return to the facial bone and teeth in depleted models. The analysis of this experiment now gives more valuable information about re-settlement of macrophages (**Figure 5l-r**).

Based on this comment to better delineate the stage-specific spatial distribution of *Csf1r+* macrophages and *Csf1* ligand during tooth development. To provide a better insight of this, we revised the **Supplementary Figure 3** which now better visualize spatio-temporal distribution of *Csf1* and *Csf1r* in the dental tissue from pre-natal to postnatal stages with emphasis on annotation of spatial distribution with color-coded semi-transparent regions, arrowheads pointing on the positive region and new details on the presence of macrophages in progenitor area of incisor and pulp horn the molar at P0 and P14 developmental stages. The Results were changed accordingly:

Page 7, lines 6-9: *"... The expression of the Csf1 in the dental tissue was not restricted to the adult stages only, but both Csf1 and Csf1r were present in the developing molars and incisors within dental papilla/pulp, dental follicle, odontoblast layer and inside of the dental epithelium (Supplementary Fig. 3)."*

4. It is unclear why these studies focus on CSF-1 alone when it is well known that multiple cytokines such as the interleukins, TGF-beta, TNF, PTH and RANKl also influence osteoclastogenesis, bone

resorption and bone remodeling that are essential in late phase of tooth development during root elongation and tooth eruption. Furthermore, the question of how resident macrophages interact with T lymphocytes and dendritic cells that are present within dental pulp and the periodontal ligament and that also play an important role in immunosurveillance and homeostasis. scRNA-seq methods should make it possible to include a more holistic approach to understanding the micro-environment that dictates the formation, function and fate of resident macrophages throughout the life cycle of a tooth.

We thank the reviewer for bringing it up. For us, CSF1 depletion was purely instrumental. Indeed, the goal was not focusing on CSF-1 or other immune system signals (they are highly complex and combinatorial in the immune system), but to take advantage of the CSF1-CSF1R axis because it is the most effective and specific way to reach macrophage depletion selectively in areas of teeth formation, which are neural crest-derived. Previously, the role of macrophages and monocytes in tooth induction and development was not established, and this is exactly what we cover here. The general depletion of monocyte and macrophage lineage in embryos can have multiple systemic and side effects, and we had to avoid such strategies. Instead, local and specific tissue knockout of CSF1 turned out to be a wonderful and effective instrument, also highly specific for macrophages. Indeed, Csf1 has been shown to be the primary growth factor for the control of monocyte and macrophage differentiation, survival, proliferation and renewal in various tissues. For this reason, the tissue specific mouse line (*Wnt1^{Cre}*/ *Csf1$^{fl/fl}$* for neural crest derivatives) allows us to study the changes in tooth development in a situation where macrophages are non-present in the neural crest-derived tissues, whereas their differentiation and presence are not affected elsewhere.

However, as pointed out by the reviewer, cytokines and interleukins play a crucial role in the macrophage context. We decided to follow the reviewer's advice and **investigate further on this, for which we performed a cytokine and interleukins array experiments** from the extracted pulp of bisphosphonates-treated animals and correlated with single-cell transcriptomics data of the same condition. The results are included in the updated version of **Figure 6o-p**, and commented as follows:

Page 12, lines 1-10: "…*Bisphosphonate administration has been reported to influence the production of both pro- and anti-inflammatory cytokines, likely through apoptotic macrophage clearance and subsequent immune modulation. in the short term, bisphosphonates reduce pro-inflammatory cytokine production, whereas in the long term, this effect is reversed (Khanna et al, 2024). . To investigate this phenomenon in our Clodrosome-treated mice, we employed two complementary approaches. First, pro- and anti-inflammatory cytokine levels were analyzed in isolated dental pulps of incisors using a targeted array profiling. This analysis revealed an overall downregulation of both cytokine classes (Fig. 6o). We then correlated these findings with scRNA-seq transcriptomic data from Clodrosome-treated mice, which similarly showed downregulation of key pro-inflammatory markers, including MIP1β, MIP1α, and TNFα (Fig. 6p), which was consistent with the cytokine assay on protein level*."

The second part of the reviewers' comment relates to the potential interaction between macrophages, lymphocytes T and dendritic cells. Firstly, we leveraged the knowledge about what is known about immune cells in the dental pulp and periodontal space to orient better the reader. These comments can be found in the text as follows:

Page 3, lines 25-35:"*Recent studies focused on independent classification of various dental cell types via methods of single-cell RNA-sequencing (scRNA-seq) have revealed a proportionally*

substantial amount of immune cells present in both human and mouse teeth with diverse expression profiles (Krivanek et al, 2020; Pagella et al, 2021). These include neutrophils, B- and T-cells, or macrophages in both dental pulp or periodontal space (Sharir et al, 2019; Krivanek et al, 2020; Takahashi et al, 2019; Chiba et al, 2020; Nagata et al, 2021; Hanson-Drury et al, 2023). Although, previously, the role of immune cells have primarily been studied in the context of inflammatory conditions as pulpitis or periodontitis, the above-mentioned scRNA-seq studies have worked with healthy dental tissues (Figueredo et al, 2019; Pohl et al, 2024). Beside the immune functions, emerging studies have shown macrophages to be key players in reparative dentinogenesis (Neves et al, 2020; Kadowaki et al, 2022) in both human and mouse teeth. In addition to macrophages, dendritic cells have been also shown to participate in immunosurveillance and antigen presentation during pulp inflammation (Chen et al, 2024; Pohl et al, 2024; Quispe-Salcedo & Ohshima, 2021)."

Thus, by using single-cell transcriptomics data we investigated in a more holistic approach the interaction potential of different cell types in tooth as immune cells but probability with other cell types, while keeping the focus on macrophage cluster. In response, we included an interactome analysis in our study. This analysis covers the number of interactions (senders and receivers), as well as the communication probability inferred from receptor-ligand pairs based on our single-cell dataset between the macrophages and several other cell populations in the tooth. Although, the results obtained by this more holistic approach showed that macrophages and T-cells are among the less interactive cell clusters. This new data can be found as a new **Figure 2**, and commented in the main text as follows:

Page 6, lines 9-29: *"…In tooth, similar to other organs such as mammary gland or salivary gland, resident macrophages are in close contact with heterogenous cell populations as integral part of the microenvironment (Zhou et al, 2023; McKendrick et al, 2023). Based on previous single cell transcriptomics data (Krivanek et al, 2020) we delved into the interaction and cell communication between macrophages and the rest of cell clusters present in the mouse incisor. Although, macrophage cluster is not among the most interactive cell groups, a range of 25-50 interaction partners mainly with dental follicle, stellate reticulum (SR), stratum intermedium (SI) and outer enamel epithelium (OEE) was detected (Fig. 2a). The subsequent analysis of separate signals revealed the interaction probability between macrophages as senders (expression of ligand) and receivers (expression of receptor) and other cell types (Fig. 2b,c). To understand these interaction pairs not only on statistical level, but on the level of concrete ligand-receptor pairs, we performed the CellChat analysis (Jin et al, 2025). This tool quantitatively infers intercellular communication networks and detected particular ligand-receptor pairs including the communication probability (Fig. 2d). To better understand the role of macrophages in the dental epithelial stem cell niche, we further focused on the macrophage-dental epithelium clusters interactions. Here we found a specific Lgals9-Cd44 pair with high communication probability. The expression of CD44 was validated on the protein level in the progenitor region of dental epithelium (Juuri et al, 2012) and it co-localized with position of AIF1+ macrophages (Fig. 2e). Additionally, in the stellate reticulum of labial cervical loop (LaCL), we identified macrophages closely associated with previously discovered* $Fos^{CRERT2}/R26^{ZsGreen1}$*-lineage traced transient progenitors of outer enamel epithelium (Fig. 2f). Taken together, these findings suggest that macrophages might play a role in epithelial stem cell niche homeostasis."*

5. As specified below, the authors narrow their interpretation to developing molar and incisor organs as well as the effects of genetic and pharmacological alteration on pre-eruptive and eruptive phases. A major gap in analyses relates to the effects of CSF-1/r levels on the state of resident macrophages within alveolar bone, both at the base of growing roots and the bone that overlies a fully formed crown. Such an oversight leads to the conclusion that the biology of root formation/elongation as well as tooth eruption is not well leveraged or explored.

We appreciate the reviewer's feedback related to this and understand his/her concern. For this reason, we decided to characterize in more detail the role of macrophages and osteoclasts in a peri-eruptive stage (postnatal day 14) paying special attention at the different suggested regions: eruption space above the molar crown and the base of growing root. To address this, we have performed new set of immunofluorescence assays to detect AIF1 (macrophages) and Cathepsin K (osteoclasts) in postnatal day 14 jaws of *Wnt1*^{Cre}/*Csf1*$^{fl/fl}$ and matching-age wildtype mice. The images and quantifications related to the number of macrophages and pre- and osteoclasts in the area above the cusps and the base of growing roots are found in the updated version of **Figure 4**. As complementary data, we show as well how osteoclast activity is not arrested but delayed in this mouse model in the alveolar bone surrounding the molars (**Figure 4a,b**) and the incisor (**Figure 4f,g**). We explain these new results in the text as follows:

Page 8, lines 13-26: "*To further elucidate this phenotype, we conducted a detailed characterization of the jawbones in these animals (Fig. 4). Molar eruption and root formation were developmentally delayed (Fig. 4a,b). To quantify these defects, we measured the number of AIF1+, CTSK+ and AIF1+/CTSK+ positive cells in the eruption space and root growth space (Fig. 4c–e), as well as morphometric parameters including eruption distance and root length (Fig. 4f,g). Our results show decrease of AIF1+ cells (macrophages) in mutant mice, however no significant difference was observed in the number of CTSK+ (osteoclasts) or AIF1+/CTSK+ (pre-osteoclasts) cells between mutant and wildtype animals (Fig. 4d,e). Subsequently, we examined the alveolar bone socket of mouse incisor. In contrast to molars, the incisors of mutant mice exhibited a significant increase of AIF1+/CTSK+ cells (pre-osteoclasts) and in parallel a significant decrease of CTSK+ cells (osteoclasts) (Fig. 4h,i) along with a notably wider dental follicle space (haematoxylin-eosin images of Fig. 4h). These findings explain more prominent phenotype observed in incisors compared to molars (Fig. 3i,j) and suggest the important role of CSF1 signaling for proper differentiation of osteoclasts (Kim & Kim, 2016; Mun et al, 2020) (Fig. 4j).*"

The reviewer raises an important point regarding whether the observed dental phenotype reflects a tooth-specific effect or a secondary consequence of impaired bone formation and resorption resulting from disrupted Csf1 signalling. We agree that distinguishing between these possibilities is critical. Our data strongly support the interpretation that the observed phenotype is tooth-autonomous rather than bone-driven. In *Wnt1*^{Cre}/*Csf1*$^{fl/fl}$ mice, the alveolar bone develops with normal morphology, maintaining the overall jaw structure, which argues against a general bone formation defect (**Figure3i,j**). Additionally, the occurrence of supernumerary teeth adjacent to the first molar demonstrates that macrophage deficiency perturbs early tooth patterning directly, before bone formation takes place (**Figure 3j**). Finally, transient macrophage depletion in adult mice induces odontoblast and epithelial disorganization, indicating that macrophage clusters are intrinsically required for maintaining tooth cell integrity and function (**Figure 6h-k**). Collectively, our findings demonstrate that the dental abnormalities arise from direct effects on tooth development and maintenance, rather than as a secondary consequence of defective bone formation.

Of course, the alveolar bone surrounding the teeth should not be unnoticed and does not remain passive when affecting CSF-1 signalling. Our newly produced data presented in the new **Figure 4** shows that a delay in molar eruption and root formation happens because of affected migration and differentiation from monocytes to osteoclast.

Taking together, we believe that the supporting bone surely has some contribution to the observed tooth-phenotype but taking in consideration the above-mentioned findings we suggest that the lack of macrophages in the dental tissue is the primary cause of the described tooth problems.

6. Authors fail to provide insight into their findings of what roles resident or circulating macrophages may play in dental epithelium and the enamel organ, for example, the stellate reticulum and cervical loop area. While the phenotypic changes in dentition are striking, the manuscript can clearly benefit from a more thoughtful interpretation of these descriptive findings.

We appreciate the reviewer's insightful comment regarding the need for a clearer explanation of the specific role of macrophages within the epithelial compartment, both in the enamel organ of molars and in the cervical loop of the incisor.
To address this, we approached the question utilizing various methodologies:

1. **New computational single cell data analysis**: We conducted a more detailed analysis of the previously published single-cell RNA sequencing dataset (Krivanek et al., 2020). First, we introduced a heatmap showing the number of interactions among the various cell clusters identified in the tooth, including macrophages. In addition, we included circle plots to illustrate the role of macrophages as both signal senders and receivers (**Figure 2a-c**). To provide a more targeted insight, we performed a CellChat analysis, which allowed us to identify specific ligand-receptor pairs mediating interactions between macrophages and cells located in the most apical part of the incisor. These included interactions with the dental follicle, dental pulp, and epithelial compartment – **specifically the outer enamel epithelium, stellate reticulum, and stratum intermedium**. This new data is presented in the new version in the **Figure 2d**.
   We further validated these findings on tissue sections, demonstrating that macrophages are embedded within the epithelial compartment and are found in close proximity to **CD44+ epithelial stem cells from the stellate reticulum and stratum intermedium** (**Figure 2e**). Moreover, these results are consistent with the results presented in the first submission of our paper in which we used *Fos*^{CreERT2}/*R26*^{ZsGreen} tracing, **showing how macrophages are in direct physical contact with outer enamel epithelium progenitors**. This data can be found in **Figure 2f**.
   Taken together, these findings highlight the role of macrophages within the epithelial compartment as key modulators of stem cell maintenance and activity.

The main text was adapted as follows:

Page 6, lines 8-29: *"**Cellular and molecular interactions of macrophages with other cell types and stem cell niche**. In tooth, similar to other organs such as mammary gland or salivary gland, resident macrophages are in close contact with heterogenous cell populations as integral part of the microenvironment (Zhou et al, 2023; McKendrick et al, 2023). Based on previous single cell transcriptomics data (Krivanek et al, 2020) we delved into the interaction and cell*

communication between macrophages and the rest of cell clusters present in the mouse incisor. Although, macrophage cluster is not among the most interactive cell groups, a range of 25-50 interaction partners mainly with dental follicle, stellate reticulum (SR), stratum intermedium (SI) and outer enamel epithelium (OEE) was detected (Fig. 2a). The subsequent analysis of separate signals revealed the interaction probability between macrophages as senders (expression of ligand) and receivers (expression of receptor) and other cell types (Fig. 2b,c). To understand these interaction pairs not only on statistical level, but on the level of concrete ligand-receptor pairs, we performed the CellChat analysis (Jin et al, 2025). This tool quantitatively infers intercellular communication networks and detected particular ligand-receptor pairs including the communication probability (Fig. 2d). To better understand the role of macrophages in the dental epithelial stem cell niche, we further focused on the macrophage-dental epithelium clusters interactions. Here we found a specific Lgals9-Cd44 pair with high communication probability. The expression of CD44 was validated on the protein level in the progenitor region of dental epithelium (Juuri et al, 2012) and it co-localized with position of AIF1+ macrophages (Fig. 2e). Additionally, in the stellate reticulum of labial cervical loop (LaCL), we identified macrophages closely associated with previously discovered FosCRERT2/R26ZsGreen1-lineage traced transient progenitors of outer enamel epithelium (Fig. 2f). Taken together, these findings suggest that macrophages might play a role in epithelial stem cell niche homeostasis."

And discussed as follows:

Page 13, lines 27-34: *"The mechanisms by which macrophages can exert their influence may be either in providing support for proliferation (Liu et al, 2016), producing growth factors (such as VEGFs) (Lai et al, 2019), or regulation of inflammation (Watanabe et al). Ligand–receptor analysis identified the macrophage Lgals9 ligand and epithelial CD44 receptor as a putative communication axis, suggesting that macrophage–epithelium crosstalk may mediate the regulation of proliferation and differentiation. However, the precise mechanism by which macrophages influence surrounding epithelial or mesenchymal stem cell niches to ensure proper tooth development remains to be investigated in future studies."*

2. **New microCT scans**. To understand how the lack of macrophages result in disruption of epithelium layer and therefore impairs enamel matrix production, we segmented the enamel of *Csf1r KO* mutant mice vs. wildtype. This new data is currently found in the **Supplementary Figure 4h,i**. Our new quantification of enamel volumes exhibits a significant drop in *Csf1R KO* vs. wildtype animals.

The main text was adapted as follows:

Page 9, lines 11-12: *"... These epithelial abnormalities in both the continuously growing incisors and molars were associated with a marked reduction in enamel formation in mutant mice (Supplementary Fig. 4h,i)."*

3. **New immunohistochemistry stainings.** We characterized the morphological epithelial defects based on COL4 and CDH1 staining in *Wnt1*^{Cre}*/Csf1*$^{fl/fl}$, *Csf1r KO* and wildtype mice in the incisor and molars. We included these new data as **Figure 3k,l** and **Supplementary Figure 4a-g** and provide textual explanation as follows:

Page 8, lines 1-10*: "Beside the defects in hard matrices, the induced absence of macrophages triggered severe morphological abnormalities in both epithelial and mesenchymal compartments of the continuously growing incisors (Fig. 3k,l, Supplementary Fig. 4a,b). Histological analysis of apical tooth region uncovered various defects affecting both teeth and associated structures. This included (i) misshaped and smaller epithelial LaCL, (ii) increased vasculature in the neurovascular bundle region and dental follicle or (iii) discontinuation of pre-ameloblast and ameloblast layers resulting in ectopic folding of epithelial layer (Fig. 3k,l, Supplementary Fig. 4a,b). In the developing molars we observed increased distance between the residual enamel epithelium and the oral epithelium (Supplementary Fig. 4c, d). Our findings indicate that Wnt1^{Cre}/Csf1$^{fl/fl}$ mutant mice display delayed eruption, yet show no defects in epithelial formation or maintenance during the pre-eruptive stages."*

7. Figure panels should be attended to, in terms of **labeling** all relevant structures, correct cross-referencing in the text and alignment with the description provided in the legends. The latter will benefit from a more detailed explanation of what symbols like arrowheads and arrows signify.

We thank this valuable point. In our revised manuscript we include new detailed annotations in all the main (**Figure 1**, **Figure 2**, **Figure 3**, **Figure 4**, **Figure 5** and **Figure 6**) and supplementary figures (**Supplementary Figure 1**, **Supplementary Figure 3** and **Supplementary Figure 4**) which better describe different anatomical/histological structures, as well we highlighted by the use of coloured arrowheads or semi-transparent domains (**Figure 1, Supplementary Figure 1 and Supplementary Figure 3**) the regions of interest to emphasize the main focus. In addition, we corrected some references of the figures across the manuscript and implemented changes in figure legends to better match all the figures.

Specific suggestions (By Section):

Title: Should be revised to more accurately reflect the data and conclusions of these studies.

8. For example, embryonic macrophages can only be described as essential in tooth development if there is an arrest in morphogenesis as seen in the failure to progress from the bud to the cap stage.
9. The term 'tooth' is more universally accepted when compared to 'dental'.
10. Perhaps the authors will consider using 'development' rather than 'morphogenesis' as the former is more inclusive of enamel and dentin formation while morphogenesis typically refers to developmental stages that precede matrix deposition.
11. Unclear as to what the term 'self-renewal' refers to in the title. Is this a reference to data on the effect of the lack of CSF-1/r on incisal growth or the finding of a supernumerary molar? Perhaps the term 'homeostasis' may be preferred.

We agree with all the reviewer's suggestion. Based on them we have adapted the title of the manuscript to better reflect the main conclusions of our study.

The original title: *"Embryonic and postnatal macrophages are essential for dental morphogenesis and self-renewal"*,

The updated version: "***Embryonic and postnatal macrophages are necessary for proper tooth development and homeostasis***"

***Abstract**:* Modifications needed based on how the manuscript is shaped after revision and should also accurately reflect the limitations of these studies.

12. Mention of the use of mouse models of tooth development and eruption is needed as 'bone marrow transplantation in 'healthy adults' can be interpreted as for humans, at first read.

We thank this comment, and in order to clarify this statement we changed in the text as follows:

Page 2, line 6-7: *"...To investigate their role in development, we genetically disrupted macrophage migration using neural-crest specific Wnt1^{Cre}/Csf1$^{fl/fl}$ and general Csf1r knockout **mouse** models."*

Page 2, lines 10-11: *"...In addition, the short-term pharmacological depletion of macrophages in wildtype **adult mice** caused striking but temporary damage of dental tissues."*

13. Specifying that the genetic disruption models were used to study macrophage activation is not accurate as tracing studies were not performed.

We thank this comment. To address this in the abstract we changed it in the text as:

Page 2, lines 12-13: "*...Following treatment, macrophages rapidly repopulated dental tissue and the presence of M2 reparative state phenotype was observed by single-cell RNA-seq analysis.*"

*(**Previous version:** Following treatment, macrophages rapidly repopulated dental tissue and polarized from M0 to M2 reparative state, leading to tissue restoration.)*

14. The concluding statement that these data reveal a non-immune role of dental macrophages is inaccurate based on the present data sets. Clearly, the rise in AIF-1+ cells in pre-eruptive and eruptive phases may suggest a role of circulating monocytes/macrophages and the likely formation of osteoclasts in bone resorption, which implies a role within the inflammatory cytokine pathways.

We appreciate this comment, and we adapted the abstract as follows:

Page 2, line 15-17: "*...This previously unrecognized role of macrophages in teeth is reminiscent of their function in complex tissue regeneration, and requires future studies to dissect precise cellular and molecular mechanisms.*"

*(**Previous version:*** This non-immune role of dental macrophages is reminiscent of their function in complex tissue regeneration, and requires future studies to dissect precise cellular and molecular mechanisms.)

Along with this concern, we assessed the spatial distribution of macrophages (AIF1+) and osteoclasts (CTSK+) during pre-eruptive and eruptive phases of normal (wildtype) tooth development to

appreciate the role of osteoclasts during tooth eruption. These results we present as a new **Supplementary Figure 1** and commented on this in the main text as follows:

Page 5, lines 26-34: "…*Besides the dental papilla/pulp mesenchyme, the presence of AIF1+ cells was also observed in the close surroundings of the tooth, including the superior wall of the alveolar crypt (the space between the enamel organ and the oral epithelium) and the dental follicle at different time points (Fig. 1b–g; Supplementary Fig. 1a–c). The presence of AIF1+ cells was also detected during later root elongation process at P14 in the dental follicle root-growing space (Fig. 1g). AIF1+ cells as monocyte-lineage derivatives, share common lineage with osteoclasts. These cells, characterized by expression of Cathepsin K (CTSK) (Dai et al, 2020; Wilson et al, 2009) are essential for bone resorption allowing root elongation and tooth eruption (Fig. Supplementary 1d-f)."*

Figure legend, Page 36, lines 5-13: "***Supplementary Figure 1. Spatio-temporal distribution of AIF1- and CTSK-positive cells during molar development.*** *At pre-/perinatal stages (E15.5, E17.5, P0), macrophages are located not only in the dental pulp but also in the superior wall of the alveolar crypt above the enamel organ/cusps (eruption space), and along the dental follicle* ***(a-c)****. From postnatal stages (P0, P6, and P14) alveolar bone is being resorbed as indicated by the presence of CTSK-positive cells (white arrowheads) in the superior wall of alveolar crypt (allowing molar eruption), and in the bone around the root growing space from P6 (allowing root elongation)* ***(d-f)****. Scale bars = 100 µm, insets = 50 µm. Am. = ameloblasts, DF = dental follicle, DP = dental pulp, E = embryonic day, EO = enamel organ, Ep. = epithelium, Od. = odontoblasts, OE = oral epithelium, P = postnatal day, sAC = superior wall of alveolar crypt."*

Introduction*:* Revise to include literature that will better orient the readership to the role of macrophages in tooth development, eruption and homeostasis

15. Scaling back the background on macrophage biology by citing recent review/s will allow for the addition of other relevant background.

We appreciate the reviewer's comment and agree that adding more recent references on literature was needed. Firstly, to better orient the reader, we have added several recent reviews related to a function of macrophages as regulators of processes such as development, growth, repair, and tissue homeostasis. This information can be found specifically in:

Page 2, lines 20-22: "…*Macrophages regulate complex cellular dynamics of tissues during development, growth, repair, and homeostasis in all tissues, as reviewed by many research teams (Mass et al, 2023; Guan et al, 2025; Zhao et al, 2024; Theret et al, 2019; Sehgal et al, 2021; Wynn et al, 2013)".*

Following with this, in the rest of the introduction, we elaborated on the following main points related with macrophage biology: (1) Origin as monocyte-derivates, (2) Diversity of tissue-resident macrophages (liver, brain, lungs or skin), (3) Canonical functions (engulfing cellular debris, clearing apoptotic cells and regulating inflammatory response) as well as novel evidences of their role

maintaining stem cell niches in organs like guts, mammary gland or hematopoietic niche. All this information is commented in detail as follows:

Page 2, lines 22-35; page 3, lines 1-3: *"..They are migratory and phagocytic cells that can differentiate from monocytes circulating in the bloodstream and are an integral part of the immune system (Dash et al, 2024). They serve as a non-specific first-line defence against harmful pathogens and initiate adaptive immune response by presenting antigens (Kierdorf et al, 2015). Beyond their critical immune role, macrophages extravasate from the vascular system or derive from primitive macrophages and persist as tissue-resident macrophages in numerous tissues (Haldar & Murphy, 2014). Here they gain diverse functions specific for the populated tissue which includes e.g., Kupffer cells in liver, microglia in brain, osteoclasts in bone, alveolar macrophages in lungs, Hofbauer cells in placenta, Langerhans cells in skin and many others (Lendeckel et al, 2022; Mass et al, 2023). Generally, tissue-resident macrophages actively support the proper function and integrity of tissues by homeostatic surveillance. This includes engulfing cellular debris, clearing apoptotic and senescent cells, and regulating the inflammatory response (Lendeckel et al, 2022; Watanabe et al; Hatch et al, 2024). Importantly, there is increasing evidence that macrophages are also crucial for the maintenance of stem cell niches in various tissues, including intestines, muscles, mammary gland, the hematopoietic niche and others (Manneken & Currie, 2023a; Ratnayake et al, 2021a; Zhou et al, 2023; Kayvanjoo et al, 2024; Winkler et al, 2010). The role of macrophages in the stem cell niche of teeth remains a completely unexplored area."*

16. A brief overview of the presence of other inflammatory cells in a developing and adult dental pulp as well as of macrophages that are also present in the periodontal ligament will be useful to include.

Based on the reviewer´s suggestions we decided to give a broader view of other immune cells such as neutrophils, B- and T-cells in the mouse teeth, with special focus on the pulp and periodontal tissue. We adapted it in the main text as continues:

Page 30, lines 25-35: "… *Recent studies focused on independent classification of various dental cell types via methods of single-cell RNA-sequencing (scRNA-seq) have revealed a proportionally substantial amount of immune cells present in both human and mouse teeth with diverse expression profiles (Krivanek et al, 2020; Pagella et al, 2021). These include neutrophils, B- and T-cells, or macrophages in both dental pulp or periodontal space (Sharir et al, 2019; Krivanek et al, 2020; Takahashi et al, 2019; Chiba et al, 2020; Nagata et al, 2021; Hanson-Drury et al, 2023). Although, previously, the role of immune cells have primarily been studied in the context of inflammatory conditions as pulpitis or periodontitis, the above-mentioned scRNA-seq studies have worked with healthy dental tissues (Figueredo et al, 2019; Pohl et al, 2024). Beside the immune functions, emerging studies have shown macrophages to be key players in reparative dentinogenesis (Neves et al, 2020; Kadowaki et al, 2022) in both human and mouse teeth. In addition to macrophages, dendritic cells have been also shown to participate in immunosurveillance and antigen presentation during pulp inflammation (Chen et al, 2024; Pohl et al, 2024; Quispe-Salcedo & Ohshima, 2021)."*

17. Also important to orient the reader better to the theories of tooth eruption where positive pressure of an elongating root creates osteoclastic activation in the superior wall of the alveolar crypt and the

eruption pathway. Classic literature citations should be included along with excellent reviews by Gary Wise along with his work on CSF-1 and tooth eruption.

We appreciate the suggestion of the reviewer about incorporating in the introduction information of tooth eruption and its relation with osteoclastogenesis in dental follicle and root formation. Indeed, we agree this is an important issue to address to orient better the reader. Based on this and other comments we revised many parts of our manuscript and adding many new results (as described in a detail above). In the introduction we are adding/editing the following text to provide more detailed background about the tooth eruption a root elongation:

Page 3, lines 20-24: *"…In addition, studies using the rodent dentition have demonstrated the process of alveolar bone resorption, making it a traditional model for understanding tooth eruption (Wise et al, 2002; Wise & King, 2008). Tooth eruption is a temporally and spatially precisely regulated process that requires the active participation of a dental follicle, leading to properly controlled alveolar bone resorption, and subsequent alveolar bone formation at the base of the bony crypt (Wise, 2009)."*

Additionally, in the Introduction, we provide better background for our new results where we focus particularly on the critical regions involved in proper tooth eruption – namely the superior wall of the alveolar crypt and the dental follicle. This aspect is addressed in the text as follows:

Page 4, lines 20-24: *"…Our results show that macrophages first appear during early stages of tooth development – in the condensed dental mesenchyme. Postnatally, prior to eruption, macrophage numbers expand sharply within the dental pulp while the teeth are still developing. Accordingly, we investigated the temporal dynamics of macrophage and osteoclast localization in key regions essential for tooth eruption and root formation, namely the superior wall of the alveolar crypt and the dental follicle."*

18. Information on what is known about dendritic cells that also play a role in immunosurveillance and antigen-presentation during pulpal inflammation is important to conclude.

We thank the reviewer for pointing out the incomplete information about the role of dendritic cells in inflammation of dental pulp. In this manner, we added in the Introduction section a mention about dendritic cells providing a better understanding about the complexity of immune cells in the context of pulpal inflammation:

Page 3, lines 33-35: *"…In addition to macrophages, dendritic cells have been also shown to participate in immunosurveillance and antigen presentation during pulp inflammation (Chen et al, 2024; Pohl et al, 2024; Quispe-Salcedo & Ohshima, 2021)."*

19. Tooth morphology should be accurately referred to. For example, the hard outer shell of a tooth is not composed of dentin and enamel (2nd para). It is the anatomical crown that is covered by enamel while dentin that is akin to bone forms the bulk of a mature tooth (crown ad root).

We fully agree with this comment. In revised manuscript we provide a more accurate explanation of the tooth morphology. We adapted the Introduction as follows:

Page 3, lines 1-12: *"…Teeth are highly mineralized structures located at the beginning of most vertebrate digestive tracts and they vary in their form, replacement, and growth across species. Despite these differences, vertebrate teeth share (with some degree of variation) essentially two parts: (1) the crown composed primarily of two mineralized tissues, dentin and enamel, which are respectively formed by odontoblasts and ameloblasts; and (2) the root, which is comprised of dentin and cementum. These hard tissues surround and protect the highly vascularized and innervated dental pulp where dentin-forming odontoblasts are located (Virtej et al, 2013; Luukko & Kettunen, 2016). While the ameloblasts are lost during the eruption of the crown, the dental pulp allows teeth to function as a living organ capable of responding to stimuli from the external environment."*

20. Briefly explain the mechanism by which clodronate liposomes deplete macrophages as it can provide essential background for readers unfamiliar with it.

We thank the reviewer for this feedback. We have added an explanation of the mechanism of action of this drug in the main text and referred to the relevant literature. The revised text reads as follows:

Page 10, lines 7-11: *"…Clodronate liposomes (belonging to a larger group of bisphosphonates) are widely used drugs that specifically eliminate macrophages and some of their tissue derivatives in living organisms (Xia et al, 2024; Sears et al, 2022). This drug is engulfed by metabolically active phagocytic cells, such as macrophages, and converted into a toxic ATP analogue that inhibits mitochondrial translocase resulting in cell death (Frith et al, 1997; Lehenkari et al, 2002)."*

21. The concluding paragraph is too speculative and should be revised to accurately reflect the results of these studies.

We agree with the reviewer´s suggestion, due to this we adapted the concluding paragraph of Introduction accordingly:

Page 5, lines 1-5: *"…Taken together, our study reveals previously unrecognized role of macrophages during tooth formation and homeostasis. Our findings demonstrate that macrophages are essential for proper tooth development and, in parallel, are closely associated and interact with the epithelial stem cell niche in continuously growing teeth. Our results using a tooth as a model system, may provide novel insights into the cellular mechanisms governing the development, growth and regeneration of other tissues."*

Results and Figures: This section draws the most criticism and needs a major revision. Key concerns are highlighted as follows.

22. In the first series, the temporo-spatial profiles of macrophage distribution in molar and incisor organs are described using the marker of phagocytotic macrophages, AIF-1/IBA1. Details of this cytoplasmic protein that is expressed as important for macrophage function will be useful for the unfamiliar reader. It is also unclear whether AIF-1 is expressed by macrophages that are recruited as part of an inflammatory/immune response.

We thank the reviewer for their suggestion. To address the first part of this comment, we added a general information about AIF-1/IBA1 which reads as follows:

Page 5, lines 14-19: "…*To investigate this aspect, we detected AIF1 (allograft inflammatory factor 1, also known as IBA1) which is highly and specifically expressed in the monocytic lineage, including macrophages and microglia (Kishikawa et al, 2017). AIF1 is a calcium-binding protein that participates in several intracellular processes (phagocytosis, membrane ruffling and F-actin remodeling) as well as activation of macrophages upon inflammation (De Leon-Oliva et al, 2023).*"

Next, in our revised manuscript we provide a new comprehensive data about AIF1 as a general marker for macrophages obtained by different methodological approaches:

1. **Single cell transcriptomics.** We included a differential gene expression (DGE) analysis from mouse pulp incisor dataset, proving that *Aif1* is highly and very specifically expressed in the cluster of macrophages along different classic macrophage marker such as *F4/80*, *Csf1r*, *Mrc1*, *Cxcl16 etc.* This new data is included in the updated version of **Supplementary Figure 2d**. According to this data we adapted the text as follows:

Page 6, lines 36; Page 7, lines 1-4: "…*To confirm the identity of Csf1r+ cell cluster as macrophages we performed a differential gene expression (DGE) analysis which proving the co-expression of other well-established molecular identifiers of macrophages (e.g., F4/80, Aif1, Mrc1, Cxcl16 and others) (Supplementary Fig. 2d) (Wei et al, 2023; Abdelaziz et al, 2020; Kim et al, 2019).*"

2. **Immunohistochemistry**. We validated those results by co-localization of F4/80 and AIF1 antibody staining (immunohistochemistry) in control and Clodrosome-treated animals. This data is presented in the **Figure 6e** and we comment on these results in the results as follows:

Page 10, lines 22-26: "…Immunohistochemical and flow cytometric (FC) analyses confirmed partial depletion of macrophages following Clodrosome treatment, with a recovery of macrophage numbers after a one-week break of Clodrosome treatment (Fig. 6b–g). Validation was based on the detection of the surface macrophage marker F4/80 and the intracellular marker AIF1, which were found to co-localize within the same cell population (Fig. 6e; Supplementary Fig. 2d)."

23. Figure 1 panels need to be more thoroughly labeled and should capture the superior wall of the alveolar crypt, in low and high magnification views. This is a critical area as it is expected that circulating monocytes/macrophages will dominate along with osteoclasts whose resorptive activities become evident during the pre-eruptive phases (onset at PN Day 8 for molars and incisors).

We thank the reviewer for this comment and we agree that the mentioned area – superior wall of the alveolar crypt – is a critical region. Based on this suggestion we implemented several changes in our revised manuscript.

In the new **Figure 1a-g** we provide a detailed labelling depicting a progressive increase of macrophages (AIF1+) colonizing the dental pulp, superior wall of the alveolar crypt (**yellow arrowheads**) and around the dental follicle (**grey arrowheads**).

In parallel we introduced new labels for different histological structures. This can be found adequately stated in:

Figure legend, Page 24, lines 12-16: *"AB = alveolar bone, Am. = ameloblasts, DF = dental follicle, DP = dental papilla/pulp, E = embryonic day, EO = enamel organ, IK = initiation knot, LaCL = labial cervical loop, LiCL = lingual cervical loop, NVB = neurovascular bundle, Od. = odontoblasts, OE = oral epithelium, P = postnatal day, RGS = root growing space, sAC = superior wall of alveolar crypt."*

Additionally, we performed a new imaging and now showing both low (stages E15,5; E17,5; P0, P3, P6 and P14; presented in new version of **Figure 1b,g**) and high magnification views (stages P0 and P3; found in **Figure 1d',e'**).

Moreover, we provide a new low magnification + detailed images on the regions of Superior wall of alveolar crypt, Dental follicle, Interradicular area and Root growing space of E15,5; E17,5; P0; P6 and P14 in a newly produced **Supplementary Figure 1a-f**. We adapted the results as follows:

Page 5, lines 26-29: *"…Besides the dental papilla/pulp mesenchyme, the presence of AIF1+ cells was also observed in the close surroundings of the tooth, including the superior wall of the alveolar crypt (the space between the enamel organ and the oral epithelium) and the dental follicle at different time points (Fig. 1b–g; Supplementary Fig. 1a–c)."*

The new **Supplementary Fig. 1d-f** moreover shows close details of above-mentioned regions at P0, P6, and P14 – representative stages for the pre-eruptive and eruptive tooth development. This highlights not only the presence of macrophages via AIF1 staining but also presence of osteoclasts through combined AIF1 and CTSK staining. We explained in the text as follows:

Page 5, lines 31-34: *"…AIF1+ cells as monocyte-lineage derivatives, share common developmental history with osteoclasts. These cells, characterized by expression of Cathepsin K (CTSK) (Dai et al, 2020; Wilson et al, 2009) are essential for bone resorption allowing root elongation and tooth eruption (Fig. Supplementary 1d-f)."*

24. The presence of macrophages in the dental follicle and gingival mucosa seem to be ignored as is the rise in cell density that is visible within alveolar bone within the interradicular area of molar organs.

We appreciate this helpful comment from the reviewer. In the revised manuscript, we focused in detail on the presence of macrophages in the dental follicle, the superior wall of alveolar crypt, interradicular area and root growing space. Based on this comment we adapted the **Figure 1** accordingly (as described in detail in the previous question).

In the new version of **Figure 1**, we produced new images at lower magnification to include all these regions. We have also added yellow arrowheads to indicate the presence of AIF1+ cells in the superior wall of the alveolar crypt and grey arrowheads to show their distribution in the dental follicle. Due to space limitations in the main figure, a more detailed view is also included in **Supplementary Figure 1**. We comment on this in more detail in a previous question.

We edited results in the main text as follows:

Page 5, lines 26-29: *"…Besides the dental papilla/pulp mesenchyme, the presence of AIF1+ cells was also observed in the close surroundings of the tooth, including the superior wall of the alveolar crypt (the space between the enamel organ and the oral epithelium) and the dental follicle at different time points (Fig. 1b–g; Supplementary Fig. 1a–c)."*

Additionally, as suggested by the reviewer we include a new panel of staining in **Supplementary Figure 1d-f**, to highlight the presence of AIF1+ (macrophages) and CTSK (osteoclasts) cells in the forming-root space in molars and in the interradicular space once roots are in the process of elongating. We commented on these additional results accordingly as follows:

Page 5, lines 31-34: *"…AIF1+ cells as monocyte-lineage derivatives, share common lineage with osteoclasts. These cells, characterized by expression of Cathepsin K (CTSK) (Dai et al, 2020; Wilson et al, 2009) are essential for bone resorption allowing root elongation and tooth eruption (Fig. Supplementary 1d-f)."*

In order to improve the clarity of histological sections and the various structures depicted on them, we are now improving the labelling of many new areas: **Am. = ameloblasts, DF = dental follicle, LaCL = labial cervical loop, LiCL = lingual cervical loop, NVB = neurovascular bundle, Od. = odontoblasts, OE = oral epithelium, RGS = root growing space, sAC = superior wall of alveolar crypt.**

25. Page 5, line 18 - How did they identify which are Csf1 ligand and Csf1r receptor that are shown in supplementary Fig 1d?

We thank the reviewer for this comment. It may not have been clear in the previous version of this supplementary figure, staining using the *Csf1* or *Csf1r* probes (RNA Scope methodology) was performed independently on different slides. To improve the readability of our images we reorganized this figure significantly and present it as **Supplementary Figure 3**.

To ensure this point is more easily understood by readers, we have added **clearer labels** which are now positioned **next** to the images (not inside), indicating whether the **Csf1 (ligand)** or the **Csf1r (receptor)** was detected. In addition, we introduced **arrows** and **color-coded semi-transparent regions** to better illustrate the spatiotemporal distribution of both probes. Along with that we introduce detailed labels of various histological structures which includes: **Am. = ameloblasts, Cond. Mesen. = condensed mesenchyme, DF = dental follicle, EO = enamel organ, IK = initiation knot, DP = dental pulp, LaCL = labial cervical loop, LiCL = lingual cervical loop, Od. = odontoblasts, Mϕ = macrophages, pre-od = pre-odontoblasts, pre-am = pre-ameloblasts.**

This information has been incorporated into the main text as follows:

Page 7, lines 6-9: *"…The expression of the Csf1 in the dental tissue was not restricted to the adult stages only, but both Csf1 and Csf1r were present in the developing molars and incisors within dental papilla/pulp, dental follicle, odontoblast layer and inside of the dental epithelium (Supplementary Fig. 3)."*

26. In result, Page 4, line 33 and also in Page 5, line 3-7, Fig 1jand k is mentioned but these figures are not labeled or referred to in the legend.

We appreciate a detailed revision of our manuscript provided. In our revised manuscript, we fixed the labelling and referring of all the figures.

27. The specificity of immunostaining is not convincing as seen in Figure 1i where background staining of odontoblastic processes and (pre) dentin is visible.

We appreciate this comment from the reviewer regarding the specificity of the AIF1 antibody. Unfortunately, the problem with all immunostainings performed on tooth samples is that the (pre)dentin matrix is "sticky" for antibodies and often provide a false-positive signal in this region. This fact could be seen also in our data. The specificity of the used antibody was proved and published many times. To acknowledge this, we are adding specific comment and reference in the materials and methods as follows:

Page 18, line 20-22: *"AIF1 (Novus, NB100-1028; 1:500, previously shown as specific for macrophages (Van Broeckhoven et al, 2022))"*

Moreover, in our revised manuscript we implemented several changes to address this:

- We adjusted the signal of the background (global linear adjustment) to enhance signal quality.
- In the new version of **Figure 1j**, it is now more clearly visible that AIF1 staining is specific to macrophages, which are associated with odontoblasts, so the signal does not originate from non-specific staining of odontoblast processes.
- We increased the size of images and provided more detailed zoomed-in insets (**Figure 1i',i''**).

28. The area labeled in panel 1j and described in the text (part 3 of Results) is the collapsed intermediary layers of the enamel/dental organ and should be identified as such. It is curious that the morphology of the immunostained cells so closely resembles that of dendritic cells.

Thanks to this comment, we realized that in the first version of our manuscript there was an error in referencing this figure (it should have been **Figure 2j**). In the revised manuscript, the previous **Figure 2j** has been replaced by a new **Figure 2e**. This panel now shows not only the presence and distribution of macrophages in the labial cervical loop (together with **Figure 2f**) but also **new features of this epithelial structure – the expression of CD44 in the labial cervical loop**, which was predicted by our scRNA-seq analysis shown in **Figure 2d**.

To improve the clarity of this histological section, we added detailed annotations to the new **Figures 2e** and **2f**, including: LaCL = labial cervical loop, OEE = outer enamel epithelium, SI = stratum intermedium, SR = stellate reticulum.

Regarding the second part of the comment related to macrophages and dendritic cells. Macrophages and dendritic cells are closely related, and both come in multiple flavours, mature and immature, activated and non-activated. Also, they are highly similar in morphology when observed in tissues.

However, the formation of embryonic dendritic cells occurs late – around E17 in mouse embryo, and thus what we stain and characterize in embryos are mostly macrophages.

29. From this panel it is hard to delineate resident macrophages from those that are immunogenic and derived from circulating monocytes.

Although we agree with this comment, it was not our intention to clearly delineate this aspect here. Our goal in this panel was rather to provide a descriptive illustration of the presence and distribution of macrophages within the dental epithelial stem cell niche of the continuously growing mouse incisor.

In the revised manuscript, we now include new panels (**Figure 1j**) which illustrate the spatial distribution of macrophage populations across the whole apical part of mouse incisor. Specifically, we observe macrophages associated with blood vessels (yellow color-coded), likely representing recently extravasated monocytes that have just entered the tissue. Additionally, we identify distinct populations located within the dental follicle (cyan), along the odontoblast layer (green), and resident within the dental pulp (orange). The latter are characterized by co-localization with mKI67 (**Figure 1i,j**), suggesting *in situ* proliferation and a potential tissue-resident phenotype. We comment on these in the main text as follows:

Page 6, lines 2-7: *"…Co-staining of macrophages with mKI67, a marker of proliferating cells, revealed their proliferative capacity within the teeth, suggesting that these cells are pulp-resident and maintained through local proliferation. (Fig. 1i). The spatial distribution of macrophages in this tooth was not stochastic, but have shown an association with other cell types and histological structures such as neurovascular bundle, the odontoblast (and sub-odontoblast) layer, dental pulp cells or dental follicle region (Fig. 1j)"*

In parallel we show the new panel **Figure 2e** and the reworked panel **Figure 2f** with a detailed perspective of the labial cervical loop (as we describe in more detail within the previous question).

30. Figure 2a and 2b should identify the remaining cell clusters as it will be useful to know how the environment that macrophages are exposed to in the presence of Csf1 and also how its absence affects other cell populations.

We thank the reviewer for this helpful suggestion. In response, we have added the missing annotations for the remaining cell clusters, now shown in the new **Figure 3a,b**. Likewise, the annotations of single-cell clusters have been incorporated into the new **Figures 5a,b**.

31. The developmental stage of tissues used for scRNA-seq should be mentioned in the legend for panels 2a and 2b as well as for 2c and 2d.

We thank the reviewer for their valuable feedback. These referred data are now presented in **Figure 3a–d**, embedded in panels a and b, is cited that this dataset is obtained from (Krivanek et al.,2020) and we added in the figure legend that corresponds to adult mouse incisors. It is adapted as follows:

Page 27, lines 1-3: *"… **Figure 3. Wnt1^{Cre}/Csf1$^{fl/fl}$ induced depletion of macrophages and associated dramatic defects on adult dentition**. Cytokine Csf1 is present in both incisor growth and molar development, as predicted by scRNA-seq analysis from adult mouse incisor dataset (a-d)."*

For the panels of **Figure 3c,d,** we have indicated the developmental stage of the tissue in brackets embedded in the images.

32. As for Figure 1, it is essential for photomicrographic views to cover the superior, inferior and lateral walls of the alveolar crypt.

We agree on that. The **new Figure 1** and the **new Supplementary Figure 1** now fully cover this aspect (as we discuss in detail in previous comments). In the **Figure 3** we added aimed to show a detailed view on tooth itself and the specific region of the labial cervical loop of mouse incisor to illustrate the general depletion of macrophages in these specific regions in *Wnt1^{Cre}/Csf1$^{fl/fl}$* animals. Unfortunately, for adding more zoomed-out images we did not find enough space – also because we had to add several new panels into this figure. In appreciate also the regions of the superior, inferior and lateral walls of the alveolar crypt, we added detail annotations into panels **Figure 3c-h**.

New annotations added: **Dent. fol./DF = dental follicle, DP = dental pulp, EO = enamel organ, iAC = inferior wall of alveolar crypt, lAC = lateral wall of alveolar crypt, OE = oral epithelium, pre-am = pre-ameloblasts, pre-od = pre-odontoblasts, sAC = superior wall of alveolar crypt**

33. Figure 2 panels should be labeled accurately with developmental stages clearly marked.

The revised **Figure 3** (previous Figure 2) now include accurate labelling of various histological structure as well as developmental stages (as described in detail within the previous questions 31 + 32),

Moreover, we harmonized the labelling to be consistent through all new Figures and added the information about developmental stages into new **Figure 4**, **Figure 5**, **Figure 6a**, **Supplementary Figure 1**, **Supplementary Figure 3** and **Supplementary Figure 4.**

34. Page 6 line 7: the authors discuss developing molars, but the Figure refers to incisors. The stem cell niche referred to in the figure is only present in developing incisors not molars.

We apologize for this confusion caused by incorrect annotation of the original figure and text (as noted in question 28). This mistake has now been corrected, and references to individual panels have been updated.

35. Page 6 line 7-8, it should be Fig 2m also lines 10-15 are describing Fig 2 l and Fig 2m but is written as Fig. 2k
The same issue as above (question 34). Panels j,k (in previous Figure 2) were inserted in late stage, but references were not changed in the text. Now all is fixed in a new **Figure 3**.

36. Page 6, line 7 and Figure 2j,k – They mentioned that they observed developmental malformations in the developing molars (Fig. 2j, k). Maybe they meant incisors?

The same as above (question 34+35) – incorrect referencing in the text. Now is fixed.

37. Page 5 line 24-25 is confusing as the authors suddenly start talking about Fig. 3 while describing Figure 2.

We thank the reviewer for this helpful comment. In response, we reconsidered the position of this sentence to improve the flow of the text and to avoid referencing Figure 3 before the text for Figure 2 is complete. To address this, we have moved this sentence further down in the text to more appropriate position ensuring a smoother and more logical progression of the Results section, as outlined below:

Previous version (Page 5, lines 24–25): *"…Next, we examined tooth development in Csf1r KO, which lack macrophages in general (Fig. 3)."*

Revised version (Page 8, lines 29–30): "…*Next, we examined changes in tooth development by using a global Csf1-receptor knockout (Csf1r KO) animal (Fig. 5).*"

38. The presence of a supernumerary tooth placed mesial to the first mandibular molar is intriguing. How often was this observed? What were the total number of samples retrieved for analysis. Was this ever observed in the maxilla? If not, what hypothesis can be framed to explain regional differences in phenotype?

We thank this valuable feedback from the reviewer. Indeed, the phenotypic manifestations are variable. To cover the observed variability, we are now adding two new supplementary tables (**Supplementary Table 1** and **Supplementary Table 2**) where we summarize and quantify observed dental defects following the disruption of CSF signalling. **This table covers both mandible and maxilla, as well as incisors (length, folding, eruption, pulp reduction, bone-fusion, enamel) and molars (presence of roots, appearance of roots, patterning of cusps, supernumerary teeth, eruption status); different genotype, BMT and age.**

We comment on this in the text as follows:

Page 7, lines 27-36: "*MicroCT analysis revealed that the incisors of Wnt1^{Cre}/Csf1$^{fl/fl}$ animals exhibited an abnormal morphology, with generally reduced size, a hook-like shape and asymmetrical growth (Fig. 3i,j). Additionally, we observed local enamel defects and reduced dental pulp chamber filled with dentinous tissue (Fig. 3j). Interestingly, the apical part of affected incisor was shifted anteriorly, starting at the level of the first molar instead of its physiological posterior position close to the temporomandibular joint (Fig. 3j). In contrast to the incisors, the molars of Wnt1^{Cre}/Csf1$^{fl/fl}$ animals exhibited a less drastic phenotype, with mild morphological alterations of root and crown. Notably, in some of the Wnt1^{Cre}/Csf1$^{fl/fl}$ animals' supernumerary*

tooth was observed (Fig. 3j). Variability of all observed dental phenotypes of incisors and molars is summarized in detail in the Supplementary Table 1 and Supplementary Table 2."

Page 9, lines 34-25; Page 10, line 1: *"To reconcile the phenotypic variations observed between the conditional knockout (Wnt1^{Cre}/Csf1$^{fl/fl}$) and the global knockout (Csf1r KO), we summarized the findings for incisors and molars in Supplementary Table 1 and Supplementary Table 2, respectively."*

39. Figure 3 describes the effects of depleting Csf1r and the subsequent changes in molar and incisor organs. These appear strikingly similar to that seen with the conditional depletion of the Csf1 ligand. How can differences in the level of the ligand and receptor for Csf1 be reconciled?

We appreciate this comment from the reviewer. We addressed this comment in the "Discussion" section of the main text as follows:

Page 15, lines 18-23: "… *The phenotypic differences between Csf1R KO and Wnt1^{Cre}/Csf1$^{fl/fl}$ animals can be reconciled by the spatial and functional specificity of CSF1-CSF1R signalling. While global loss of CSF1R disrupts systemic macrophage development and homeostasis, neural crest-specific loss of CSF1 impairs local signalling required for craniofacial bone and tooth development, without affecting other CSF1-producing tissues. Moreover, residual CSF1 or compensatory ligands such as IL-34 may support survival and function of macrophages outside the neural crest-derived domains."*

40. Again, developmental stages are not specified in Figure 3 panels

Thanks to this comment, we paid more attention to this suggestion, and we decided to include in brackets developmental stage of the tissue in brackets. Either if its adult tissue (indicating the weeks-old), postnatal development (labelled as: P0, P3, P6, P14) or embryonic (labelled as: E12.5, E15, E17.5). Although this question referred to the previous version of Figure 3, we unified this criterion to the current version of the figures.

41. Page 6, line 27 should be Fig 3g instead of Fig 3i and Fig 3j

We apologize for the mismatch between the figures and the text in the previous version of the manuscript. Now is fixed.

42. With the BMT rescue experiments why are regional differences noted? Specific reference to earlier work of Wise et al., that documented how local administration of Csf1 corrected the failure to erupt should be included in the discussion

The effect of BMT within individual animals is indeed variable. Interestingly, similar to individual animals of used strains having the same genetic modification used in this study. To address this along with the reviewed manuscript we now include **two new tables** (**Supplementary Table 1** and

Supplementary Table 2), where we extensively studied and described several parameters of variability of phenotypes across different individuals. **This table covers both mandible and maxilla, as well as incisors (length, folding, eruption, pulp reduction, bone-fusion, enamel) and molars (presence of roots, appearance of roots, patterning of cusps, supernumerary teeth, eruption status); different genotype, BMT and age.**

We added this in the results as follows:
Page 7, lines 34-36: "*Variability of all observed dental phenotypes of incisors and molars is summarized in detail in the Supplementary Table 1 and Supplementary Table 2.*"

Page 9, lines 34-35; Page 10, line 1: "*To reconcile the phenotypic variations observed between the conditional knockout (Wnt1^{Cre}/Csf1$^{fl/fl}$) and the global knockout (Csf1r KO), we summarized the findings for incisors and molars in Supplementary Table 1 and Supplementary Table 2, respectively.*"

We thank this reviewer for suggesting the work Wise et al., which we now discuss where we show which part of the CSF1-based phenotype stems from the bone (new postnatal day 14 experiments and analysis of tooth roots – see **Figure 4a-g**) and which is tooth autonomous.

These results are included in the main text as follows:

Page 8, lines 13-20: "*To further elucidate this phenotype, we conducted a detailed characterization of the jawbones in these animals (Fig. 4). Molar eruption and root formation were developmentally delayed (Fig. 4a,b). To quantify these defects, we measured the number of AIF1+, CTSK+ and AIF1+/CTSK+ positive cells in the eruption space and root growth space (Fig. 4c-e), as well as morphometric parameters including eruption distance and root length (Fig. 4f,g). Our results show decrease of AIF1+ cells (macrophages) in mutant mice, however no significant difference was observed in the number of CTSK+ (osteoclasts) or AIF1+/CTSK+ (pre-osteoclasts) cells between mutant and wildtype animals (Fig. 4d,e).*"

Indeed, this was a very relevant advice given by the reviewer, as Wise and colleagues showed in a number of now cited publications that external addition of CSF1 improves the eruption of teeth and influences bone remodelling via osteoclastogenesis. This is certainly very relevant to our analysis of alveolar bone. For this reason, we introduced the following passage and references into the main text, where we discuss eruption situation in CSF1-depleted animals.

Page 14, lines 29-36: "*The delayed molar eruption observed in Wnt1^{Cre}/Csf1$^{fl/fl}$ mice aligns with previous studies demonstrating that **local administration of CSF-1 accelerates tooth eruption and modulates bone remodeling through osteoclastogenesis** (Cielinski et al, 1995; Wise, 2009). As CSF-1 plays a critical role in supporting the recruitment of mononuclear cells (osteoclast precursors) to the dental follicle prior to the onset of eruption (Wise et al, 2002, 2005). These findings emphasize the importance of CSF-1 signaling for the coordinated interaction between the dental follicle and the surrounding bone to achieve correct tooth shape.*"

43. Figures 4 and 5 describe results of a pharmacological intervention using a form of bisphosphonate drug to reduce/deplete macrophages, presumably the resident population of cells. It is unclear how

the rationale for the delivery regimen was derived. Several details related to the exact dosage are also missing.

Based on this and other comments we decided to repeat the pharmacological treatment experiment and include several new conditions and methods of phenotype analysis. The revised manuscript now presents a comprehensive experimental layout encompassing the following new aspects:

a) Different ages of animals (3 weeks and 6 weeks).
b) Recovery status one week after the last treatment.
c) Prolonged effects of the treatment.
d) Depletion status of macrophages assessed by quantitative flow cytometry (F4/80 and Gr-1, newly explained in the text) and IHC (AIF1).
e) Co-staining of AIF1 and F4/80 on tissue sections along with scRNA-seq co-expression of *Aif1*, *F4/80* and other known molecular markers of macrophages.
f) Cytokine activity in treated versus control animals.
g) Correlation between results of cytokine assay and scRNA-seq data from treated vs. control animals.
h) Improved clarification of distinct (sub)clusters of M1-like and M2-like macrophages following treatment compared to control animals.

The new **Figure 6a** now depicts the updated delivery regimen in a clear way and we adapted accordingly in the Materials and Methods section when the Clodronates were administered, as follows:

Page 19, lines 20-29: *"Animals were injected with an amount of 200 µl of the bisphosphonates drug per 30g of body weight. The animals underwent to different regime strategies based on the condition of study: (1) acute: 6 weeks-old mice received 3 consecutives daily i.p. injections (200 µl per 30g of body weight) and harvested one day after; (2) recovered: 6 weeks-old mice received 3 consecutives daily i.p. injections (200 µl per 30g of body weight) and harvested one week later; (3) prolonged; 6 weeks-old mice underwent a regime of 7 injections over 29 days, every two days until day 8 with 100 µl i.p and then, weekly with 100 µl for two weeks (day 15 and day 22). Exclusively, for single cell sequencing (10x), 3 weeks-old mice were used being i.p. injected for three consecutive days and collected on fourth (200 µl per 30 g body weight), for three consecutive days and collected on the fourth."*

44. Page 8 lines 3-4 should be Fig 4e instead of Fig 4c.

We apologize for the mismatch between the figures and the text in the previous version of the manuscript, and we fixed and checked this in the new version of the article.

45. Page 8, line 3 – Which treatment and which result depicts that physiological growth and tissue morphology were completely restored? In Fig 4, there is no mention about panel c in the figure legend.

The legend of the new **Figure 6** (previously Figure 4) has been completely restructured, and all panels are now properly referenced in the legend.

46. "Importantly, after the treatment was stopped, the physiological growth and tissue morphology were completely restored (Fig. 4c)". It is not clear which image shows the restored morphology. Also please mention how long after treatment stopped, the recovery was observed and whether this timeline is consistent across different specimens.

We thank the reviewer for this feedback. Based on this, we implemented several changes into this figure to improve its clarity:

1. We added **white arrowhead** into microCT image to indicate at the site of the dentin/enamel defect in treated condition.
2. We added **black arrowheads** into hematoxylin and eosin images (**Figure 6k'**) to emphasize the site of the dentin/enamel defect in treated condition.
3. We added **yellow arrowheads** to indicate the reverted (back-to-normal) state of dentin/enamel after cessation of bisphosphonate treatment (**Figure 6k**).
4. We enlarged the histological images showing the transient disruption of the odontoblast and ameloblast layers **Figure 6i,k**.

The restored region, where both dentin and enamel layers reappear without morphological defects, is now indicated with a **yellow arrowhead** in **Figure 6k**. Additionally, **we correlated the distance** of the lesion (highlighted by black arrowheads in the updated **Figure 6k'**) with previously published data on the incisor growth rate (Gonzalez Lopez et al., 2023). The distance of the lesion from the labial cervical loop showed an exact correlation with the known growth rate and with the duration of the "healing period" after Clodrosome treatment was discontinued. We have addressed this point in the main text as follows:

Page 11, lines 7–17: *"…Notably, transient effect of macrophages depletion was specifically observed after prolonged Clodrosome treatment followed by one week recovery time (Fig. 6k). The impacted area was located approximately in distance of 1.5 mm from the LaCL. This distance corresponds to a weekly growth rate of mouse incisor, which was previously shown to be around 1.61 mm in adult mice (Gonzalez Lopez et al, 2023). Following cessation of treatment (days 22–28), the physiological growth and tissue morphology were completely restored resulting in restoration of the odontoblast and ameloblasts layers morphology and the dentin and enamel formation (Fig. 6k). This suggests a reversible effect of macrophage depletion and remarkable buffering capacity of these cells in order to restore tissue homeostasis."*

47. It is unclear why general macrophage markers – F4/80 and GRI were used instead of AIF1. Conversely, why were these markers that characterize all cells in the monocyte/macrophage lineage not used in studies described in Figures 1, 2 and 3.

We appreciate the reviewer's comment. The rationale for using the F4/80 marker instead of AIF1 for flow cytometry (as used in Figures 1–4) is that F4/80 is a membrane-bound marker of macrophages, whereas AIF1 is an intracellular protein. Although both antibodies were tested for flow cytometry, only F4/80 antibody provided a specific signal.

To validate that AIF1 and F4/80 are co-expressed within the same cells (macrophages), we performed a new experiment and bioinformatic analysis:

(1) Immunohistochemistry. We performed co-staining of F4/80 + AIF1 which signal clearly co-localize in macrophages within dental pulp. These results are now included in the revised version of **Figure 6e** and discussed as follows:

Page 10, lines 24–26: *"Validation was based on the detection of the surface macrophage marker F4/80 and the intracellular marker AIF1, which were found to co-localize within the same cell population (Fig. 6e; Supplementary Fig. 2d)."*

(2) Single-cell transcriptomics. Our new analysis proved that macrophages cluster highly specifically express many previososly known molecular markers of macrophages including *F4/80*, *Aif1* or *Csf1r*, which we used as key markers throughout this study **Supplementary Figure 2d**. We comment on this in the text as follows:

Page 7, lines 36; Page 8, lines 1-4: *"To confirm the identity of Csf1r+ cell cluster as macrophages we performed a differential gene expression (DGE) analysis which proving the co-expression of other well-established molecular identifiers of macrophages (e.g., F4/80, Aif1, Mrc1, Cxcl16 and others) (Supplementary Fig. 2d) (Wei et al, 2023; Abdelaziz et al, 2020; Kim et al, 2019)."*

48. Was a power analysis performed to determine the number of samples in each treatment group? FACS analysis results should be evaluated statistically as differences in F4/80 and GR-1 positive cells do not appear to be significant.

Thanks for this suggestion. In the revised experiment, we used a total of 18 mice (biological replicates). All of these animals were age-matched (6 weeks of age) and equally sex-distributed. Animals were divided into three experimental groups:

a) **Acute** — 3 daily injections of Clodrosome, harvested on day 4 (n=6 mice: 3 males, 3 females)
b) **Recovered** — 3 daily injections of Clodrosome, harvested on day 10 (n=6 mice: 3 males, 3 females)
c) **Control** — No Clodrosome administered (n=6 mice: 3 males, 3 females)

The maxillary and mandibular incisors pulps were pooled in pairs, obtaining in this way three replicates covering better the variability.

These different conditions are introduced in the Results section, as follows:

Page 10, lines 14-21: *"To investigate the role of macrophages in maintaining incisor homeostasis, we implemented different Clodrosome delivery regimens, and examined the dental tissue adaptive response following macrophage depletion (Fig. 6a). Clodrosome was delivered at specific time intervals to understand macrophages contribution upon different conditions: (1) acute response where tissue was harvested one day after three consecutive days of administration, (2) recovered status where tissue was harvested one week after three consecutive days of administration, (3) long-term effect where tissue was harvested after pulsed administration every other day for a week, and maintained once a week for three more weeks (Fig. 6a)."*

As well as the specific delivery regime is summarized in the **Figure 6a** and in the Materials and Methods section of the text accordingly:

Page 19, lines 20-29: *"Animals were injected with an amount of 200 µl of the bisphosphonates drug per 30g of body weight. The animals underwent to different regime strategies based on the condition of study: (1) acute: 6 weeks-old mice received 3 consecutives daily i.p. injections (200 µl per 30g of body weight) and harvested one day after; (2) recovered: 6 weeks-old mice received 3 consecutives daily i.p. injections (200 µl per 30g of body weight) and harvested one week later; (3) prolonged; 6 weeks-old mice underwent a regime of 7 injections over 29 days, every two days until day 8 with 100 µl i.p and then, weekly with 100 µl for two weeks (day 15 and day 22). Exclusively, for single cell sequencing (10x), 3 weeks-old mice were used being i.p. injected for three consecutive days and collected on fourth (200 µl per 30 g body weight), for three consecutive days and collected on the fourth."*

To make it clearer for the reader, we included in the figure legend of **Figure 6** the number of animals and their sex used for each experiment (FACS, immunofluorescence or TRAP staining) as follows:

Page 32, lines 3-18: ***"****FACS quantification revealing a drop of F4/80+ (macrophages) cells* ***(c)*** *and Gr-1 (granulocytes)* ***(d)*** *in isolated mouse pulps in control, acute and recovered conditions (n= 3 datapoints represented by different symbols, each data point is obtained from the incisor pulps pooled from 2 mice [1 female, 1 male]). Co-localization of AIF1 and F4/80 antibodies show both are markers of dental pulp resident macrophages* ***(e, e´)****. Reduction of amount of AIF1+ cells after acute response to bisphosphonates delivery quantified from immunofluorescence sections occurs in dental pulp* ***(f)*** *and epithelial compartment* ***(g)*** *(n= 3 mice per experimental condition represented by different symbols, all males). Bisphosphonates disrupt macrophage homeostasis leads to impaired morphogenesis of dentin and enamel visible from microCT scans (white arrowhead) and hematoxylin and eosin (HE) staining (black arrowheads)* ***(h-k)*** *which is reverted when bisphosphonates are no longer administered (yellow arrowheads). Missing activity of TRAP+ osteoclasts in the lesion area on dentin/enamel interphase after prolonged bisphosphonates delivery indicates that defect is not an outcome of elevated osteoclasts activity in this region* ***(l,m)****. Recovered status after Clodrosome treatment triggers an increase osteoclast activity in the incisor alveolar bone socket* ***(n)*** *(n= 3 mice per experimental condition represented by different symbols, 2 females and 1 male)."*

The FACS analysis was performed again with more biological replicates and the drop of F4/80 macrophages was analysed and statistically evaluated (**Figure 6c,d**). Our results show significant drop of F4/80 macrophages after acute treatment and restoration into normal state after recovery period. We comment on this in the revised version of our manuscript as follows:

Page 10, lines 29-36 – Page 11, lines 1-2: *"In acute response experiment we detected a significant drop of amount of F4/80+ cells from 15.23 ± 0.25 % (Mean ± standard deviation) (untreated condition) to 4.97 ± 1.46 % in the acute condition. The ratio of F4/80+ macrophages was restored after recovery period to 17.5 ± 4.81 % one week after last Clodrosome delivery (Fig. 6c). Additionally, we analyzed the ratio of Gr-1+ cells (common marker of granulocytes, differentiating monocytes and macrophages) after the Clodrosome treatment. FC analysis showed a similar trend to that observed for F4/80 with ratio of Gr-1+ cells of 1,43 ± 0.35% in controls, 0.66% in acute-response and 1.66% in the recovered group (Fig. 6d). Likewise, quantification of AIF1 expression by immunohistochemistry on paraffin sections revealed a reduced number of AIF1+ cells in both the dental pulp (Fig. 6f) and the epithelial compartment of LaCL (Fig. 6g)."*

49. Several other questions persist about these experiments as it is confounding to find a localized defect in dentin and enamel that the authors claim are not related to osteoclastic resorption. Since bisphosphonates are known to suppress osteoclastic activities these results are confounding indeed. Furthermore, the authors may consider the phenomenon of internal resorption that is the result of resorptive cell activity that reside within dental pulp.

This is a very good point. We are aware that bisphosphonate treatment affects not only macrophages but also osteoclasts, and discuss it in the text as follows:

Page 11, lines 18-22: *"Clodronate is commonly used to treat diseases associated with improper bone metabolism whereby its mechanism of action is to affect osteoclasts, cells derived from macrophages that have the ability to resorb hard tissue. To exclude the association of observed dental lesions with ectopic activation of osteoclasts, we performed TRAP (Tartrate-resistant acid phosphatase) assay to detect the osteolytic activity of osteoclasts (Fig. 6l-n)."*

Keeping this in mind, we first excluded the potential involvement of osteoclasts in causing the isolated, regional and restorable morphological defects of enamel and dentin observed in the incisors (**Figure 6h–m**).

To further confirm this, we performed TRAP staining to assess osteolytic activity of osteoclasts in the affected region. This analysis revealed **neither the presence of osteoclasts nor any traces of residual osteoclastic activity in the affected area**, with only a few TRAP-positive cells observed in the adjacent bone, not the lesion itself. Osteoclasts in association with the alveolar bone of the continuously growing incisor are physiologically associated with permanent growth and associated periodontal tissue remodelling (**Figure 6m**). We commented on this particular aspect accordingly in the text as follows:

Page 11, lines 22-24: *"Osteoclasts in association with the alveolar bone of the continuously growing incisor are physiologically associated with permanent growth and associated periodontal tissue remodeling (Omi & Mishina, 2022)"*

Moreover, since it has been reported in literature that traces of tartrate-resistant acid phosphatase in the matrix may persist even after osteoclasts themselves have disappeared (Nakayama *et al*, 2011), we decided to not overlook at this point and comment in the main text, as follows:

Page 11, lines 24- 26: "*We detected a normal distribution of osteoclasts in this region and a complete absence of any TRAP+ cells or traces of TRAP staining (both indicating osteoclast activity) (Nakayama et al, 2011) at the site of the enamel and dentin damage (Fig. 6l,m).*"

However, in the region of tooth lesion caused by the prolonged administration of clodronates, neither TRAP+ cells nor matrix traces were detected.

Internal resorption is indeed an interesting phenomenon; however, no TRAP activity was detected in dental pulp cells or odontoblasts. Although we commented on this observation in the revised manuscript as follows:

Page 11, lines 13-17: *"This suggests a reversible effect of macrophage depletion and a remarkable buffering capacity of these cells to restore tissue homeostasis. Importantly, there is no evidence that other tooth-resident cells, such as dental pulp cells or odontoblasts, internalize or resorb Clodrosome, supporting the specificity of this approach for targeting macrophages within the tooth."*

To examine a potential reduction in TRAP+ cells after bisphosphonate treatment, we newly quantified TRAP+ cells and present these results in **Figure 6n**, which is discussed in the main text as follows:

Page 11, line 27-35: *"To further investigate this, we analyzed the dynamics of osteoclast activity in the jawbone following Clodrosome administration by assessing TRAP+ staining in the incisor alveolar bone socket. While acute treatment caused a slight but insignificant reduction of osteoclast activity, a significant increase was observed one-week after treatment cessation (Fig. 6n). This delayed response likely reflects a compensatory upregulation of osteoclast activity during the recovery phase."*

Altogether, our results bring strong evidence that osteoclasts are not the original cause of this localized defect.

50. Taken together, the concluding studies described in Figures 4 and 5 do not add much value to the manuscript as they are inadequately designed and poorly interpreted.

We believe that all the additional experiments which we performed based on comments of all three reviewers significantly improved the quality of the **Figure 6** and **Figure 7** of our revised manuscript. We discuss them in detail in several previous comments. Most importantly, we have performed an additional set of experiments to strengthen the conclusions and address the reviewer's concern regarding the design and interpretation of the studies previously shown in Figure 4 and Figure 5 (**now Figure 6 and Figure 7**).

These new experiments included analyses covering short-term (acute), long-term (recovery), and prolonged administration of Clodrosome. Our new data provides a comprehensive understanding of the effects of macrophage depletion over time and the process of recovery during tooth growth.

More specifically:

1) **Dynamics of macrophages depletion**: We now show the statistically relevant quantification of depletion of macrophages shortly after drug administration and their recovery one week after treatment – identified and quantified by both the flow cytometry (FC) shown in **Figure 6c,d** and immunohistochemistry (IHC) in **Figure 6e-g**. These results were commented accordingly in the main text, as continues:

Page 10, lines 22-24: *"Immunohistochemical and flow cytometric (FC) analyses confirmed partial depletion of macrophages following Clodrosome treatment, with a recovery of macrophage numbers after a one-week break of Clodrosome treatment (Fig. 6b–g)"*

Page 10, lines 29-36; Page 11, lines 1-2: *"In acute response experiment we detected a significant drop of amount of F4/80+ cells from 15.23 ± 0.25 % (Mean ± standard deviation) (untreated condition)*

to 4.97 ± 1.46 % in the acute condition. The ratio of F4/80+ macrophages was restored after recovery period to 17.5 ± 4.81 % one week after last Clodrosome delivery (Fig. 6c). Additionally, we analyzed the ratio of Gr-1+ cells (common marker of granulocytes, differentiating monocytes and macrophages) after the Clodrosome treatment. FC analysis showed a similar trend to that observed for F4/80 with ratio of Gr-1+ cells of 1,43 ± 0.35% in controls, 0.66% in acute-response and 1.66% in the recovered group (Fig. 6d). Likewise, quantification of AIF1 expression by immunohistochemistry on paraffin sections revealed a reduced number of AIF1+ cells in both the dental pulp (Fig. 6f) and the epithelial compartment of LaCL (Fig. 6g)."

2) **Osteoclastic activity:** Quantification of TRAP staining in the alveolar bone surrounding the incisor revealed that acute treatment caused a slight but insignificant reduction of osteoclast activity, while a significant increase was observed one-week after treatment cessation. This data is presented in the new **Figure 6l-n,** and commented in the text as follows:

Page 11, lines 18-35: "*Clodronate is commonly used to treat diseases associated with improper bone metabolism whereby its mechanism of action is to affect osteoclasts, cells derived from macrophages that have the ability to resorb hard tissue. To exclude the association of observed dental lesions with ectopic activation of osteoclasts, we performed TRAP (Tartrate-resistant acid phosphatase) assay to detect the osteolytic activity of osteoclasts (Fig. 6l-n). Osteoclasts in the adjacent alveolar bone with the continuously growing incisor are physiologically associated with permanent growth and tissue remodel (Omi & Mishina, 2022). We detected a normal distribution of osteoclasts in this bony region and a complete absence of any TRAP+ cells or matrix-traces of TRAP staining (both indicating osteoclast activity) (Nakayama et al, 2011) at the site of the enamel and dentin damage (Fig. 6l,m). Thus, the lesion is presumed to have been formed during dentin and enamel patterning and not secondarily. To further investigate this, we analyzed the dynamics of osteoclast activity in the jawbone following Clodrosome administration by assessing TRAP+ staining in the incisor alveolar bone socket. While acute treatment caused a slight but insignificant reduction of osteoclast activity, a significant increase was observed one-week after treatment cessation (Fig. 6n). This delayed response likely reflects a compensatory upregulation of osteoclast activity during the recovery phase."*

3) **Inflammatory response:** We analyzed cytokine activity in the dental pulp following bisphosphonate treatment. These data provide further insights into the molecular responses associated with macrophage depletion. This analysis and the quantification was performed using unbiased profile cytokine array kit and corelated with single-cell transcriptomics data obtained from Clodrosome-treated mice versus untreated condition. This data is presented in the new **Figure 6o,p,** and commented in the text as follows:

Page 12, lines 1-10: "*Bisphosphonate administration has been reported to influence the production of both pro- and anti-inflammatory cytokines, likely through apoptotic macrophage clearance and subsequent immune modulation. in the short term, bisphosphonates reduce pro-inflammatory cytokine production, whereas in the long term, this effect is reversed (Khanna et al, 2024). To investigate this phenomenon in our Clodrosome-treated mice, we employed two complementary approaches. First, pro- and anti-inflammatory cytokine levels were analyzed in isolated dental pulps of incisors using a targeted array profiling. This analysis revealed an overall downregulation of both cytokine classes (Fig. 6o). We then correlated these findings with scRNA-seq transcriptomic data from Clodrosome-treated mice, which similarly showed downregulation*

of key pro-inflammatory markers, including MIP1β, MIP1α, and TNFα (Fig. 6p), which was consistent with the cytokine assay on protein level."

The part focused on the scRNA-seq analysis between Clodrosome-treated versus untreated animals now is located in **Figure 7.** We included a new panel (**Figure 7e**) which includes a better characterization of the macrophage cluster based on the main markers used in our study (*Aif1*, *Csf1r*, *Mrc1* and *F4/80*). Moreover, we split the previous Figure 7f, and we created two independent panels one for M2-like associated markers (new **Figure 7g**) and other one for M1-like associated markers (new **Figure 7h**). This was adjusted accordingly in the main text as follows:

Page 12, lines 18-22: "*Finally, and most importantly, scRNA-seq analysis revealed a phenotype shift of macrophages in the macrophage cluster (Fig. 7e) from M0-like (resting) into M2-like (healing) after acute-Clodrosome treatment. This newly emerged macrophages subset (Fig. 7f) exhibited elevated expression of M2-like associated markers, including Stab1, C1qa genes rather than M1-like (Cxcl2 and Ccl4 genes) (Fig. 7g,h).*"

Together, these new results substantially improve the experimental design and interpretation, reinforcing the overall conclusions of the study.

51. Figure labeling is erroneous in multiple figure panels. This needs to be clarified for proper interpretation of findings.

We fixed the incorrect labels throughout the manuscript.

52. Did the authors notice any difference in spatial expression pattern of Csf1ligand and Csf1r receptor in dental epithelium vs dental mesenchyme (Supplementary Figure 1d)?

To spatially delineate the expression of *Csf1* and *Csf1r* during incisor and molar development we significantly revised this supplementary figure (now presented as a **Supplementary Figure 3**). This figure now better visualize spatio-temporal distribution of *Csf1* and *Csf1r* expression in the developing teeth from pre-natal (E12,5; E15;5; E17,5) to postnatal (P0; P14) stages. We **added annotation of spatial distribution** with **color-coded semi-transparent regions**, **arrowheads** pointing on positive regions and **new zoomed-in details** (**Supplementary Figure 3f**) on the presence of macrophages in progenitor area of incisor and pulp horn of the molar. To address the question about the epithelium and the mesenchyme distribution, we adapted the text as follows:

Page 7, lines 6-9: "*… The expression of the Csf1 in the dental tissue was not restricted to the adult stages only, but both Csf1 and Csf1r were present in the developing molars and incisors within dental papilla/pulp, dental follicle, odontoblast layer and inside of the dental epithelium (Supplementary Fig. 3).*"

53. Statistical results for the TRAP assay should be added along with the clarification of whether the observed absence of osteoclast activity aligns with prior studies on clodronate.

Based on this comment we analysed the amount of TRAP+ cells in the alveolar bone to provide relevant statistical results. The new quantification plot is displayed in **Figure 6n**. In the figure legend we state the number and sex of used animals so we adapted as continues:

Page 33, lines 13-18: *"… Missing activity of TRAP+ osteoclasts in the lesion area on dentin/enamel interphase after prolonged bisphosphonates delivery indicates that defect is not an outcome of elevated osteoclasts activity in this region **(l,m)**. Recovered status after Clodrosome treatment triggers an increase osteoclast activity in the incisor alveolar bone socket **(n)** (n= 3 mice per experimental condition represented by different symbols, 2 females and 1 male)."*

We introduced this in the Results section, as follows:

Page 11, lines 27-31: *"… To further investigate this, we analyzed the dynamics of osteoclast activity in the jawbone following Clodrosome administration by assessing TRAP+ staining in the incisor alveolar bone socket. While acute treatment caused a slight but insignificant reduction of osteoclast activity, a significant increase was observed one-week after treatment cessation (Fig. 6n)."*

As commented by the reviewer, we clarified that our observation with prior studies on clodronate conducted in animals. We adapted in the text as continues:

Page 11, lines 31-33: *"The absence of changes in osteoclast activity at short-term Clodrosome administration is consistent with previous studies showing that clodronates primarily inhibit osteoclast function over prolonged treatment rather than immediately (Pedrazzoni et al, 1995; Lin & O'Connor, 2017)."*

Discussion: A revision of this section is recommended and should be done by providing as many mechanistic insights as possible into the role of tissue-bound macrophages present in tooth organs and surrounding tissues. As written, the authors repeat what is already presented in the Results while leaving out many interesting facets of these studies.

We thank the reviewer for this valuable feedback. To enhance the clarity and depth of our Discussion, we have extensively revised this section, addressed all comments and discussed new findings. Although space limitations made this task challenging, we have done our best to provide a comprehensive yet concise revision.

54. What is the relevance of this research to other tissues (e.g., bone, cartilage, skin), and what are the potential applications?

We appreciate the reviewer's interest in the relevance of this research to other types of tissues.
In our revised manuscript we addressed this as follows:

Page 17, lines 8-17: *"…Such novel role of macrophages mirrors their involvement in complex tissue regeneration, for example during salamander limb regrowth or murine ear punch recovery (Simkin et al, 2024; Godwin et al, 2013). In these regenerative contexts, macrophages emerge as critical agents for developing and maintaining tissue integrity beyond mere inflammation, and their perturbation often results in distorted morphogenesis. Our results provide evidence for the*

essential role of macrophages in dental tissues and may also highlight their broader importance in other tissues, where they have been shown to regulate permanently active stem cell niches through interactions with myogenic progenitors and by contributing to the maintenance of stem cell environments in the intestine, mammary gland, and hematopoietic system (Manneken & Currie, 2023; Ratnayake et al, 2021; Zhou et al, 2023; Kayvanjoo et al, 2024; Winkler et al, 2010)."

55. The current study is primarily based on mouse models, which raises questions about its applicability to human teeth. Could you elaborate on any potential clinical implications or translational outlook?

We understand and appreciate this reviewer's concerns concerning the applicability of the presented findings to the human dentition. We elaborated more in this aspect in the main text as follows:

Page 17, lines 17-26: *"…Although our research was focused on mice, this model shares high degree of similarities in the tooth development and morphogenesis with human (Tucker & Fraser, 2014; Hulsey et al, 2020; Hermans et al, 2021; Cardoso-Moreira et al, 2020). Our findings suggest that macrophages may serve as potential therapeutic targets in disorders involving tooth malformations, including eruption failure and pulp stone formation. This hypothesis is further supported by the previously reported roles of macrophages in regulating stem cell niches (Manneken and Currie 2023). In addition, specific stimulation of macrophages in the dental pulp might be used to improve the formation of tertiary dentine after tooth injury. This hypothesis is based on previously described function of macrophages in mediating the healing of skin lesions (extensively reviewed by Sim et al. (2022) and their role in activating quiescent stem cells after skeletal muscle injury (Ratnayake et al, 2021)."*

56. The paper discusses the transition of macrophages from the M0 (quiescent state) to the M2 (repair state), but the role of M1 (pro-inflammatory state) is not explored in detail. Could this be addressed further?

We thank the reviewer for this insightful comment. In our revised manuscript, we have revised our figure focused on scRNA-seq analysis (new **Figure 7**) and added the M1 (pro-inflammatory) and M2 (repair-associated) molecular characteristics of macrophages identified in our analysis (control versus Clodrosome-treated animals). These results, provide a holistic and unbiased perspective on population structure following the treatment. The data are now presented in separate panels with headings distinguishing M2-like – associated gene markers (*Stab1, C1qa*) from M1-like – associated gene markers (*Cxcl2, Ccl4*) within the macrophage cluster (**Figure 7g,h**). We further emphasized this by outlining with a dotted line the emerging macrophage population characterized by an inflammatory signature (M2-like macrophages). These modifications have been appropriately incorporated into the main text as follows:

Page 12, lines 18-24: *"Finally, and most importantly, scRNA-seq analysis revealed a phenotype shift of macrophages in the macrophage cluster (Fig. 7e) from M0-like (resting) into M2-like (healing) after acute-Clodrosome treatment. This newly emerged macrophages subset (Fig. 7f) exhibited elevated expression of M2-like associated markers, including Stab1, C1qa genes rather than M1-like (Cxcl2 and Ccl4 genes) (Fig. 7g,h). These findings indicate rapid adaptive response abilities of macrophages, which instantly react to dental tissue damage and facilitate a fast recovery of*

homeostasis, thereby restoring function of the incisor — an organ critical for the survival of this species."

Moreover, this is polarity change from M0 to M2 is further addressed in the discussion, where this phenomenon is discussed beside the dental tissue as follows:

Page 16, lines 7-14: "…scRNAseq results from Clodrosome-treated animals vs. controls reveals not only the presence of a macrophage population but, more importantly, a phenotype shift from M0-resting macrophages to M2-healing macrophages. The rapid acquisition of M2-like macrophage phenotype in response to tissue damage aligns with previous studies showing that neural cells (Ji *et al*, 2020), human-derived gingival mesenchymal cells (Zhang *et al*, 2010), and dental pulp stem cells (Yang *et al*, 2023) promote M2 macrophage polarization to enhance wound healing. In summary, our findings suggest that after injury, the population of tissue-resident macrophages rapidly recovers and activates an M2 healing phenotype which leads to the swift restoration of a healthy tooth morphogenesis."

Animals: It is important to include the ratio of male to female embryos, young adult and adult mice used in these studies. Numbers of animals used in each group for each approach also need to be added.

We thank for this comment and we agree that stating clearly number of animals as well as the sex of the animal will make the study clearer and more transparent.

For adult animals, we have included the number of animals (n), as well as the ratio of males:females used for each study in the figure captions for each panel.

Regarding embryos, it is very unusual to assess (genotype) them to determine sex for experiments related to tooth development. Unfortunately, most of the embryonic tissue was collected prior to these revisions and we do not have any records (neither left samples) to determine the sex of animals used in this study. Given that, embryos were collected from the same litter, we can reasonably assume a 1:1 sex ratio. Since we did not know the sex of used embryos, we treated them the same and did not observe any signs of potential sex-dependent phenotype in our results. In parallel, we did not find any evidence in literature related to sex-differences in bone or tooth embryonic development (Lesciotto et. al 2022).

Methods:

57. Regarding the use of bisphosphonate drugs as a means to study pharmacologic macrophage depletion: Given the mixed therapeutic targets of bisphosphonates, it is difficult to determine that the drugs are directly linked to macrophage function in this system. Thus, it may be a stretch to attribute the transient phenotype observed after injection of these drugs to the 'depletion of macrophages' around the teeth. Even though TRAP staining was performed, proving the existence of some osteoclasts (but not necessarily their functional state), it would be more useful to study multiple other bone cells (osteoblasts, osteocytes) over a time course (pre-treatment, immediate post-treatment, x-days post treatment, etc.) alongside reliable markers of macrophages. This would allow one to better decipher the direct cellular link to this phenotypic change post treatment.

We appreciate this comment. We are fully aware of pleiotropic effect of bisphosphonates. We do not claim this treatment is absolutely specific. The Clodrosome experiment was designed to get a parallel research line – to study a transient effect – alongside our genetically-modified organisms, especially given that Clodrosome effectively depleted macrophages.

We are afraid that studying the osteoblast/osteocytes over a time course, as suggested here, would not bring any new understanding for the observed tooth-isolated transient effect, as we do not observe the defects in the surrounding bone at the level of bone morphology/density. The focus of our study are teeth, and we can detect the Clodrosome-driven effects in enamel and dentin, whereas the bone stays phenotypically silent during the treatment period (at the level of our investigation and timeframes).

Importantly, **the dental defects resemble those observed in CSF1 depletion – an orthogonal approach, and they are reversible in teeth after the stop of Clodrosome treatment.** Together, this brings further investigation of the bone away from the teeth – a central focus of this manuscript.

Also, the longer effects of bisphosphonate on the alveolar bone were addressed previously and are published by Wise and colleagues for the eruption stages (where indeed the bone remodeling is key). However, in our case, the Clodrosome was administered long after the tooth eruption occurs, in a stable situation for the bone and in a short-term mode. This explains that we do not see the effects in the bone next to the teeth, and therefore we do not follow this any longer.

We adapted accordingly this part of the Discussion:

Page 15, lines 25-30: *"Although bisphosphonates have been proposed as a tool for studying the osteoclast-mediated events of tooth eruption, as delays the molar eruption in rats (Grier & Wise, 1998), in our strategy Clodrosome was administered long after the tooth eruption occurs, in a stable situation for the bone for a short-term. This explains that we do not see the effects in the bone next to the teeth, and therefore we do not follow this any longer, focusing primarily on the tooth defects instead of bone."*

58. Authors have mentioned that Clodrosome treatment led to severe aberrations in enamel and dentin morphology-please clarify specific aberrations such as changes in thickness, mineralization, or cellular organization. Include quantitative data.

This is a very good point from the reviewer. Although the observed aberrations of dentin/enamel structure were clear, **the effect was only local and compared to the whole volume of dentin/enamel minor and, most importantly it was transient and fully restored after the end of the treatment without issues in alveolar bone.** Such effects included loss of the continuity of the enamel layer, formation of enamel folds of different morphology, and also the local losses of integrity of the dental layer – creating fractured deposited dentin specifically in incisors.

This is included in new version of **Figure 6h-k**, and we provide a better description of such reversible defects in the main text as follows:

Page 11, lines 3-13: *"…To assess the effect of transient macrophage depletion on the structure of dental mineralized tissues, we performed microCT analysis. The results revealed a transient disruption*

of dentin and enamel (Fig. 6h,j) ,further visualized in detail at the histological level (Fig. 6j,k) by discontinued odontoblast and ameloblast layers creating a anisotropically deposited dentin specifically in incisor. Notably, transient effect of macrophages depletion was specifically observed after prolonged Clodrosome treatment followed by one week recovery time (Fig. 6k). The impacted area was located approximately in distance of 1.5 mm from the LaCL. This distance corresponds to a weekly growth rate of mouse incisor, which was previously shown to be around 1.61 mm in adult mice (Gonzalez Lopez et al, 2023). Following cessation of treatment (days 22–28), the physiological growth and tissue morphology were completely restored resulting in restoration of the odontoblast and ameloblasts layers morphology and the dentin and enamel formation (Fig. 6k)."

Importantly, those similar defects (or any defects at all) were not observed in molars, which are stable and do not undergo continuous self-renewal like murine incisors. This indicates that the Clodrosome–macrophage–driven effects occur only when the dynamics of hard tissue formation are rapid and stem cell niches are active. Consistently, as noted above, no effects were detected in the bone during this short-term treatment.

We mentioned this point in the revised discussion of the manuscript, as continues:

Page 16, lines 1-4: *"These specific and transient defects in tooth were exclusively found in incisors and not in molars, since molars are stable and do not self-renew. This highlights that Clodrosome-macrophage-driven defects arose only when cell dynamics of hard matrix generation is fast, and the stem cell niches (epithelial and mesenchymal) are active."*

Reviewer #2 (Remarks to the Author):

In this study, Lopez et al. sought to study the non-immune role of macrophages in dental development. Using growing teeth of a few mouse models as study models, the authors found that macrophages are associated with both epithelial and mesenchymal stem niches throughout lifetime. The mouse models used in this study include Wnt1cre; Csf1 fl/fl, Csf1R KO and Clodronate-treated mouse as the pharmacological depletion of macrophages model. The teeth of these animals exhibit abnormal dentin and enamel deposition, eruption defects and tooth mispatterning. The phenotypes in the Csf1R KO can be partially rescued by bone marrow transplantation. The depletion of macrophages by clodronate causes striking but temporary defects to the dental tissues. From these results, the authors propose that macrophages have essential roles in dental development and patterning of both epithelial and mesenchymal parts of the tooth.

I found this study is highly comprehensive and significant. The concept of the non-immune role in tooth development is extremely novel. Additionally, the manuscript is well-written. However, there are some points regarding the study's rigor that I would like to point out as listed below;

Thank you very much for these kind words and for the valuable feedback, which we greatly appreciate. Based on the provided comments (together with comments from other reviewers), we have carried out numerous substantial changes of our manuscript. Below, we provide a detailed, point-by-point response outlining all the relevant changes made.

Major points:

1. Overall, all the animal models used in this study seem to have significant limitations to target macrophage cell population only directly and specifically. The evidence shown in this manuscript showing that targeted cells are specifically macrophages are not particularly strong and need further experiments to support the conclusion.

We thank the reviewer for this comment and fully understand this concern. We agree that specifically targeting macrophages is a crucial first step in our study to draw any solid conclusions.

In the revised manuscript, we place greater emphasis and provide stronger evidence that macrophages are the primary targeted cells in our experimental models (wild-type, genetically modified, and Clodrosome-treated animals). To support this conclusion, we have included new supporting data obtained using several complementary methodological approaches:

1. **Single-cell transcriptomics data.** We newly performed differential gene expression (DGE) analysis using healthy mouse incisor dataset. This analysis provided quantitative expression data and revealed that *Csf1r* is along with other macrophage markers used in our study – such as *Aif1* and *F4/80* strongly and highly specifically co-expressed in the macrophage cluster. (**Supplementary Figure 2d**).

   These new data confirm that *Csf1r+* macrophages in the tooth serves as a primary target for general Csf1r KO depletion as well as for targeted depletion of CSF1 ligand (having affinity to CSF1R receptor, using *Wnt1*^{Cre}/*Csf1*$^{fl/fl}$ animals) (**Supplementary Figure 2b-d**). Thus, for all other dental cell types, CSF1 acts as an "invisible" factor and is not expected to affect their function.

We commented on this in the main text in results results as follows:

Page 6, lines 32-36; Page 7, lines 1-6 : *"The Csf1/Csf1r system is central to macrophage survival, proliferation, differentiation, and migration (chemotaxis) in various organs (Stanley & Chitu, 2014; Sehgal et al, 2018). By investigating potential homing factors of macrophages using previously published scRNA-seq data, we noted that cell cluster previously identified as macrophages express colony stimulating factor 1 receptor (Csf1r) (Krivanek et al, 2020; Jones & Ricardo, 2013; Ushach & Zlotnik, 2016) (Supplementary Fig. 2a-c). To confirm the identity of Csf1r+ cell cluster as macrophages we performed a differential gene expression (DGE) analysis which proving the co-expression of other well-established molecular identifiers of macrophages (e.g., F4/80, Aif1, Mrc1, Cxcl16 and others) (Supplementary Fig. 2d) (Wei et al, 2023; Abdelaziz et al, 2020; Kim et al, 2019). In parallel, the expression of the corresponding Csf1 ligand in dental pulp cells suggested that the presence of macrophages in this region can be controlled by CSF1-CSF1R interaction axis (Supplementary Fig. 2a-c)."*

We also adapted the text in the Discussion to clarify this aspect:

Page 15, lines 8-10: "We showed that *Csf1r* can be used as a specific and reliable marker in dental tissue by single cell transcriptomics data and validated by RNA in situ hybridization, and is critical."

2. **Immunohistochemistry (IHC).** We further validated the scRNA-seq expression data of selected marker genes (AIF1 and F4/80) at protein-level in the tissue by immunohistochemistry. This co-staining revealed perfect co-localization of these used and known marker genes in the same cell type (macrophages) while AIF1 localized intracellularly and F4/80 on the cell surface. These results further confirm the identity of AIF1+ cells as F4/80-positive macrophages. Together with the scRNA-seq data these results confirm that Csf1r is expressed specifically in these double-positive AIF1+ and F4/80+ cells. We show this in a new **Figure 6e,e´** and comment on this in the text accordingly:

Page 10, lines 24-26: *"Validation was based on the detection of the surface macrophage marker F4/80 and the intracellular marker AIF1, which were found to co-localize within the same cell population (Fig. 6e; Supplementary Fig. 2d)."*

3. **RNA *in situ* hybridization.** We introduce a new version of **Figure 5c,d** where we better annotate all the histological parts of the section through continuously growing incisor and developing molar, showing the distribution of *Csf1r* (receptor expressed only in macrophages in the tooth) expression. The *Csf1r+* cells exhibit the same distribution in the dental tissue AIF1+ and F4/80+ macrophages. This is newly shown in the **Figure 1j.** Specifically, we observe macrophages associated with blood vessels (pink), within the dental follicle (cyan), along the odontoblast layer (green), and resident within the dental pulp (orange). The latter are characterized by co-localization with mKI67 **(Figure 1i,j)**, suggesting *in situ* proliferation and a potential tissue-resident phenotype. We comment on these in the main text as follows:

Page 6, lines 1-7: *"… Similar to the molars, macrophages populated the dental pulp of the continuously growing mouse incisor (Fig 1i-j). Co-staining of macrophages with mKI67, a marker of proliferating cells, revealed their proliferative capacity within the teeth, suggesting that these cells are pulp-resident and maintained through local proliferation. (Fig. 1i). The spatial distribution of macrophages in this tooth was not stochastic, but have shown an association with other cell types and histological structures such as neurovascular bundle, the odontoblast (and sub-odontoblast) layer, dental pulp cells or dental follicle region (Fig. 1j)."*

Additionally, we revised the **Supplementary Figure 3** which now better visualize spatio-temporal distribution of *Csf1* and *Csf1r* in the dental tissue from pre-natal to postnatal stages with emphasis on annotation of spatial distribution with color-coded semi-transparent regions, arrowheads pointing on the positive region and new details on the presence of macrophages in progenitor area of incisor and pulp horn the molar at P0 and P14 developmental stages. The Results were changed accordingly:

Page 7, lines 6-9: *"… The expression of the Csf1 in the dental tissue was not restricted to the adult stages only, but both Csf1 and Csf1r were present in the developing molars and incisors within dental papilla/pulp, dental follicle, odontoblast layer and inside of the dental epithelium (Supplementary Fig. 3)."*

To ensure this point is more easily understood by readers, we have added **clearer labels** which are now positioned **next** to the images (not inside), indicating whether the **Csf1 (ligand)** or the **Csf1r (receptor)** was detected. In addition, we introduced **arrows** and **color-coded semi-transparent regions** to better illustrate the spatiotemporal distribution of both probes. Along with that we introduce detailed labels

of various histological structures which includes: **Am. = ameloblasts, Cond. Mesen. = condensed mesenchyme, DF = dental follicle, EO = enamel organ, IK = initiation knot, DP = dental pulp, LaCL = labial cervical loop, LiCL = lingual cervical loop, Od. = odontoblasts, Mϕ = macrophages, pre-od = pre-odontoblasts, pre-am = pre-ameloblasts.**

4. **Genetic macrophage-depletion strategies.** We utilize two mouse models to investigate the role of macrophages in tooth. Firstly, we used a neural crest-specific *Wnt1^{Cre}/Csf1$^{fl/fl}$* strain to disrupt the Csf1-ligand based signalling in neural crest derivates (all mesenchymal parts of the tooth). Thus *Csf1r+* macrophages which are responsive to this ligand fail to populate the tooth (**Supplementary Fig. 3e**). Second approach use the global knockout for *Csf1r* (*Csf1r KO*). In both scenarios, we detected none or very little of AIF1+ cells (**Figure 3e-h**, **Figure 5f**) in the tissue compared to wildtype controls. We commented on this aspect in the main text as continues:

Page 7, lines 18-21: *"In mutant Wnt1^{Cre}/Csf1$^{fl/fl}$ mice, AIF1+ macrophages are almost absent in incisors and molars compared to wildtype animals (Fig. 3e-h). Interestingly, although the conditional deletion of Csf1 by Wnt1^{Cre}/Csf1$^{fl/fl}$ was targeted to the mesenchymal part of the tooth, macrophages were not observed neither in the epithelial compartment of growing teeth (Fig. 3g)."*

Interestingly, our bone marrow transplantation (BMT) experiments in *Csf1r KO* showed that the phenotype rescue of the dental phenotype happens when macrophages derived from transplanted bone marrow re-inhabit the teeth, proved by co-staining of GFP+/AIF1+ cells and **new quantifications** data presented in **Figure 5f-h**. Newly commented in the text:

Page 9, lines 18-23: *"To assess the success rate of BMT in relation to dental tissue, we quantified the engraftment of the transplanted cells by IHC co-staining of AIF1 and GFP. Although in the dental tissue of Csf1r KO animals we did not observe any AIF1+/GFP+ cells, after BMT the number of AIF1+/GFP+ cells increased to 52.15 cells per region of interest (ROI) in the dental pulp. This demonstrated the efficient infiltration and establishment of transplanted cells within the dental pulp and their contribution in the phenotype rescue (Fig. 5h)."*

In this revised version of the manuscript, we included a more in-depth analysis of the morphometric features of teeth and bone improved after BMT. We are adding 3D images obtained by microCT of the lower and upper jaws from different angles (**Figure 5l,m**) and quantifications of teeth and bone sizes (**Figure 5n,r)**. We commented this results in the text accordingly:

Page 9, lines 24-33: *"Further analysis by microCT demonstrated partial dental and bone phenotype rescue by 14 days (Fig. 5l,m), which included recovery of molars and incisors eruption and a general restoration of mandibular and maxillary bone thickness in BMT compared Csf1r KO animals . Furthermore, to assess morphometric features of wildtype versus Csf1r KO and Csf1r KO + BMT animals, we measured the mandibular, molar and incisor lengths (Fig. 5n-r). Although the overall mandibular length remained unchanged among the three groups (Fig. 5o) the incisor length showed a slight but significant increase following BMT (Fig. 5p). Notably, the first maxillary and mandibular molars displayed a significant and more pronounced increase in length in Csf1r KO + BMT mice compared to Csf1r KO controls (Fig. 5q,r). Data obtained from a*

phenotype rescue experiment thus show that BMT-derived cells promptly reach the dental tissues and provide in situ support to save a phenotype of malformed teeth."

Therefore, we conclude that the observed rescued phenotype is macrophages-dependent and does not arise from other cell sources being potentially intrinsically affected in our experiments.

5. **Pharmacological depletion of macrophages.** This approach relies on using known macrophage-depleting agent clodronate (bisphosphonates) previously proven to deplete these cells *in vivo*. Newly, we provide quantifications of the macrophages depletion efficiency in tooth utilizing two strategies:

    (1) Flow cytometry (FC). Quantification of F4/80+ cells (macrophages) showed a significant decrease in the number of F4/80+ cells following Clodrosome administration. Number of macrophages was restored one week after treatment cessation (**Figure 6c**). Thus, used drug has a transient effect: macrophages that engulf the Clodrosome undergo apoptosis, but once treatment stops, new population of macrophages promptly repopulate the dental pulp.

We commented this in the text as follows:
Page 10, lines 29-33: "*In acute response experiment we detected a significant drop of amount of F4/80+ cells from 15.23 ± 0.25 % (Mean ± standard deviation) (untreated condition) to 4.97 ± 1.46 % in the acute condition. The ratio of F4/80+ macrophages was restored after recovery period to 17.5 ± 4.81 % one week after last Clodrosome delivery (Fig. 6c)."*

    (2) Immunohistochemistry. This approach revealed a significant drop in number of AIF1+ macrophages affecting to both dental pulp (mesenchyme) and labial cervical loop (epithelial), following the same trend as what we observed by FC analysis (newly shown in the **Figure 6e-g**). This further proves the specificity of the used molecular markers of macrophages and the efficiency of used pharmacological treatment.

We commented this in the text as follows:
Page 10, line 36; Page 11 lines 1-2: "*Likewise, quantification of AIF1 expression by immunohistochemistry on paraffin sections revealed a reduced number of AIF1+ cells in both the dental pulp (Fig. 6f) and the epithelial compartment of LaCL (Fig. 6g)."*

Taken together, our newly performed experiments and the presented results provide strong additional evidence that we specifically targeted the macrophage population, with only theoretical little to no effect on other cell types.

2. The authors started with the identification of the Csf1 ligand and its receptor Csf1R within the developing teeth and found that they are ubiquitously express in both mesenchyme and epithelium. (Sup fig.1)

We thank the reviewer for this comment. We believe this is just a misunderstanding. We would like to clarify that **only the, the expression of *Csf1* (ligand) was observed throughout the mesenchymal** pulp population (and some other areas), whereas the expression of ***Csf1r* (receptor) was very specifically expressed only in macrophages** and absent in other regions/cell types.

We agree that this may not have been sufficiently clearly shown in the previous version. Based on this comment we decided to significantly revise and reorganise this figure, now presented as **Supplementary Figure 3**.

We have added **clearer labels** which are now positioned **next** to the images (not inside), indicating whether the **Csf1 (ligand)** or the **Csf1r (receptor)** was detected. In addition, we introduced **arrows** and **color-coded semi-transparent regions** to better illustrate the spatiotemporal distribution of both probes. Along with that we introduce detailed labels of various histological structures which includes: **Am. = ameloblasts, Cond. Mesen. = condensed mesenchyme, DF = dental follicle, EO = enamel organ, IK = initiation knot, DP = dental pulp, LaCL = labial cervical loop, LiCL = lingual cervical loop, Od. = odontoblasts, Mϕ = macrophages, pre-od = pre-odontoblasts, pre-am = pre-ameloblasts.**

This information has been incorporated into the main text as follows:

Page 7, lines 6-9: *"…The expression of the Csf1 in the dental tissue was not restricted to the adult stages only, but both Csf1 and Csf1r were present in the developing molars and incisors within dental papilla/pulp, dental follicle, odontoblast layer and inside of the dental epithelium (Supplementary Fig. 3)."*

As detailed above, we confirmed through multiple methodologies – including scRNA-seq, immunohistochemistry, *in situ* hybridization, and various analyses in genetically modified and pharmacologically depleted animals, that *Csf1r* is a highly specific macrophage-specific molecular marker, co-expressing Aif1, F4/80 and many other established macrophages-specific genes.

3. They selected to use Wnt1cre driver mouse to remove Csf1 in the mesenchymal cell lineage. The rationale is unclear. Will removing Csf1 in epithelial lineage give rise to the comparable results? Are these cells limited to macrophages? As dental development is epithelial-mesenchymal interaction, changes in one compartment can affect the other compartment.

We thank the reviewer for this comment and have made our rationale clearer.
The rationale standing behind the use of $Wnt1^{Cre}/Csf1^{fl/fl}$ animals, was that the majority of the tooth and tooth-associated tissue (importantly, excluding dental epithelium) originate from the *Wnt1+* neural crest. Meanwhile, the epithelial part (enamel organ including ameloblasts, stellate reticulum, stratum intermedium, outer and inner enamel epithelium, and finally enamel) is in mice and human derived from the oral ectoderm.

In the new **Figure 1**, we demonstrate that the presence and establishment of macrophages occur initially and exclusively in the condensed mesenchyme during tooth development, first detected at stage E12.5 beneath the initiation knot. Macrophages are not observed within the enamel organ (epithelium) until stage E17.5. At this timepoint all the mesenchymal parts are already colonized by macrophages and macrophages start to migrate also into enamel organ (**Figure 1c, Supplementary Figure 1b, Supplementary Figure 3d**). **These findings suggest that macrophages first populate the neural crest–derived ectomesenchyme and only subsequently migrate into the epithelial compartment.**

To reflect this, we commented on this in results, as follows:

Page 5, lines 19-31: *"Starting from E12.5 (Embryonic day 12.5, post coitum), we detected AIF1+ macrophages in the periphery of the condensed mesenchyme (Fig. 1a). Later, by E15.5 and E17.5, their expansion gradually increased throughout the dental papilla/pulp. Macrophages appeared homogenously spread in the dental papilla/pulp both before and after birth. This included the apical and central part of the pulp, pulp horns and the odontoblast layer (Fig. 1a-c). The abundance of AIF1+ cells in the dental papilla/pulp dramatically increased shortly after birth (Fig. 1d-g). At embryonic timepoints E12.5-E17.5 and P0 (postnatal day 0), the mean of macrophages-positive area ranged from 0.75-2%, while during later, still pre-eruptive P3-P14 stages it ranged from 9.5-13% (Fig. 1h). Besides the dental papilla/pulp mesenchyme, the presence of AIF1+ cells was also observed in the close surroundings of the tooth, including the superior wall of the alveolar crypt (the space between the enamel organ and the oral epithelium) and the dental follicle at different time points (Fig. 1b–g; Supplementary Fig. 1a–c). The presence of AIF1+ cells was also detected during later root elongation process at P14 in the dental follicle root-growing space (Fig. 1g).*

Page 7, lines 12-15: *"Our results suggest that macrophages firstly colonize the neural-crest derived ectomesenchyme and later on, migrate into the epithelial compartment. Therefore, genetically-modified mouse allowed us to investigate the role of macrophages in teeth after targeted ablation of Csf1 in dental mesenchyme and other associated neural crest derivatives (Fig. 3)."*

And we commented on this in the discussion as follows:

Page 13, lines 10-23: *"We observed macrophages as early as the bud stage (E12.5) of tooth development, localized exclusively within the dental mesenchyme but absent from the epithelium. During the development through the late bud, cap, and bell stages (E13.5–E17.5), macrophages increasingly populated the dental papilla, follicle, and enamel organ. Interestingly, macrophage abundance increased markedly after birth, resembling patterns seen in other organ systems (Dick et al, 2022). However, further lineage-tracing studies will be required to confirm this relationship.*
In the incisor, we additionally mapped macrophage distribution according to specific regions and associations to diverse cell populations. We identified circulating macrophages associated with blood vessels, proliferating tissue macrophages in dental pulp, macrophages interspersed among odontoblasts, those occupying the dental follicle, macrophages that migrated into the dental epithelium – particularly in the labial cervical loop where the epithelial stem cell niche is located (Juuri et al, 2012; Krivanek et al, 2023). The compartment-specific distribution implies that macrophage functions are context-dependent, modulating the microenvironment according to the local developmental or regenerative demands of each region."

Interestingly, the *Csf1* (ligand) is expressed not only in the dental pulp, but also in the dental epithelium of continuously growing teeth (the labial cervical loop), as shown in the **Figure 3a–d**. Based on this and our results using *Wnt1*^{Cre}/*Csf1*$^{fl/fl}$ animals, we hypothesize that macrophages populate the dental epithelium (also expressing *Csf1* ligand) through the mesenchyme. Once this is affected, macrophages fail to populate not only the neural-crest-origin mesenchyme, but dental epithelium as well.

We agree that this using an epithelium-specific *Cre* driver, such as *Krt14*^{Cre} together with *Csf1*$^{fl/fl}$ animals might show something interesting, however, unfortunately, introduction such a new strain (new ethical approval, embryo-transfer, several generations of breeding, experiments themselves) would probably take several 2-3 years with very unsure outcome. Thus, within the timeframe of the revision, it was not feasible to perform such a complex new experiment.

4. For example, in fig.2 Are the cells that co-express Csf1 and derived from Wnt1-cre express markers for macrophages only or other cells markers too? Another interesting point in the fig.2 is that AIF1+ cell shows different residing locations between incisor and molar, do you have any thoughts on these different phenomena?

We touched this partially in the first comment. Our data show that macrophages express Csf1r (receptor) – they do not express the Csf1 (ligand).
To better explain the population structure from scRNA-seq data (now in **Figure 3a**) and its relation to the expression of the *Csf1* (ligand) in all sequenced cells (**Figure 3b**), we now **added detailed annotation** of all cell clusters of scRNA-seq data. These data show expression of *Csf1* in the dental pulp cells and some other cell types and show that Csf1 is almost not expressed in the cluster of macrophages.

Indeed, the distribution of (AIF1+) macrophages differs between molars and incisors as our data suggest. We hypothesize that it is mainly due to their different growth potential and maintenance of stem cell niche – specifically observed it the labial cervical loop of the incisor.

To better show the difference between the distribution of macrophages in molars (and their surrounding) and incisors, we now provide a more in-depth visualization and description of spatio-temporal progression/establishment of AIF1+ macrophages in the zones critical for tooth development and eruption (superior wall of the alveolar crypt, dental follicle) during embryonic development (E15.5, E17.5) and later postnatal stages (P0, P6, P14) as seen in the new **Figure 1a-g** and **Supplementary Fig.1 a-c.** We comment on this in the text as follows:

Page 5, lines 26-31: *"…Besides the dental papilla/pulp mesenchyme, the presence of AIF1*$^{+}$ *cells was also observed in the close surroundings of the tooth, including the superior wall of the alveolar crypt (the space between the enamel organ and the oral epithelium) and the dental follicle at different time points (Fig. 1b–g; Supplementary Fig. 1a–c). The presence of AIF1+ cells was also detected during later root elongation process at P14 in the dental follicle root-growing space (Fig. 1g)."*

To address incisors, in the revised manuscript, we now include new panels (**Figure 1j**) which illustrate the spatial distribution of macrophage populations across the whole apical part of mouse incisor. Specifically, we observe macrophages associated with blood vessels (yellow color-coded), likely representing recently extravasated monocytes that have just entered the tissue. Additionally, we identify distinct populations located within the dental follicle (cyan), along the odontoblast layer (green), and resident within the dental pulp (orange). The latter are characterized by co-localization

with mKI67 (**Figure 1i,j**), suggesting *in situ* proliferation and a potential tissue-resident phenotype. We comment on these in the main text as follows:

Page 6, lines 2-7: *"…Co-staining of macrophages with mKI67, a marker of proliferating cells, revealed their proliferative capacity within the teeth, suggesting that these cells are pulp-resident and maintained through local proliferation. (Fig. 1i). The spatial distribution of macrophages in this tooth was not stochastic, but have shown an association with other cell types and histological structures such as neurovascular bundle, the odontoblast (and sub-odontoblast) layer, dental pulp cells or dental follicle region (Fig. 1j)"*

In parallel we show the new panel **Figure 2e** and the reworked panel **Figure 2f** with a detailed perspective of the labial cervical loop.

5. In fig. 3b, other populations such as glia, epithelium, and non-labelled population in the middle of the panel also express Csf1R too.

We thank the reviewer for this valuable comment. Indeed, this type of 2D embedding visualization of gene expression has certain limitations (it was primarily designed for easy visual gene expression clarity) and may give the impression that Csf1r is rarely expressed in other cell types as well. To address this, we now provide quantitative data from differential gene expression analysis focusing on *Csf1r* and macrophages (**Supplementary Figure 2d**). These results demonstrate that *Csf1r* expression is extremely specific to macrophages (and partially to a very minor population of lymphocytes), whereas other cell clusters such as glial or epithelial cells show statistically insignificant *Csf1r* expression. We now show that *Csf1r* is co-expressed with other known markers of macrophages like *Aif1*, *F4/80***,** Cxcl16, etc. which further proves the character of this cluster.
According to this data we adapted the text as follows:

Page 6, line 36; Page 7, lines 1-4: "…*To confirm the identity of Csf1r+ cell cluster as macrophages we performed a differential gene expression (DGE) analysis which proving the co-expression of other well-established molecular identifiers of macrophages (e.g., F4/80, Aif1, Mrc1, Cxcl16 and others) (Supplementary Fig. 2d) (Wei et al, 2023; Abdelaziz et al, 2020; Kim et al, 2019).*"

6. The rescued result of transplantation experiment is very exciting. Are populated AIF1+ cells from the donor or the host? The quantification of these cells will enhance the strength of the study.

This is a very valid point that we are now clearly addressing. In our revised manuscript we quantified the amount of double-positive (GFP+/AIF1+) cells per area in the dental pulp of molars of *Csf1r KO* and *Csf1r KO* + BMT showing that **after the bone marrow transplantation in *Csf1r KO* animals all macrophages that populate the pulp are derived from the donor** (they are GFP+/AIF1+ double-positive)**.** This new data can be found in the updated version of **Figure 5h**, and we adapted accordingly the text as follows:

Page 9, lines 16-23: *"We conducted a bone marrow transplantation (BMT) via single intraperitoneal (i.p.) administration of a healthy mouse GFP+ whole bone marrow into P1 mice (Fig. 5e-h). To assess the success rate of BMT in relation to dental tissue, we quantified the engraftment of the*

transplanted cells by IHC co-staining of AIF1 and GFP. Although in the dental tissue of Csf1r KO animals we did not observe any AIF1+/GFP+ cells, after BMT the number of AIF1+/GFP+ cells increased to 52.15 ± 4.13 cells per region of interest (ROI) in the dental pulp. This demonstrated the efficient infiltration and establishment of transplanted cells within the dental pulp and their contribution in the phenotype rescue (Fig. 5h).”

7. Data in the fig.4 is also remarkably interesting and exciting. However, I hope to see stronger evidence that this result is directly caused by the depletion of macrophage population. What are the mechanistic insights occurring at the macrophages- does the apoptotic macrophage cell number increase vs mesenchymal/epithelial apoptotic cell number, etc.?

Thanks for the reviewer´s feedback. To clarify the mechanistic basis of our findings, we first elaborated on how clodronate liposomes induce macrophage cell death. This explanation has been added to the revised manuscript, as follows:

Page 10, lines 7-11: *“Clodronate liposomes (belonging to a larger group of bisphosphonates) are widely used drugs that specifically eliminate macrophages and some of their tissue derivatives in living organisms (Xia et al, 2024; Sears et al, 2022). This drug is engulfed by metabolically active phagocytic cells, such as macrophages, and converted into a toxic ATP analogue that inhibits mitochondrial translocase resulting in cell death (Frith et al, 1997; Lehenkari et al, 2002).”*

To strengthen the causal link between macrophage depletion and the observed dental phenotype, we have expanded the original dataset (previously Fig. 4) and now include new experimental evidence presented in the revised **Figure 6**. These new analyses support that the dental defects are indeed a direct consequence of macrophage depletion following Clodrosome treatment. Specifically, we did the following:

1. **Repeated the whole treatment where we added the short-term and long-term conditions and increased biological replicates for better statistical power** (as schematized in the new **Figure 6a**). These conditions were:

    - Acute response (short term): 3 daily injections of Clodrosome, tissue collected on day 4 (n = 6; 3 males, 3 females).
    - Recovery phase (long term): 3 daily injections of Clodrosome, tissue collected on day 10 (n = 6; 3 males, 3 females).

We included this information in the main text as follows:

Page 10, lines 14-21: *“To investigate the role of macrophages in maintaining incisor homeostasis, we implemented different Clodrosome delivery regimens, and examined the dental tissue adaptive response following macrophage depletion (Fig. 6a). Clodrosome was delivered at specific time intervals to understand macrophages contribution upon different conditions: (1) acute response where tissue was harvested one day after three consecutive days of administration, (2) recovered status where tissue was harvested one week after three consecutive days of administration, (3) long-term effect where tissue was harvested after pulsed administration every other day for a week, and maintained once a week for three more weeks (Fig. 6a).”*

2. **Performed quantitative flow cytometry (FC) analysis** on isolated dental pulp cells using the F4/80 (macrophages) and Gr-1 (granulocytes) staining, with a viability dye. Compared with controls, acute Clodrosome induced 3 times reduction in viable F4/80+ cells, **confirming macrophage-specific cell death**. Long-term Clodrosome scenario revealed a recovery of the number of macrophages in the pulp, which proves the transient effect of the bisphosphonates and the ability of new macrophages to establish in the dental tissue.

Page 10, lines 29-36 Page 11, lines 1-2: *"In acute response experiment we detected a significant drop of amount of F4/80+ cells from 15.23 ± 0.25 % (Mean ± standard deviation) (untreated condition) to 4.97 ± 1.46 % in the acute condition. The ratio of F4/80+ macrophages was restored after recovery period to 17.5 ± 4.81 % one week after last Clodrosome delivery (Fig. 6c. Additionally, we analyzed the ratio of Gr-1+ cells (common marker of granulocytes, differentiating monocytes and macrophages) after the Clodrosome treatment. FC analysis showed a similar trend to that observed for F4/80 with ratio of Gr-1+ cells of 1,43 ± 0.35% in controls, 0.66% in acute-response and 1.66% in the recovered group (Fig. 6d)."*

3. **Conducted immunostaining (AIF1 and F4/80)** to assess spatial distribution and co-localization (proven) of macrophage markers. We observed an overall reduction of number of AIF1+ cells in both the dental pulp and epithelial compartments. This data is presented in **Figure 6f,g** and added in the main text as follows:

Page 10, line 36; Page 11 lines 1-2: *"Likewise, quantification of AIF1 expression by immunohistochemistry on paraffin sections revealed a reduced number of AIF1+ cells in both the dental pulp (Fig. 6f) and the epithelial compartment of LaCL (Fig. 6g)."*

4. **Assessed inflammatory response via a cytokine array** analysis to characterize the pro- vs. anti-inflammatory profile induced by macrophage cell death. At the protein level we detected a down-regulation of pro- and anti-inflammatory cytokines which correlated with single-cell RNA seq transcriptomics data from Clodrosome-treated mice. This new data is shown in **Figure 6o,p**, and commented in the text as follows:

Page 12, lines 1-10: "*Bisphosphonate administration has been reported to influence the production of both pro- and anti-inflammatory cytokines, likely through apoptotic macrophage clearance and subsequent immune modulation. in the short term, bisphosphonates reduce pro-inflammatory cytokine production, whereas in the long term, this effect is reversed (Khanna et al, 2024). To investigate this phenomenon in our Clodrosome-treated mice, we employed two complementary approaches. First, pro- and anti-inflammatory cytokine levels were analyzed in isolated dental pulps of incisors using a targeted array profiling. This analysis revealed an overall downregulation of both cytokine classes (Fig. 6o). We then correlated these findings with scRNA-seq transcriptomic data from Clodrosome-treated mice, which similarly showed downregulation of key pro-inflammatory markers, including MIP1β, MIP1α, and TNFα (Fig. 6p), which was consistent with the cytokine assay on protein level.*"

Together, these new experiments provide stronger mechanistic evidence that the observed dental defects are caused by macrophage depletion following Clodrosome treatment, rather than by off-target cytotoxic effects on epithelial or mesenchymal cells.

Minor point

a. Page 6 line 15, do you mean fig 2i instead of 2k?

Thank you for mentioning this. Now fixed.

Reviewer #3 (Remarks to the Author):

Reviewer #3 provided brief comments to the editor only which included general remarks on the manuscript followed by a few technical remarks.

We sincerely appreciate the reviewer's positive feedback and insightful comments. Our detailed responses to all points are provided below

1. This reviewer pointed out that the analysis of the macrophage population in the developing tooth is based on a single marker and that expanding the marker selection and provision of quantitative data would strengthen the results.

We thank the reviewer for this valuable comment. To address this concern, we expanded our analysis of macrophage identity. Using **single-cell transcriptomics** data, we performed a differential gene expression (DGE) analysis from a healthy mouse incisor dataset, which demonstrated that ***Aif1*** is highly and specifically expressed within the macrophage cluster, together with many other established macrophage markers such as *F4/80*, *Csf1r*, *Mrc1*, *Cxcl16 etc.* This new data is included in the updated version of **Supplementary Figure 2d**.

Furthermore, we validated these findings by **co-localization of F4/80 and AIF1 through immunohistochemistry** in both control and Clodrosome-treated animals, as presented in **Figure 6e**.

In addition, we revised the **Supplementary Figure 3** to better visualize the spatiotemporal distribution of macrophages characterized by expression of ***Csf1r* (*in situ* hybridization)** during tooth development, from prenatal (E12.5–E17.5) to postnatal (P0, P14) stages.

We adapted the main text as follows:

Page 6, lines 36; Page 7, lines 1-4: "…*To confirm the identity of Csf1r+ cell cluster as macrophages we performed a differential gene expression (DGE) analysis which proving the co-expression of other well-established molecular identifiers of macrophages (e.g., F4/80, Aif1, Mrc1, Cxcl16 and others) (Supplementary Fig. 2d) (Wei et al, 2023; Abdelaziz et al, 2020; Kim et al, 2019).*"

Page 10, lines 22-26: "…Immunohistochemical and flow cytometric (FC) analyses confirmed partial depletion of macrophages following Clodrosome treatment, with a recovery of macrophage numbers after a one-week break of Clodrosome treatment (Fig. 6b-g). Validation was based on

the detection of the surface macrophage marker F4/80 and the intracellular marker AIF1, which were found to co-localize within the same cell population (Fig. 6e; Supplementary Fig. 2d)."

We believe that these additional analyses strengthen the molecular identity of the macrophage population in the tooth.

2. They also expressed the same concerns as Reviewer #2 about limitations the animal models used in this study.

We thank the reviewer for this comment. We took this point seriously and, in the revision, strengthened our evidence that in our animal models (either *Wnt1*^{Cre}*/Csf1*$^{fl/fl}$ or *Csf1r KO*) the targeted cells are macrophages and that the observed phenotypes are macrophage-dependent.
To validate this, we included the following new set of experiments and analyses:

1. **Macrophage specificity of targets.** We proved this by different methodologies:

   a) scRNA-seq differential expression gene (DGE) analysis: We now provide quantitative single-cell evidence that ***Csf1r*** is highly and specifically expressed in the macrophage cluster and co-expressed with canonical macrophage markers (*Aif1*, *F4/80*, *Mrc1*, *Cxcl16*), whereas expression in other clusters (e.g., glia/epithelium) is negligible. These newly added results are shown in **Supplementary Fig. 2d** referred accordingly in the text as follows:

Page 6, lines 36; Page 7, lines 1-4: "…*To confirm the identity of Csf1r+ cell cluster as macrophages we performed a differential gene expression (DGE) analysis which proving the co-expression of other well-established molecular identifiers of macrophages (e.g., F4/80, Aif1, Mrc1, Cxcl16 and others) (Supplementary Fig. 2d) (Wei et al, 2023; Abdelaziz et al, 2020; Kim et al, 2019).*"

Page 15, lines 8-11: "We showed that *Csf1r* can be used as a specific and reliable marker in dental tissue by single cell transcriptomics data and validated by RNA *in situ* hybridization, and is critical to promote proliferation, survival and differentiation of macrophages (Stanley & Chitu, 2014; Jones & Ricardo, 2013)."

   b) Protein-level validation: Immunohistochemistry co-localization of AIF1 (intracellular) and F4/80 (surface) confirms that the analyzed cells are macrophages. These images are found in **Figure 6e**, and commented accordingly in the text:

Page 10, lines 22-26: "…Immunohistochemical and flow cytometric (FC) analyses confirmed partial depletion of macrophages following Clodrosome treatment, with a recovery of macrophage numbers after a one-week break of Clodrosome treatment (Fig. 6b–g). Validation was based on the detection of the surface macrophage marker F4/80 and the intracellular marker AIF1, which were found to co-localize within the same cell population (Fig. 6e; Supplementary Fig. 2d)."

   c) Spatial ISH mapping: *Csf1r+* cells map to the same regions as AIF1+/F4/80+ macrophages during mouse incisors and molars development (**Supplementary Figure 3**) showing the colonization of Csf1r+ cells first in the dental pulp(mesenchyme), associated to dental

follicle, and later on in close relation with odontoblast and inside of the epithelial compartment in incisors. This is addressed in the text as follows:

Page 7, lines 6-9: *"… The expression of the Csf1 in the dental tissue was not restricted to the adult stages only, but both Csf1 and Csf1r were present in the developing molars and incisors within dental papilla/pulp, dental follicle, odontoblast layer and inside of the dental epithelium (Supplementary Fig. 3)."*

Moreover, in this **Supplementary Figure 3** we improved the labelling and the data is presented in a clearer way to emphasize that Csf1 (ligand) is broad in mesenchyme (and LaCL epithelium of the incisor), whereas Csf1r (receptor) is macrophage-specific, addressing the initial ambiguity.

2. **Orthogonal depletion strategies and rescue.** We utilize various genetically-modified animals and pharmacological approaches to specifically target and deplete macrophages in dental tissue. We elaborate on this as follows:

a) Genetic macrophage-depletion strategies. We utilize two mouse models to investigate the role of macrophages in tooth. Firstly, we used a neural crest-specific *Wnt1*^{Cre}*/Csf1*$^{fl/fl}$ strain to disrupt the Csf1-ligand based signalling in neural crest derivates (all mesenchymal parts of the tooth). Thus *Csf1r+* macrophages which are responsive to this ligand fail to populate the tooth (**Supplementary Fig. 3e**). Second approach use the global knockout for *Csf1r* (*Csf1r KO*). In both scenarios, we detected none or very little of AIF1+ cells (**Figure 3e-h**, **Figure 5f**) in the tissue compared to wildtype controls. We commented on this aspect in the main text as continues:

Page 7, lines 18-21: *"In mutant Wnt1*^{Cre}*/Csf1*$^{fl/fl}$ *mice, AIF1+ macrophages are almost absent in incisors and molars compared to wildtype animals (Fig. 3e-h). Interestingly, although the conditional deletion of Csf1 by Wnt1*^{Cre}*/Csf1*$^{fl/fl}$ *was targeted to the mesenchymal part of the tooth, macrophages were not observed neither in the epithelial compartment of growing teeth (Fig. 3g)."*

Interestingly, our bone marrow transplantation (BMT) experiments in *Csf1r KO* showed that the phenotype rescue of the dental phenotype happens when macrophages derived from transplanted bone marrow re-inhabit the teeth, proved by co-staining of GFP+/AIF1+ cells and **new quantifications** data presented in **Figure 5f-h**. Newly commented in the text:

Page 9, lines 18-23: *"To assess the success rate of BMT in relation to dental tissue, we quantified the engraftment of the transplanted cells by IHC co-staining of AIF1 and GFP. Although in the dental tissue of Csf1r KO animals we did not observe any AIF1+/GFP+ cells, after BMT the number of AIF1+/GFP+ cells increased to 52.15 cells per region of interest (ROI) in the dental pulp. This demonstrated the efficient infiltration and establishment of transplanted cells within the dental pulp and their contribution in the phenotype rescue (Fig. 5h)."*

In this revised version of the manuscript, we included a more in-depth analysis of the morphometric features of teeth and bone improved after BMT. We are adding 3D images obtained by microCT of the lower and upper jaws from different angles (**Figure 5l,m**) and quantifications of teeth and bone sizes (**Figure 5n,r)**. We commented this results in the text accordingly:

Page 9, lines 24-33: *"Further analysis by microCT demonstrated partial dental and bone phenotype rescue by 14 days (Fig. 5l,m), which included recovery of molars and incisors eruption and a general restoration of mandibular and maxillary bone thickness in BMT compared Csf1r KO animals . Furthermore, to assess morphometric features of wildtype versus Csf1r KO and Csf1r KO + BMT animals, we measured the mandibular, molar and incisor lengths (Fig. 5n-r). Although the overall mandibular length remained unchanged among the three groups (Fig. 5o) the incisor length showed a slight but significant increase following BMT (Fig. 5p). Notably, the first maxillary and mandibular molars displayed a significant and more pronounced increase in length in Csf1r KO + BMT mice compared to Csf1r KO controls (Fig. 5q,r). Data obtained from a phenotype rescue experiment thus show that BMT-derived cells promptly reach the dental tissues and provide in situ support to save a phenotype of malformed teeth."*

Therefore, we conclude that the observed rescued phenotype is macrophages-dependent and does not arise from other cell sources being potentially intrinsically affected in our experiments.

b) **Pharmacological depletion of macrophages.** This approach relies on using known macrophage-depleting agent clodronate (bisphosphonates) previously proven to deplete these cells *in vivo*. Newly, we provide quantifications of the macrophages depletion efficiency in tooth utilizing two strategies:

- Flow cytometry (FC). Quantification of F4/80+ cells (macrophages) showed a significant decrease in the number of F4/80+ cells following Clodrosome administration. Number of macrophages was restored one week after treatment cessation (**Figure 6c**). Thus, used drug has a transient effect: macrophages that engulf the Clodrosome undergo apoptosis, but once treatment stops, new population of macrophages promptly repopulate the dental pulp.

We commented this in the text as follows:
Page 10, lines 29-33: *"In acute response experiment we detected a significant drop of amount of F4/80+ cells from 15.23 ± 0.25 % (Mean ± standard deviation) (untreated condition) to 4.97 ± 1.46 % in the acute condition. The ratio of F4/80+ macrophages was restored after recovery period to 17.5 ± 4.81 % one week after last Clodrosome delivery (Fig. 6c)."*

- Immunohistochemistry. This approach revealed a significant drop in number of AIF1+ macrophages affecting to both dental pulp (mesenchyme) and labial cervical loop (epithelial), following the same trend as what we observed by FC analysis (newly shown in the **Figure 6e-g**). This further proves the specificity of the used molecular markers of macrophages and the efficiency of used pharmacological treatment.

We commented this in the text as follows:
Page 10, line 36; Page 11 lines 1-2: "*Likewise, quantification of AIF1 expression by immunohistochemistry on paraffin sections revealed a reduced number of AIF1+ cells in both the dental pulp (Fig. 6f) and the epithelial compartment of LaCL (Fig. 6g).*"

Taken together, convergent evidence from scRNA-seq, IHC, ISH, two genetic models, pharmacological depletion with recovery, and a donor-cell rescue experiment supports that our

manipulations specifically affect macrophages and that the dental phenotypes arise from macrophage loss.

3. Lastly, they pointed to discrepancies in the analysis of pharmacological model of macrophage depletion as they found that the results and M&M sections differ in description of the age, administration strategy and dosage of the drug used for different analyses.

We thank the comment of the reviewer. Based on this, we decided to introduce many changes in our revised manuscript:

1. **We repeated this experiment with an improved experimental scheme**, schematized in the new version of **Figure 6a**. The main improvements here are:

   d) Split of the different conditions of the study (acute, recovered and prolonged). For better clarity, we color coded them (control-black, acute-purple, prolonged- orange and recovered-green) shown as new **Figure 6c,d,f,g,n**.
   e) For each condition we state the animal age.
   f) For each condition we specify for which methodology samples were harvested.
   g) Better labelled and explained Clodrosome regime.
   h) In the earlier version of the manuscript, flow cytometry related experiments were conducted using isolated pulp from P7 mice. Because the sample size was too small to permit statistical analysis, we have removed those data and **repeated the experiment with a larger *n* and an updated administration strategy**, now shown in **Figure 6a,c,d,f,g**.

2. We adapted the Materials and Methods section accordingly:

Page 19, lines 20-29: *"Animals were injected with an amount of 200 µl of the bisphosphonates drug per 30g of body weight. The animals underwent to different regime strategies based on the condition of study: (1) acute: 6 weeks-old mice received 3 consecutives daily i.p. injections (200 µl per 30g of body weight) and harvested one day after; (2) recovered: 6 weeks-old mice received 3 consecutives daily i.p. injections (200 µl per 30g of body weight) and harvested one week later; (3) prolonged; 6 weeks-old mice underwent a regime of 7 injections over 29 days, every two days until day 8 with 100 µl i.p and then, weekly with 100 µl for two weeks (day 15 and day 22). Exclusively, for single cell sequencing (10x), 3 weeks-old mice were used being i.p. injected for three consecutive days and collected on fourth (200 µl per 30 g body weight), for three consecutive days and collected on the fourth."*

Yellow – included or modified during the **first** round of revisions

Green – included or modified during the **second** round of revisions

Reviewer #2 (Remarks to the Author):

I am satisfied with the revision and additional experiments and data the authors provided to my previous concerns and questions. The manuscript was significantly improved with stronger evidence and reads really well, currently. This study is very exciting and will help advance the knowledge of research field.
I also appreciate the opportunity to re-evaluate this manuscript alongside the detailed concerns raised by Reviewer #3. This study addresses an important and underexplored aspect of tooth biology by examining the role of macrophages in tooth development and homeostasis. The work proposes a new axis in which macrophages contribute to tooth development, expanding the traditional view that has largely focused on epithelial–mesenchymal interactions. While I agree with several of Reviewer #3's scientific concerns and believe certain interpretations require more cautious framing and clarification, I also find the overall observations to be novel, conceptually interesting, and potentially impactful for the field.

We truly appreciate the positive feedback on our revised manuscript. We believe that we have addressed the remaining issues in the second round of experimental revision.

We also think that there has been a misunderstanding in case of Reviewer 3, and the vast majority of the comments have already been addressed in our first revision. Somehow, Reviewer 3 missed few results or quantities assessments – for instance in case of FACS-based analysis of macrophages. In this second revision round, we took a chance to better explain and discuss key results and also resolve what appeared as discrepancies. Not least, we performed additional clarifying experiments. We provide a detailed point-by-point breakdown below.

1.The delineation between bone-dependent and tooth-dependent effects - I do observe alteration in mandibular bone morphology, so I think that bone-dependent effects cannot be completely excluded. The current data support a strong association between macrophage depletion and tooth pathology; however, the temporal and causal separation between bone changes and tooth defects remains incompletely resolved.

We appreciate this comment. We are aware that bones and teeth are closely linked in terms of development and function. This includes the indisputable role of Csf1-Csf1r signaling in bone biology, and therefore that the phenotype we observed may not be isolated to teeth—without the role of adjacent bones.

In our revised manuscript, we reflected on this fact and discussed it in detail to emphasize that some of the observed phenotypes in teeth may be related to the bone phenotype:

Page 3, lines 15-18 (Introduction): "***Teeth cannot be considered as isolated organs but should be understood as inseparable part of a complex system in which the surrounding alveolar bone***

along with other periodontal structures *(gingiva, periodontal ligaments and cementum) participate in the tooth development and function".*

Page 17, lines 29-35; Page 18, lines 1-9 (Discussion): *"**The development of alveolar bone and teeth represents a highly integrated and co-regulated process** within the embryonic craniofacial complex. **These tissues do not form independently** but arise through continuous molecular, biomechanical, and immune system-derived interplay. In particular, **macrophages and their CSF1-dependent lineages** emerge as key mediators within this interface, orchestrating osteoclast differentiation, **bone resorption, tooth eruption**, as well as intra-dental epithelial and mesenchymal cell responses. This intricate coupling means that perturbations affecting bone formation and immune cell recruitment to facial tissues during development **are unlikely to remain confined to one compartment**. Instead, **they propagate across the entire alveolo-dental complex, altering root elongation, eruption dynamics, and even enamel and dentin maturation.** Therefore, a mechanistically holistic approach - one that jointly examines odontogenic and osteogenic processes and integrates the contributions of tooth macrophages and stromal cells - is essential for understanding the orchestration of orofacial development. **In this specific context, studying bone and tooth formation in isolation risks overlooking the reciprocal dependencies that define this morphogenetic continuum and shape both normal development and pathological outcomes."***
Overall, our work does not exclude partial bone-dependent effects associated with disruption of Csf1-Csf1r signaling on tooth formation.

Moreover, we further worked on clarifying which phenotypes may involve bone-dependent mechanisms, and provide additional analyses addressing the relationship between bone and tooth defects. This can be found in the manuscript as continues:

A) Findings supporting bone-dependent or mixed phenotypes:

A1) Delayed molar eruption and root elongation and shorter incisors. We characterized macrophage (AIF1+), osteoclast (CTSK+), and pre-osteoclast (AIF1+/CTSK+) populations in molar eruption and root growth regions and in the incisor alveolar socket in *Wnt1*^{Cre}/*Csf1*$^{fl/f}$ at a peri-eruptive stage (P14) (**Fig. 4**). These analyses show reduced macrophage numbers and altered osteoclast differentiation dynamics, associated with **delayed molar eruption, delayed root elongation, and shortened incisors**, consistent with impaired bone resorption. We commented and discussed these new results in the text as follows:

Page 8, lines 15-31 (Results): *"To further elucidate this phenotype, we conducted a detailed characterization of the jawbones in these animals (Fig. 4). Molar eruption and root formation were developmentally delayed (Fig. 4a,b). To quantify these defects, we measured the number of AIF1+, CTSK+ and AIF1+/CTSK+ positive cells in the eruption space and root growth space (Fig. 4c–e), as well as morphometric parameters including eruption distance and root length (Fig. 4f,g). Our results show decrease of AIF1+ cells (macrophages) in mutant mice, however no significant difference was observed in the number of CTSK+ (osteoclasts) or AIF1+/CTSK+ (pre-osteoclasts) cells between mutant and wildtype animals (Fig. 4d,e). The observed delayed molar eruption and root elongation are a direct bone consequence as disrupting Csf1 signaling impaired correct osteoclast differentiation and slows done bone resorption compared to controls. Subsequently, we examined*

the alveolar bone socket of mouse incisor. In contrast to molars, the incisors of mutant mice exhibited a significant increase of AIF1+/CTSK+ cells (pre-osteoclasts) and in parallel a significant decrease of CTSK+ cells (osteoclasts) (Fig. 4h,i) along with a notably wider dental follicle space (Masson trichrome images of Fig. 4h). These findings explain more prominent phenotype observed in incisors compared to molars (Fig. 3i,j)proving the important role of CSF1 signaling for proper differentiation of osteoclasts (Kim & Kim, 2016; Mun et al, 2020) (Fig. 4j). Thus, perturbations in bone resorption during tooth development can influence on its correct formation."

Page 17, lines 25-28 (Discussion): "…*Our data indicate that the some observed dental defects (e.g. single case appearance of supernumerary teeth or the transient disruption of dentin/enamel formation upon clodrosome treatment) originates primarily in teeth. However, in some other phenotypes (delayed molar eruption and root elongation or incisor shorten morphology) the role of surrounding bone cannot be completely excluded."*

A2) Mandibular jaws preserve structural organization with moderate size reduction. We stated that *Wnt1^{Cre}/Csf1$^{fl/fl}$* and *Csf1R KO* mice, the alveolar bone develops with normal morphology, maintaining the overall jaw structure, arguing against a general bone formation defect (**Fig. 3i,j**). This statement required further quantitative support. To address it, we **performed additional morphometric analyses** on the existing microCT **dataset to objectively assess mandibular size and shape parameters in controls, *Wnt1^{Cre}/Csf1$^{fl/fl}$* and *Csf1R KO* mice**. These new measurements are presented in a new **Supplementary Fig. 5f-i**, and commented accordingly in the manuscript as follows:

Page 10, lines 20-36; Page 11, lines 1-6 (Results): "*To address the implication of macrophages depletion during mandibular alveolar bone formation, we performed morphometric analyses using microCT datasets and quantitatively assessed mandibular anteroposterior lengths and vertical heights in control, Wnt1^{Cre}/Csf1$^{fl/fl}$ and Csf1R KO animals. Three-dimensional microCT reconstructions showed that the overall mandibular organization is preserved in Wnt1^{Cre}/Csf1$^{fl/fl}$ mutants, with clearly identifiable alveolar regions (incisor and molar alveoli), a properly positioned mandibular ramus, and formation of the major coronoid, condylar, and angular processes (**Fig. 3i,j and Fig. 5l,m**). To quantitatively assess the changes in the mandibles of Wnt1^{Cre}/Csf1$^{fl/fl}$ and Csf1R KO animals, we performed quantitative morphometric analyses using defined anatomical landmarks (**Supplementary Fig. 5f-i**). The measurements of Wnt1^{Cre}/Csf1$^{fl/fl}$ animals (both 6-weeks or 8-weeks old animals) showed a minor, however statistically significant differences in the anteroposterior dimensions. For 6-weeks Wnt1^{Cre}/Csf1$^{fl/fl}$ mutants A-B and A-C distances were 7.92 ± 0.17 mm and 6.21 ± 0.19 mm, respectively; and for controls 8.57 ± 0.62 mm and 7.09 ± 0.13 mm. In contrast, no statistically significant differences were observed in the vertical height. For 6-weeks old Wnt1^{Cre}/Csf1$^{fl/fl}$ mutants B-C and D-E distances were 4.65 ± 0.04 mm and 2.86 ± 0.06 mm, respectively; and for controls 5.22 ± 0.62 mm and 3.32 ± 0.23 mm (**Supplementary Fig. 5g,h**). On the other hand, 2-weeks old Csf1R KO animals exhibit pronounced variations in mandibular sizes, affecting both anteroposterior and vertical dimensions. Anteroposterior A-B and A-C distances in Csf1R KO jaws were 6.14 ± 0.22 mm and 5.21 ± 0.47 mm compared to aged-matched controls which displayed 6.94 ± 0.14 mm and 6.77 ± 0.10 mm. Vertical dimensions B-C and D-E distances in Csf1R KO jaws were 3.45 ± 0.31 mm and 2.41 ± 0.13 mm compared to controls which sized 4.02 ± 0.06 mm and 2.55 ± 0.05 mm (**Supplementary Fig. 5i**). Together, our data indicate that disruption of Csf1-Csf1r signaling axis can impacts the*

mandible size, which is rather minor in $Wnt1^{Cre}/Csf1^{fl/fl}$ animals and more notable in Csf1R KO animals.

B) **Findings supporting tooth-dependent effects:** We added several analyses suggesting that some phenotypes arise independently of major bone defects.

B1) Appearance of supernumerary teeth. Although the supernumerary tooth phenotype (**Fig. 3j**) was observed with incomplete penetrance (1 out of 4 analyzed *$Wnt1^{Cre}/Csf1^{fl/fl}$* animals), we consider this finding biologically meaningful. The presence of an additional tooth adjacent to the first molar suggests a disturbance in early odontogenic patterning mechanisms prior to the onset of jawbone formation or resorption.

We have revised the manuscript to present this finding in a more cautious manner:

Page 4, lines 30-33 (Introduction): "*Our results show that depletion of macrophages leads to multiple developmental abnormalities, including disrupted tooth morphology, malformations in hard dental tissues, delay of tooth growth, or improper early tooth patterning which in one case was manifested by the presence of a supernumerary tooth*"

Page 7, lines 35-36; Page 8, lines 1-2 (Results): "*Notably, in a single case of the $Wnt1^{Cre}/Csf1^{fl/fl}$ animals' supernumerary tooth was observed (Fig. 3j). Variability of all observed dental phenotypes of incisors and molars is summarized in detail in the Supplementary Table 1 and Supplementary Table 2.*"

Page 15, lines 21-26 (Discussion): "*$Wnt1^{Cre}/Csf1^{fl/fl}$ genotype, in a single case, developed a supernumerary tooth positioned mesial from the first molar. As induction of tooth germs is prior to bone formation in embryogenesis, the presence of supernumerary tooth provides the evidence of tooth-autonomous phenotype stemming from impaired early tooth patterning*."

Page 17, lines 25-28 (Discussion): "*In this study, our data indicate that the some observed dental defects (e.g. single case appearance of supernumerary teeth or the transient disruption of dentin/enamel formation upon clodrosome treatment) originates primarily in teeth. However, in some other phenotypes (delayed molar eruption and root elongation or incisor shorten morphology) the role of surrounding bone cannot be completely excluded."*

B2) Bone formation at early stage is not affected in initial phases of jaw formation. We analyzed bone formation and matrix secretion in analyzing *$Wnt1^{Cre}/Csf1^{fl/fl}$* embryos at E14.5. We performed Masson Trichrome staining on serial sections to evaluate collagen-rich osteoid deposition in the developing alveolar bone region between Meckel's cartilage and the incisor. We complemented this, by RNA *in situ* hybridization for *Sp7* (Osterix) to visualize changes in osteoblast specification. These data are now included in **Supplementary Fig. 5a-e** and described in the Results section.

Page 8, lines 32-35, Page 9, lines 1-5 (Results): "*To study if the observed incisor elongation abnormalities are a secondary cause of disrupting osteoblast lineage or early bone matrix deposition, we evaluated matrix secretion on $Wnt1^{Cre}/Csf1^{fl/fl}$ embryos at E14.5 by Masson Trichrome staining (Supplementary Fig. 5a-c). The extent of collagen-rich osteoid deposition and spatial organization of early bone matrix were not visibly changed. Complementary, we detected RNA in situ hybridization of Sp7 (Osterix), a key transcription factor for osteoblast commitment and*

differentiation, and did not reveal altered patterning of the osteogenic domain (Supplementary Fig. 5d,e). Therefore, ***Csf1 deletion in neural crest–derivatives does not disrupt the establishment of the osteoblast lineage or early bone matrix deposition*** *at the developmental stage preceding incisor elongation".*

B3) Dentin and enamel disorganization in Clodrosome-treated animals. Transient pharmacologically-induced (clodrosome) macrophage depletion in adult mice triggered odontoblast and epithelial disorganization, indicating that macrophage clusters are intrinsically required for maintaining tooth cell integrity and function (**Fig. 6h-k**). This fact, cannot be explained by changes in bone formation since the experiment is performed in fully adult animals, where bone is already fully developed. Despite this, we performed osteoclast detection (TRAP) not showing significant changes upon acute clodrosome regime. We commented these results as follows:

Page 12, Lines 23-30 (Results): "*To exclude the association of observed dental lesions with ectopic activation of osteoclasts, we performed TRAP (Tartrate-resistant acid phosphatase) assay to detect the osteolytic activity of osteoclasts (Fig. 6l-n). Osteoclasts in association with the alveolar bone of the continuously growing incisor are physiologically associated with permanent growth and associated periodontal tissue remodeling (Omi & Mishina, 2022). We detected a normal distribution of osteoclasts in this region and a complete absence of any TRAP+ cells or traces of TRAP staining (both indicating osteoclast activity) (Nakayama et al, 2011) at the site of the enamel and dentin damage (Fig. 6l,m). Thus, the lesion is presumed to have been formed during dentin and enamel patterning and not secondarily*."

To sum up, we believe that the introduced changes in the text and the additional experimental data reconcile the main concerns raised by the reviewer:

- We **avoid** definitive statements claiming a **complete and exclusive tooth autonomy** of the observed phenotypes.
- We explicitly **acknowledge that bone effects** cannot be entirely excluded.
- We **emphasize a coordinated bone–tooth** mechanism.
- We provided **new morphometric data proving an overall maintenance of the structural organization of mandibular jawbones** when Csf1 signaling is disrupted.
- We show **no major effects in osteoblast-lineage at the onset of jawbone formation** in *$Wnt1^{Cre}/Csf1^{fl/fl}$*.

2. Resident vs circulating macrophages effect -The distinction between resident and circulating macrophages remains unclear. The manuscript emphasizes the importance of resident macrophages, yet the rescue experiment suggests that these cells are circulation-derived. I agree that this issue has not been adequately addressed and requires clearer explanation and terminology.

This is completely true. We apologize for bringing in excessive terminology in the previous version of our manuscript, which confused the reviewers. We have corrected this issue in this revision.

The central point of this study was to clarify the role of macrophages on tooth development – regardless of their primary origin (monocyte-derived or are tissue-resident). The origin of macrophages is not

essential for the central message of our manuscript (general role of macrophages in tooth development and homeostasis) and neither of the potential outcomes would change the central message of this study. Indeed, most likely, teeth host both populations of macrophages, including tissue resident cells, similarly to other organs, and any further clarifications will not add new understanding here. Also, it is very likely that in any functional experiments macrophage populations will demonstrate plasticity and cross-compensation, which will make lineage-discriminating and functional experiments unnecessarily hard.

Nevertheless, to avoid any misunderstanding or misinterpretation we decided to address this, by completely removing the terms tissue-resident or tooth resident-macrophages in our results ". **Instead, we have introduced the term, "Tooth macrophages," which encompasses both of these groups.**

We explain this term at the very beginning of the manuscript to avoid any confusion:

Page 2, lines 20-35; Page 3, lines 1-6 (Introduction): "*Macrophages regulate complex cellular dynamics of tissues during development, growth, repair, and homeostasis in all tissues, as reviewed by many research teams (Mass et al, 2023; Guan et al, 2025; Zhao et al, 2024; Theret et al, 2019; Sehgal et al, 2021; Wynn et al, 2013). Beyond their critical immune role as first-line defenders against pathogens, they initiate adaptive immune response, clear apoptotic and senescent cells, and actively shape tissue architecture during organogenesis and regeneration (Kierdorf et al, 2015, Lendeckel et al, 2022; Hatch et al, 2024; Watanabe et al, 2019). Macrophages can be broadly classified according to their developmental origin: (i) monocyte-derived, which differentiate from bone marrow-derived monocytes that circulates in the bloodstream and enter tissue in response of tissue homeostasis or inflammation, and (ii) tissue-resident macrophages, which originates from embryonic erytho-myeloid progenitors in the yolk sac or fetal liver, which seed developing tissues during embryogenesis and can persist into adulthood (Haldar & Murphy, 2014). Tissue-resident macrophages acquire tissue-specific functions and are present in numerous tissues, including e.g., Kupffer cells in liver, microglia in brain, osteoclasts in bone, alveolar macrophages in lungs, Hofbauer cells in placenta, Langerhans cells in skin and many others (Lendeckel et al, 2022; Mass et al, 2023). Importantly, there is increasing evidence that macrophages are also crucial for the maintenance of stem cell niches in various tissues, including intestines, muscles, mammary gland, the hematopoietic niche and others (Manneken & Currie, 2023a; Ratnayake et al, 2021a; Zhou et al, 2023; Kayvanjoo et al, 2024; Winkler et al, 2010). In this study we focus on "tooth macrophages" as a broad term for the macrophage population located in the mice teeth regardless of their primary origin (monocyte-derived or tissue-resident). Despite the advance of studying macrophages in different organs, the role of tooth macrophages in the context of the stem cell niche of incisor and molar morphogenesis still remains a completely unexplored area*".

We introduced these changes to adequate the terminology use in the main text as follows:

Page 5, lines 12-13 (Results): "*Tooth macrophages populate early embryonic developing teeth and expand postnatally before eruption.*"

Page 6, lines 5-7 (Results): "*Co-staining of macrophages with mKI67, a marker of proliferating cells, revealed their proliferative capacity within the teeth, suggesting that these cells are ~~pulp-resident and~~ maintained through local proliferation (Fig. 1*i)"

Page 6, lines 11-12 (Results): "*In tooth, similar to other organs such as mammary gland or salivary gland, ~~resident~~ macrophages are in close contact with heterogenous cell populations as integral part of the microenvironment (Zhou et al,2023; McKendrick et al, 2023).*"

Page 14, lines 6-7 (Discussion): "*Throughout these developmental stages, ~~tissue-resident~~ tooth macrophages emerge as abundant component of the tooth microenvironment*"

Page 17, lines 11-13 (discussion): "*This, coupled with our observation of actively proliferating ~~tissue-resident~~ tooth macrophages in adult teeth, suggests that macrophages quickly repopulate impacted dental tissue.*"

Page 17, lines 19-21 (Discussion): "*In summary, our findings suggest that after injury, the population of ~~tissue-resident~~ tooth macrophages rapidly recovers and activates an M2 healing phenotype which leads to the swift restoration of a healthy tooth morphogenesis*"

Page 18, lines 3-4 (Discussion): "*Therefore, a mechanistically holistic approach - one that jointly examines odontogenic and osteogenic processes and integrates the contributions of ~~resident~~ immune and stromal cells*"

Page 34, lines 6-7 (Figure legend): "*Immunohistochemical co-staining using AIF1 and F4/80 antibodies show co-localization of both markers ~~in dental pulp resident~~ of tooth macrophages.*"

Page 38, line 5 (Figure legend): "*Supplementary Figure 2. Csf1 signaling system is the tooth~~-resident~~ macrophages*"

Moreover, to completely clear that our main aim in the paper was to study tooth macrophages independently from their developmental origin, we included in the introduction this clear statement as follows:

Page 4, lines 10-11: *"This study investigates the role of tooth macrophages independently of their origin (this term does not distinguish between monocyte-derived or tissue resident macrophages) during development, growth and interaction with the stem cell niche during continuous tooth growth."*

3. Supernumerary tooth - It appears that the authors emphasized this phenotype in response to Reviewer #1's previous comment. However, because this was observed in only 1 of 4 mandibles, I agree that it is overemphasized. The authors perhaps should explicitly state that this is a rare observation and present it as an isolated, interesting finding rather than evidence of a generalized patterning defect.

Although the supernumerary tooth phenotype (Fig. 3j) was observed in single case out of 4 analyzed *$Wnt1^{Cre}$/$Csf1^{fl/fl}$* animals, we consider this finding important and biologically meaningful. The presence of a supernumerary tooth suggests a mechanism originating during early odontogenesis (prior bone formation or even resorption). This observation supports the hypothesis that macrophage deficiency can influence an early tooth patterning processes – although only in rare cases.

We have revised the manuscript to present this finding in a more cautious manner. Rather than stating that the phenotype "has to be tooth-autonomous," we now describe it as an observation that evidences a tooth-autonomous role of macrophages during early patterning.

Page 4, lines 30-33 (Introduction): "Our *results show that depletion of macrophages leads to multiple*

developmental abnormalities, including disrupted tooth morphology, malformations in hard dental tissues, delay of tooth growth, or improper early tooth patterning which in one case was manifested by the presence of a supernumerary tooth"

Page 7, lines 35-36; Page 8, lines 1-2 (Results): "*Notably, in a single case of the Wnt1^{Cre}/Csf1$^{fl/fl}$ animals' supernumerary tooth was observed (Fig. 3j). Variability of all observed dental phenotypes of incisors and molars is summarized in detail in the Supplementary Table 1 and Supplementary Table 2.*"

Page 15, lines 21-26 (Discussion): "*Wnt1^{Cre}/Csf1$^{fl/fl}$ genotype, in a single case, developed a supernumerary tooth positioned mesial from the first molar. As induction of tooth germs is prior to bone formation in embryogenesis, the presence of supernumerary tooth provides the evidence of tooth-autonomous phenotype stemming from impaired early tooth patterning.*"

Page 17, lines 25-28 (Discussion): "*In this study, our data indicate that the some observed dental defects (e.g. single case appearance of supernumerary teeth or the transient disruption of dentin/enamel formation upon clodrosome treatment) originates primarily in teeth. However, in some other phenotypes (delayed molar eruption and root elongation or incisor shorten morphology) the role of surrounding bone cannot be completely excluded.*"

To emphasize that the presence of supernumerary tooth concerned only one of four individuals, we have included a detailed breakdown not only of the frequency of occurrence of supernumerary teeth, but also of all other observed phenotypes (Supplementary Tables 1 and 2).

4. Reliance on histology and immunostaining instead of flow cytometry - I agree with Reviewer #3 that flow cytometry would provide a more quantitative assessment of macrophage populations. However, when performed with a well-validated and specific antibody, careful IHC quantification on serial sections can still provide reliable and biologically meaningful information, particularly in preserving spatial tissue architecture that flow cytometry cannot capture. In this context, the IHC analyses, if properly controlled and quantified, are sufficient to support the conclusions regarding macrophage presence and distribution in dental tissues.

We are grateful for this comment. Based on suggestions from reviewers, **during the first revisions we already performed and presented quantitative flow cytometry (FC) analysis** side-to-side with IHC analysis. We believe that this fact was overlooked by mistake.

We performed FC (shown in **Fig. 6c,d**) based on validated, well-known and highly specific antibodies: F4/80 (macrophages) and Gr-1 (granulocytes) staining, with a viability dye. This shows that acute Clodrosome induced 3 times reduction in viable F4/80+ cells, confirming macrophage-specific cell death. Long-term Clodrosome scenario revealed a recovery proving the transient effect of the bisphosphonates and the ability of new macrophages to establish in the dental tissue. This data is accordingly commented in the manuscript as follows:

Page 11, lines 32-36 Page 12, lines 1-3 (Results): *"In acute response experiment we detected a significant drop of amount of F4/80+ cells from 15.23 ± 0.25 % (Mean ± standard deviation) (untreated condition) to 4.97 ± 1.46 % in the acute condition. The ratio of F4/80+ macrophages was restored after recovery period to 17.5 ± 4.81 % one week after last Clodrosome delivery (Fig. 6c).*

Additionally, we analyzed the ratio of Gr-1+ cells (common marker of granulocytes, differentiating monocytes and macrophages) after the Clodrosome treatment. FC analysis showed a similar trend to that observed for F4/80 with ratio of Gr-1+ cells of 1,43 ± 0.35% in controls, 0.66% in acute-response and 1.66% in the recovered group (Fig. 6d).”

We confirmed the specificity of these antibodies directly in our samples by co-detecting AIF1 and same F4/80 antibody, showing specific co-localization (**Fig. 6e**). The **statistical data for IHC analysis (Fig. 6f,g) was done in a controlled way by using 3 biological samples with at least 4 serially collected sections per animal to show the variability across the specimens and in the tissue depth.** This quantifications are presented in **Fig. 6f,g** and adapted in the main text as follows:

Page 12, lines 3-5 (Results): *“Likewise, quantification of AIF1 expression by immunohistochemistry on serial paraffin sections revealed a reduced number of AIF1+ cells in both the dental pulp (Fig. 6f) and the epithelial compartment of LaCL (Fig. 6g).”*

5. Concern about descriptive nature and lack of mechanism – I agree the work is largely descriptive and does not provide deep mechanistic dissection at the molecular level.

We thank the reviewer for this comment and agree that the study is primarily focused on the cellular role of macrophages rather than providing a deep molecular dissection.
To touch the mechanism, in response to comments raised during the first revision, we expanded the manuscript to include additional analyses addressing potential mechanisms of **macrophage–cell communication**. Specifically, **we added a new Fig. 2 presenting ligand–receptor interaction analysis** based on previously published single-cell RNA-seq data (*Krivanek et al., 2020*), including CellChat analysis that **identified putative signaling interactions between macrophages and epithelial cell populations, such as the Lgals9–Cd44 axis**. These computational findings were further **validated with histological immunohistochemistry** assays showing macrophages in close proximity to CD44+ epithelial progenitor cells. In addition, **we included new analyses demonstrating epithelial abnormalities and reduced enamel volume in macrophage-deficient mice** (**Fig. 3k,l** and **Supplementary Fig. 4**).

We emphasized this aspect in the discussion as follows:

Page 14, lines 32-35; Page 15, lines 1-5 (Discussion): *“The mechanisms by which macrophages can exert their influence may be either in providing support for proliferation (Liu et al, 2016), producing growth factors (such as VEGFs) (Lai et al, 2019), or regulation of inflammation (Watanabe et al). Ligand–receptor analysis identified the macrophage Lgals9 ligand and epithelial CD44 receptor as a putative communication axis, suggesting that macrophage–epithelium crosstalk may mediate the regulation of proliferation and differentiation.* ***However, while this data provides an initial insight into macrophage-mediated signalling within the epithelial niche, the precise molecular mechanism by which macrophages influence surrounding epithelial or mesenchymal stem cell niches to ensure proper tooth development remains to be investigated in future studies****.”*

Despite these valid concerns, I believe this manuscript remains well suited for publication in Nature Communications. The study provides the first compelling evidence that macrophages are essential

regulators of incisor epithelial architecture and continuous growth, revealing an unrecognized role for macrophages in tooth development. This expands the current paradigm beyond a purely epithelial–mesenchymal framework. The findings are conceptually novel, extend beyond dentistry into stem cell biology and epithelial morphogenesis, and open an important new direction for the field. With more cautious interpretation of certain phenotypes and clearer framing of macrophage origin and bone contributions, the work represents a significant and broadly relevant advance appropriate for Nature Communications.

We feel grateful for your kind words and for the time spent on critical reading of our manuscript.

Reviewer #3 (Remarks to the Author):

After reading the revised manuscript and an extensive response to reviewers comments, I am not able to confirm that manuscript is improved. This is mainly because the authors have missed many of the important points and gave inconclusive, indirect, or in some places incorrectly interpreted evidence to support their hypotheses.

We truly regret that our reviewer did not see the improvements despite very long and extensive experimental revision. In this new, second revision, with limited time and resources, we again performed additional experimental results and new measurements to provide support to our story, which is fairly simple in its core message – macrophages influence dental development and self-renewal (in case of continuously growing teeth). In fact, we agree (and we made all improvements and revisions already in the previous revision) that bone plays some role in developmental dental defects. We never opposed this opinion, and in this new revision we hope to demonstrate our commitment and good will towards supporting this idea. We hope our new data will show the effects better, and the textual changes bring clarity.

1. The issue of suitability of the experimental models in delineating tooth-dependent effects from the bone dependent effect has not been resolved. Bone and tooth are intricately intertwined and interdependent, both during embryonic development and postnatal homeostasis. Alveolar bone differentiation and matrix secretion commence prior to elongation of the incisor, around E14(.5), and there are reports of various mouse mutants that display premature and accelerated bone differentiation associated with altered phenotype of the incisors. The authors provide no information on the timing of bone formation in Wnt1Cre; Csf1flf/fl embryos, and the correlation with the onset of malformations in the developing incisors is missing. Therefore, the possibility that bone phenotype precedes and conditions tooth development and that changes observed in the incisor are secondary changes cannot be dismissed.

We thank the reviewer for raising this point regarding the temporal relationship between alveolar bone formation and incisor development. We agree that establishing whether early alterations in mandibular bone differentiation precede and potentially condition the incisor phenotype is critical for interpreting the causality of the observed defects in *Wnt1*^{Cre};*Csf1*$^{fl/fl}$ embryos.

As noted by the reviewer, alveolar bone differentiation and matrix deposition initiate around E14.5, prior to significant incisor elongation. To directly address whether early bone formation is altered in our model, we performed additional new experiments and analyses at E14.5.

1) **Assessment of early mandibular bone formation.**

1.1) **Histological evaluation of osteoid deposition**. We performed Masson's Trichrome staining on serial frontal sections of E14.5 wildtype (control) and *Wnt1*^{Cre}*;Csf1*$^{fl/fl}$ mandibles to evaluate collagen-rich osteoid deposition in the developing alveolar bone region between Meckel's cartilage and the incisor.

We did **not** observe differences in:

- The extent of collagen-rich osteoid deposition.
- The spatial organization of early bone matrix.

Our new data indicate that the initiation of mandibular osteogenesis is not impaired or accelerated in mutant embryos at E14.5. The new data are included as a new **Supplementary Fig. 5a-c** and described in the revised manuscript as follows:

Page 8, lines 32-35 (Results): "*To study if the observed incisor elongation abnormalities are a secondary cause of disrupting osteoblast lineage or early bone matrix deposition, we evaluated matrix secretion on Wnt1*^{Cre}*/Csf1*$^{fl/fl}$ *embryos at E14.5 by Masson's Trichrome staining (Supplementary Fig. 5a-c). The extent of collagen-rich osteoid deposition and spatial organization of early bone matrix were not visibly changed.*"

1.2) **Detecting changes in osteoblast specification (*Sp7* expression).** To assess whether early osteoblast differentiation is affected, we performed RNA *in situ* hybridization for *Sp7* (Osterix), a key transcription factor required for osteoblast commitment and differentiation.

At E14.5, *Sp7* expression displayed comparable spatial distribution between control and mutant embryos, localized between Meckel's cartilage and the incisor epithelium. Therefore, we concluded that there is**:**

- No premature nor expanded osteoblast differentiation.
- No delayed osteogenic specification.
- No altered spatial patterning of the osteogenic domain.

These findings indicate that **Csf1 deletion in neural crest–derived tissues does not disrupt the establishment of the osteoblast lineage or early bone matrix deposition** at the developmental stage preceding incisor elongation. These new data are included as a new **Supplementary Fig. 5d,e** and are described in the revised manuscript as follows:

Page 8, line 35; Page 9 lines 1-5 (Results): *"Complementary, we detected RNA in situ hybridization of Sp7 (Osterix), a key transcription factor for osteoblast commitment and differentiation, and did not reveal altered patterning of the osteogenic domain (Supplementary Fig. 5d,e). Therefore,* ***Csf1 deletion in neural crest–derivatives does not disrupt the establishment of the osteoblast lineage or early bone matrix deposition*** *at the developmental stage preceding incisor elongation".*

2) **Interpretation of bone resorption around the incisor at P14.**

In previous round of revisions, **we expanded our study utilizing *Wnt1^{Cre}/Csf1$^{fl/fl}$* mice model with a postnatal day 14.** This developmental stage serves as a showcase timing to characterize changes in the bone and epithelium present above the first maxillary incisor and the root growing zone, as well as the changes in the alveolar bone resorption surrounding the apical part of the incisor.

As the incisors in *Wnt1^{Cre}/Csf1$^{fl/fl}$* animals exhibit reduced length but not arrested tooth formation, we investigated whether this phenotype could be explained by impaired bone resorption. To address this, we performed IHC co-staining and quantifications for osteoclasts (CTSK+) and macrophages (AIF1+), alongside conventional histological staining (Masson Trichrome), presented in **Fig. 4h,i.** We commented these results as follows:

Page 8, lines 24-30: *"Subsequently, we examined the alveolar bone socket of mouse incisor. In contrast to molars, the incisors of mutant mice exhibited a significant increase of AIF1+/CTSK+ cells (pre-osteoclasts) and in parallel a significant decrease of CTSK+ cells (osteoclasts) (Fig. 4h,i) along with a notably wider dental follicle space (Masson's Trichrome images of Fig. 4h). These findings explain more prominent phenotype observed in incisors compared to molars (Fig. 3i,j) and suggest the important role of CSF1 signaling for proper differentiation of osteoclasts (Kim & Kim, 2016; Mun et al, 2020) (Fig. 4j)".*

Therefore, our analyses revealed:

- A significant reduction in terminally-differentiated osteoclasts (CTSK+ cells) in **Fig. 4a,b,d,e.**
- A significant accumulation of AIF1+/CTSK+ double-positive cells, representing chimeric monocyte/osteoclast-like (pre-osteoclast-like) cells. in **Fig. 4h-j**.
- Widening of the dental follicle space in **Fig. 4h**.

2. I disagree with their statement that the jaw in these mutants is of correct shape and the overall fine structure of the jaw is maintained, because it is not supported by any evidence: Figure 3j shows mutant mandible of significantly decreased size and altered shape when compared to control (3i). Without quantification analysis there is no proper evidence to support their conclusion, particularly in the light of insufficient sample size (two 6 weeks old and two 8 weeks old samples for Wnt1Cre; Csf1fl/fl line).

We appreciate the reviewer's comment and agree that our previous statement regarding preservation of general jaw architecture required quantitative support. Also, we never stated that bone change is absent, we only stated that bone morphology is not strong, with general preservation of all features (even though they demonstrate minor shape changes, which we briefly discussed). To address this concern even better in this new revision, we performed additional morphometric analyses on our microCT datasets to objectively assess mandibular size and shape parameters in wildtype, *Wnt1^{Cre}/Csf1$^{fl/fl}$* and *Csf1R KO* animals.

<u>1) Qualitative assessment from microCT data.</u> Anatomically, the three-dimensional microCT reconstructions (**Fig. 3i,j** and **Fig. 5l,m**) show that:

- The overall mandibular organization is preserved in *Wnt1^{Cre}/Csf1$^{fl/fl}$* animals.
- The alveolar regions (incisor alveolus, molar alveolus) and the mandibular ramus are present and properly positioned.
- Major mandibular processes (coronoid, condylar, and angular) are formed in mutants.

Although all these structures are present, their proportions slightly differ from controls, with reduction in overall size and subtle alterations in process length and thickness. To address this in detail, we performed quantitative morphometric analyses.

2) Quantitative morphometric analysis. To objectively evaluate mandibular dimensions, we defined reproducible anatomical landmark points:

- **A** – superior rim of incisor alveolus
- **B** – tip of coronoid process
- **C** – superior point of inferior ramus border
- **D** – inferior point of mandibular symphysis
- **E** – superior point of molar alveolus

Distances were measured to assess anteroposterior length (X-axis: A–B and A–C) and vertical height (Y-axis: B–C and D–E). This quantitative analysis demonstrates:

- *Wnt1^{Cre}/Csf1$^{fl/fl}$* animals show:
  - No significant differences in vertical height measurements (B–C and D–E) at either 6 or 8 weeks.
  - No difference in A–B at 6 weeks, with mild reduction observed at 8 weeks.
  - A consistent but moderate reduction in A–C length measurements.
- *Csf1R* KO animals exhibit the more significant mandibular defects, affecting both length and height.

The schematic representation of the landmarks and the measurements are presented in **Supplementary Fig. 5h–k**. We commented the results in the main text of the manuscript as follows:

Page 10, lines 20-36; Page 11, lines 1-6 (Results): "*To address the implication of macrophages depletion during mandibular alveolar bone formation, we performed morphometric analyses using microCT datasets and quantitatively assessed mandibular anteroposterior lengths and vertical heights in control, Wnt1^{Cre}/Csf1$^{fl/fl}$ and Csf1R KO animals. Three-dimensional microCT reconstructions showed that the overall mandibular organization is preserved in Wnt1^{Cre}/Csf1$^{fl/fl}$ mutants, with clearly identifiable alveolar regions (incisor and molar alveoli), a properly positioned mandibular ramus, and formation of the major coronoid, condylar, and angular processes (**Fig. 3i,j and Fig. 5l,m**). To quantitatively assess the changes in the mandibles of Wnt1^{Cre}/Csf1$^{fl/fl}$ and Csf1R KO animals, we performed quantitative morphometric analyses using defined anatomical landmarks (**Supplementary Fig. 5f-i**). The measurements of Wnt1^{Cre}/Csf1$^{fl/fl}$ animals (both 6-weeks or 8-weeks old animals) showed a minor, however statistically significant differences in the anteroposterior dimensions. For 6-weeks Wnt1^{Cre}/Csf1$^{fl/fl}$ mutants A-B and A-C distances were 7.92 ± 0.17 mm and 6.21 ± 0.19 mm, respectively; and for controls 8.57 ± 0.62 mm and 7.09 ± 0.13 mm. In contrast, no statistically significant differences were observed in the vertical height. For 6-weeks old Wnt1^{Cre}/Csf1$^{fl/fl}$ mutants B-C and D-E distances were 4.65 ± 0.04 mm and 2.86 ± 0.06 mm, respectively; and for controls 5.22 ± 0.62 mm and 3.32 ± 0.23 mm (**Supplementary Fig. 5g,h**). On the other hand, 2-weeks old Csf1R KO animals exhibit pronounced variations in mandibular sizes, affecting both anteroposterior and vertical dimensions. Anteroposterior A-B and A-C distances in Csf1R KO jaws were 6.14 ± 0.22 mm and 5.21 ± 0.47 mm compared to aged-matched controls which displayed 6.94 ± 0.14 mm and 6.77 ± 0.10 mm. Vertical dimensions B-C and D-E distances in Csf1R KO jaws were 3.45 ± 0.31 mm and 2.41 ± 0.13*

*mm compared to controls which sized 4.02 ± 0.06 mm and 2.55 ± 0.05 mm (**Supplementary Fig. 5i**). Together, our data indicate that disruption of Csf1-Csf1r signaling axis can impacts the mandible size, which is rather minor in Wnt1^{Cre}/Csf1$^{fl/fl}$ animals and more notable in Csf1R KO animals.*

Taken together, our new data show that the early onset on alveolar bone formation is NOT affected in *Wnt1^{Cre}/Csf1$^{fl/fl}$* embryos so the early tooth phenotype originates autonomously. Although, the adult *Wnt1^{Cre}/Csf1$^{fl/fl}$* animals exhibit measurable reduction in mandibular length, the moderate nature of these dimensional changes does not support a model of gross mandibular malformation driving the dental phenotype. Importantly, alveolar compartments are formed, vertical dimensions are maintained, and tooth development is not arrested.

3. Further, the importance of the supernumerary tooth formation is overstated and inappropriately emphasized considering it is observed in only one sample out of 4. This conclusion requires bigger sample size, high frequency of the phenotype and deeper analyses of the embryonic tooth development in these mutants. For example, it is critical to know whether it is unilateral or bilateral change, and also whether macrophage distribution at appropriate developmental stages reflects differences between the normally toothless diastema region where they observe the extra tooth and the rest of the dentition. Without these analyses, this phenotypic change should be considered an observation, rather than conclusive evidence of the impacted early development and patterning.

Thanks for this comment. Although the supernumerary tooth phenotype (Fig. 3j) was observed in single case out of 4 analyzed *Wnt1^{Cre}/Csf1$^{fl/fl}$* animals, we consider this finding important and biologically meaningful. The presence of a supernumerary tooth **suggests a mechanism originating during early odontogenesis (prior bone formation or even resorption).** This observation supports the hypothesis that macrophage deficiency can influence an early tooth patterning processes – although only in rare cases.

However, we tend to agree with the reviewer, and we have revised the manuscript to present this finding in a more cautious manner. Rather than stating that the phenotype "has to be tooth-autonomous," we now describe it as an observation in one single case that might evidence a tooth-autonomous role of macrophages during early patterning.

Page 4, lines 26-29 (introduction): "*Our results show that depletion of macrophages leads to multiple developmental abnormalities, including disrupted tooth morphology, malformations in hard dental tissues, delay of tooth growth, or improper early tooth patterning which in one case was manifested by the presence of a supernumerary tooth.*"

Page 7, lines 33-36 (results): "*Notably, in a single case of the Wnt1^{Cre}/Csf1$^{fl/fl}$ animals' supernumerary tooth was observed (Fig. 3j). Variability of all observed dental phenotypes of incisors and molars is summarized in detail in the Supplementary Table 1 and Supplementary Table 2.*"

Page 14, lines 16-20 (discussion): "*Wnt1^{Cre}/Csf1$^{fl/fl}$ genotype, in a single case, developed a supernumerary tooth positioned mesial from the first molar. As induction of tooth germs is prior to bone formation in embryogenesis, the presence of supernumerary tooth provides the potential evidence of tooth-autonomous phenotype stemming from impaired early tooth patterning.*"

Page 16, lines 18-20 (discussion): "*In this study, some of our data suggest that macrophage depletion leads to tooth-autonomous phenotypes, such as the formation of supernumerary teeth in Wnt1^{Cre}/Csf1$^{fl/fl}$ mice and transient disruptions in enamel and dentin following prolonged Clodrosome treatment.* ***However, the role of impacted adjacent bone on tooth phenotype cannot be excluded completely."***

To emphasize that the presence of supernumerary tooth concerned only one of four individuals, we have included a detailed breakdown not only of the frequency of occurrence of supernumerary teeth, but also of all other observed phenotypes (Supplementary Tables 1 and 2).

4. Overall, judging by the more penetrant phenotypic changes of their mutant lines, as well as the treated animals, it is very likely that they are secondary to the alterations in mutant mandibular bone (i.e. bone turnover) that hamper continuous growth of the incisor, hence the folding.

We thank the reviewer for this comment. We already provided our response above to cover the situations with dental phenotypes in embryos with ablated macrophages. Our new bone analysis at E14.5 of bone formation (**Supplementary Fig. 5a-e**) and new morphometric measurements (**Supplementary Fig. 5f-i**) of bone formation and shape shall solve the issue (as commented in details above).

Regarding the clodrosome treatment delivered to adult animals is a strongest argument for tooth-autonomous effects since in adult animal's bone turnover is slow, and the incisor self-renewal is extraordinary fast. Also, we do not observe any bone defects in general in adult treated animals in the vicinity of the failing teeth (**Fig. 6h-n**). Moreover, we do not observe any TRAP activity in the lesion of the affected tooth **(Fig. 6l,m**). We hope this is more clear now for the reviewer. We made a special statement about it in our revised manuscript:

Page 12, lines 21-35; Page 13, lines 1-6 (Results): *"…Clodronate is commonly used to treat diseases associated with improper bone metabolism whereby its mechanism of action is to affect osteoclasts, cells derived from macrophages that have the ability to resorb hard tissue. To exclude the association of observed dental lesions with ectopic activation of osteoclasts, we performed TRAP (Tartrate-resistant acid phosphatase) assay to detect the osteolytic activity of osteoclasts (Fig. 6l-n). The results showed a physiologically normal distribution of osteoclasts in the adjacent alveolar bone but a complete absence of any osteoclast activity at the site of the enamel and dentin damage (Fig. 6l,m). Thus, the lesion is presumed to have been formed during dentin and enamel patterning and not secondarily. To further investigate this, we analyzed the dynamics of osteoclast activity in the jawbone following Clodrosome administration by assessing TRAP+ staining in the incisor alveolar bone socket. While acute treatment caused a slight but insignificant reduction of osteoclast activity, a significant increase was observed one-week after treatment cessation (Fig. 6n). This delayed response likely reflects a compensatory upregulation of osteoclast activity during the recovery phase. Importantly, given the relatively slow turnover of alveolar bone in adult animals and the absence of detectable bone defects around incisors at the time of analysis, these findings further support a tooth-autonomous origin of the observed lesions, consistent with the rapid self-renewal dynamics of the incisor."*

We hope we explained our results and made sufficient changes in the revised manuscript to cover this point.

5. Discerning the role of resident macrophages separately and independently of circulatory macrophages is also not resolved in the resubmitted manuscript. It seems that the authors refer to resident macrophages as the population found in the dental tissues. However, their rescue experiment shows that this population derives fully from the circulation, which is in line with the Csfr1 knockout that shows more penetrating phenotype than the Csf1 conditional knockout.

We apologize for the excessive terminology we used in the previous version of our manuscript, which diverted the thought of a reader away from the central message. We have corrected this issue in this revision.

The central point of this study was to clarify the role of macrophages on tooth development – regardless of their primary origin (monocyte-derived or are tissue-resident). The origin of macrophages is not essential for the central message of our manuscript (general role of macrophages in tooth development and homeostasis) and neither of the potential outcomes would change the central message of this study.

Nevertheless, to avoid any misunderstanding or misinterpretation we decided to address this, by completely removing the terms tissue-resident or tooth resident-macrophages in our results ". **Instead, we have introduced the term, "Tooth macrophages," which encompasses both of these groups.**

We explain this term at the very beginning of the manuscript to avoid any confusion:

Page 2, lines 20-35; Page 3, lines 1-6 (Introduction): "*Macrophages regulate complex cellular dynamics of tissues during development, growth, repair, and homeostasis in all tissues, as reviewed by many research teams (Mass et al, 2023; Guan et al, 2025; Zhao et al, 2024; Theret et al, 2019; Sehgal et al, 2021; Wynn et al, 2013). Beyond their critical immune role as first-line defenders against pathogens, they initiate adaptive immune response, clear apoptotic and senescent cells, and actively shape tissue architecture during organogenesis and regeneration (Kierdorf et al, 2015, Lendeckel et al, 2022; Hatch et al, 2024; Watanabe et al, 2019). Macrophages can be broadly classified according to their developmental origin: (i) monocyte-derived, which differentiate from bone marrow-derived monocytes that circulates in the bloodstream and enter tissue in response of tissue homeostasis or inflammation, and (ii) tissue-resident macrophages, which originates from embryonic erytho-myeloid progenitors in the yolk sac or fetal liver, which seed developing tissues during embryogenesis and can persist into adulthood (Haldar & Murphy, 2014). Tissue-resident macrophages acquire tissue-specific functions and are present in numerous tissues, including e.g., Kupffer cells in liver, microglia in brain, osteoclasts in bone, alveolar macrophages in lungs, Hofbauer cells in placenta, Langerhans cells in skin and many others (Lendeckel et al, 2022; Mass et al, 2023). Importantly, there is increasing evidence that macrophages are also crucial for the maintenance of stem cell niches in various tissues, including intestines, muscles, mammary gland, the hematopoietic niche and others (Manneken & Currie, 2023a; Ratnayake et al, 2021a; Zhou et al, 2023; Kayvanjoo et al, 2024; Winkler et al, 2010). In this study we focus on "tooth macrophages" as a broad term for the macrophage population located in the mice teeth regardless of their primary origin (monocyte-derived or tissue-resident). Despite the advance of*

studying macrophages in different organs, the role of tooth macrophages in the context of the stem cell niche of incisor and molar morphogenesis still remains a completely unexplored area".

We introduced these changes to adequate the terminology use in the main text as follows:

Page 5, lines 12-13 (Results): "*Tooth macrophages populate early embryonic developing teeth and expand postnatally before eruption*."

Page 6, lines 5-7 (Results): "*Co-staining of macrophages with mKI67, a marker of proliferating cells, revealed their proliferative capacity within the teeth, suggesting that these cells are ~~pulp-resident and~~ maintained through local proliferation (Fig. 1*i)"

Page 6, lines 11-12 (Results): "*In tooth, similar to other organs such as mammary gland or salivary gland, ~~resident~~ macrophages are in close contact with heterogenous cell populations as integral part of the microenvironment (Zhou et al,2023; McKendrick et al, 2023).*"

Page 14, lines 6-7 (Discussion): "*Throughout these developmental stages, ~~tissue-resident~~ tooth macrophages emerge as abundant component of the tooth microenvironment*"

Page 17, lines 11-13 (discussion): "*This, coupled with our observation of actively proliferating ~~tissue-resident~~ tooth macrophages in adult teeth, suggests that macrophages quickly repopulate impacted dental tissue.*"

Page 17, lines 19-21 (Discussion): "*In summary, our findings suggest that after injury, the population of ~~tissue-resident~~ tooth macrophages rapidly recovers and activates an M2 healing phenotype which leads to the swift restoration of a healthy tooth morphogenesis*"

Page 18, lines 3-4 (Discussion): "*Therefore, a mechanistically holistic approach - one that jointly examines odontogenic and osteogenic processes and integrates the contributions of ~~resident~~ immune and stromal cells*"

Page 34, lines 6-7 (Figure legend): "*Immunohistochemical co-staining using AIF1 and F4/80 antibodies show co-localization of both markers ~~in dental pulp resident of~~ tooth macrophages.*"

Page 38, line 5 (Figure legend): "*Supplementary Figure 2. Csf1 signaling system is the tooth~~-resident~~ macrophages*"

Moreover, to completely clear that our main aim in the paper was to study tooth macrophages independently from their developmental origin, we included in the introduction this clear statement as follows:

Page 4, lines 10-11: *"This study investigates the role of tooth macrophages independently of their origin (this term does not distinguish between monocyte-derived or tissue resident macrophages) during development, growth and interaction with the stem cell niche during continuous tooth growth."*

6. I was disappointed to see that the authors remained fixed on using histological sections and unreliable quantification in immunostained sections to analyse the effect of mutations or treatments on macrophage population in dental tissues. The credibility and accuracy of their data would be significantly increased if they used flow cytometry, particularly in case of overexposure and high background which exist in their

immunostainings: many of the cells show nuclear staining for what should be surface marker and co-localization studies are not of best quality. Flow cytometry would provide a great depth to their study.

We are not sure what exactly the reviewer means by this question. We believe this is a misunderstanding or the result of an insufficient review of our work.

We have already performed and presented an extensive and statistically significant flow cytometry analysis as part of our revised manuscript. Importantly, in parallel, we performed quantitative immunohistochemical stainings (IHC) which also show robust and statistically highly significant results. Moreover, our IHC provide a crystal clear and specific (not nuclei as the reviewer wrongly claims) signal without background – we used a validated, well-known and highly specific antibody. Then, in addition to quantitative flow cytometry data we present spatial distribution data using IHC, which are quantitative in parallel as well.

- Quantitative flow cytometry (FC) analysis**, shown in Fig 6c,d,** and commented in the main text as follows**:**

Page 11, lines 32-36 Page 12, lines 1-3 (Results): *"In acute response experiment we detected a significant drop of amount of F4/80+ cells from 15.23 ± 0.25 % (Mean ± standard deviation) (untreated condition) to 4.97 ± 1.46 % in the acute condition. The ratio of F4/80+ macrophages was restored after recovery period to 17.5 ± 4.81 % one week after last Clodrosome delivery (Fig. 6c). Additionally, we analyzed the ratio of Gr-1+ cells (common marker of granulocytes, differentiating monocytes and macrophages) after the Clodrosome treatment. FC analysis showed a similar trend to that observed for F4/80 with ratio of Gr-1+ cells of 1,43 ± 0.35% in controls, 0.66% in acute-response and 1.66% in the recovered group (Fig. 6d)."*

- Co-detection by immunohistochemistry (AIF1 and F4/80)**, shown in Fig 6e-g,** and addressed in the text as continues:

Page 12, lines 3-5 (Results): *"Likewise, quantification of AIF1 expression by immunohistochemistry on serial paraffin sections revealed a reduced number of AIF1+ cells in both the dental pulp (Fig. 6f) and the epithelial compartment of LaCL (Fig. 6g)."*

7. Similarly, lack of mechanistic studies undermines their conclusions, and computational modelling of cell-cell and molecular interactions does not provide direct or conclusive evidence.

We thank the reviewer for this comment and agree that the study is primarily focused on the cellular role of macrophages rather than providing a deep molecular dissection.
To touch the mechanism, in response to comments raised during the first revision, we expanded the manuscript to include additional analyses addressing potential mechanisms of **macrophage–cell communication**. Specifically, **we added a new Fig. 2 presenting ligand–receptor interaction analysis** based on previously published single-cell RNA-seq data (*Krivanek et al., 2020*), including CellChat analysis that **identified putative signaling interactions between macrophages and epithelial cell populations, such as the Lgals9–Cd44 axis**. These computational findings were further **validated with histological immunohistochemistry** assays showing macrophages in close proximity to CD44+ epithelial progenitor cells. In addition, **we included new analyses demonstrating epithelial abnormalities and reduced enamel volume in macrophage-deficient mice** (Fig. 3k,l and Supplementary Fig. 4).

We emphasized this aspect in the discussion as follows:

Page 14, lines 32-35; Page 15, lines 1-5 (Discussion): *"The mechanisms by which macrophages can exert their influence may be either in providing support for proliferation (Liu et al, 2016), producing growth factors (such as VEGFs) (Lai et al, 2019), or regulation of inflammation (Watanabe et al). Ligand–receptor analysis identified the macrophage Lgals9 ligand and epithelial CD44 receptor as a putative communication axis, suggesting that macrophage–epithelium crosstalk may mediate the regulation of proliferation and differentiation.* ***However, while this data provides an initial insight into macrophage-mediated signalling within the epithelial niche, the precise molecular mechanism by which macrophages influence surrounding epithelial or mesenchymal stem cell niches to ensure proper tooth development remains to be investigated in future studies****."*

Conclusion of Reviewer: These downsides emphasize the overall descriptive quality of the study and speculative character of the conclusions, which in my opinion is not suitable for publication in Nature Communications.

Again, we regret that the reviewer did not accept our previous improvements and we sincerely hope that new experiments and quantifications will present a more positive case. Importantly, although we did not explore the molecular mechanism, this study cannot be considered "descriptive", as it largely presents the results of genetic and pharmacological experimentation with causative consequences. We hope the reviewer agrees on this point and sees a potential of the revised study.

Reviewer #4 (Remarks to the Author):

Per the editor's request, I am commenting on the authors' response to Reviewer #1. In my view, most of the major concerns raised by Reviewer #1 have been adequately addressed in the revised manuscript.

We want to thank to Reviewer #4 for dedicating their time and effort spent in the revised version of the manuscript. We truly appreciate the positive feedback on our and we believe that we have addressed the remaining issues in the second round of revision.

1. The understanding of the complex biological systems driving tooth formation and eruption has been improved with revised text and additional experiments.

We sincerely appreciate this feedback.

2. I agree with the authors that the main focus of this study is to elucidate the role of macrophages in tooth, leveraging mouse models with disruptions in the Csf1-Csf1r axis and pharmaceutical depletion of macrophage; the exact action mechanisms of individual molecules or cytokines are not the focus of this study.

We sincerely the support and fair opinion of the reviewer.

3. However, the distinction between tissue-resident macrophages and monocyte-derived macrophages

remains unclear in the revised manuscript. In the field, tissue-resident macrophages typically refer to cells originate during embryonic development and primarily maintain organ homeostasis under steady-state conditions. Monocyte-derived macrophages arise from circulating monocytes, are recruited to tissues, and generally function in pathogen defense or tissue repair. Distinguishing whether a macrophage population is tissue-resident or monocyte-derived is indeed challenging. Nevertheless, several established approaches can help address macrophage origin, including lineage tracing, marker expression (may be tissue-dependent), monocyte depletion/transfer, and scRNA-seq identity and trajectory analyses. While the authors have provided additional data, the manuscript does not interpret these data with sufficient clarity regarding macrophage origin. This likely reflects an incomplete understanding of the definitions and experimental approaches used to distinguish tissue-resident from monocyte-derived macrophages. The interpretations of the data regarding this point should be made cautiously and clearly based on the presented results and existing literature, to avoid the confusion to the readers.

We apologize for the excessive terminology we used in the previous version of our manuscript. We have corrected this issue in this revision.

The central point of this study was to clarify the role of macrophages on tooth development – regardless of their primary origin (monocyte-derived or are tissue-resident). The origin of macrophages is not essential for the central message of our manuscript (general role of macrophages in tooth development and homeostasis) and neither of the potential outcomes would change the central message of this study.

Nevertheless, to avoid any misunderstanding or misinterpretation we decided to address this, by completely removing the terms tissue-resident or tooth resident-macrophages in our results ". **Instead, we have introduced the term, "Tooth macrophages," which encompasses both of these groups.**

We explain this term at the very beginning of the manuscript to avoid any confusion:

Page 2, lines 20-35; Page 3, lines 1-6 (Introduction): "*Macrophages regulate complex cellular dynamics of tissues during development, growth, repair, and homeostasis in all tissues, as reviewed by many research teams (Mass et al, 2023; Guan et al, 2025; Zhao et al, 2024; Theret et al, 2019; Sehgal et al, 2021; Wynn et al, 2013). Beyond their critical immune role as first-line defenders against pathogens, they initiate adaptive immune response, clear apoptotic and senescent cells, and actively shape tissue architecture during organogenesis and regeneration (Kierdorf et al, 2015, Lendeckel et al, 2022; Hatch et al, 2024; Watanabe et al, 2019). Macrophages can be broadly classified according to their developmental origin: (i) monocyte-derived, which differentiate from bone marrow-derived monocytes that circulates in the bloodstream and enter tissue in response of tissue homeostasis or inflammation, and (ii) tissue-resident macrophages, which originates from embryonic erytho-myeloid progenitors in the yolk sac or fetal liver, which seed developing tissues during embryogenesis and can persist into adulthood (Haldar & Murphy, 2014). Tissue-resident macrophages acquire tissue-specific functions and are present in numerous tissues, including e.g., Kupffer cells in liver, microglia in brain, osteoclasts in bone, alveolar macrophages in lungs, Hofbauer cells in placenta, Langerhans cells in skin and many others (Lendeckel et al, 2022; Mass et al, 2023). Importantly, there is increasing evidence that macrophages are also crucial for the maintenance of stem cell niches in various tissues, including intestines, muscles, mammary gland, the hematopoietic niche and others (Manneken & Currie, 2023a; Ratnayake et*

al, 2021a; Zhou et al, 2023; Kayvanjoo et al, 2024; Winkler et al, 2010). In this study we focus on "tooth macrophages" as a broad term for the macrophage population located in the mice teeth regardless of their primary origin (monocyte-derived or tissue-resident). Despite the advance of studying macrophages in different organs, the role of tooth macrophages in the context of the stem cell niche of incisor and molar morphogenesis still remains a completely unexplored area".

We introduced these changes to adequate the terminology use in the main text as follows:

Page 5, lines 12-13 (Results): "*Tooth macrophages populate early embryonic developing teeth and expand postnatally before eruption*."

Page 6, lines 5-7 (Results): "*Co-staining of macrophages with mKI67, a marker of proliferating cells, revealed their proliferative capacity within the teeth, suggesting that these cells are ~~pulp-resident and~~ maintained through local proliferation (Fig. 1*i)"

Page 6, lines 11-12 (Results): "*In tooth, similar to other organs such as mammary gland or salivary gland, ~~resident~~ macrophages are in close contact with heterogenous cell populations as integral part of the microenvironment (Zhou et al,2023; McKendrick et al, 2023).*"

Page 14, lines 6-7 (Discussion): "*Throughout these developmental stages, ~~tissue-resident~~ tooth macrophages emerge as abundant component of the tooth microenvironment*"

Page 17, lines 11-13 (discussion): "*This, coupled with our observation of actively proliferating ~~tissue-resident~~ tooth macrophages in adult teeth, suggests that macrophages quickly repopulate impacted dental tissue.*"

Page 17, lines 19-21 (Discussion): "*In summary, our findings suggest that after injury, the population of ~~tissue-resident~~ tooth macrophages rapidly recovers and activates an M2 healing phenotype which leads to the swift restoration of a healthy tooth morphogenesis*"

Page 18, lines 3-4 (Discussion): "*Therefore, a mechanistically holistic approach - one that jointly examines odontogenic and osteogenic processes and integrates the contributions of ~~resident~~ immune and stromal cells*"

Page 34, lines 6-7 (Figure legend): "*Immunohistochemical co-staining using AIF1 and F4/80 antibodies show co-localization of both markers ~~in dental pulp resident~~ of tooth macrophages.*"

Page 38, line 5 (Figure legend): "*Supplementary Figure 2. Csf1 signaling system is the tooth~~-resident~~ macrophages*"

Moreover, to completely clear that our main aim in the paper was to study tooth macrophages independently from their developmental origin, we included in the introduction this clear statement as follows:

Page 4, lines 10-11: *"This study investigates the role of tooth macrophages independently of their origin (this term does not distinguish between monocyte-derived or tissue resident macrophages) during development, growth and interaction with the stem cell niche during continuous tooth growth."*

Despite this, we appreciate that the reviewer brought up this point and we agree that assessing the origin of the macrophages is an interesting future direction of the study. As suggested by the reviewer, lineage tracing would be a reliable methodology how to assess this. However, performing such experiments would require the introduction and breeding of additional genetically modified mouse strains, which would considerably extend the timeline of the study and is therefore beyond the scope of the current revision.

Importantly, although lineage-tracing experiment could provide insight into macrophage origin, the potential outcomes would not alter the central conclusions of our study, which focus on the general role of macrophages in tooth development and tissue homeostasis rather than their precise developmental origin.

4. Minor suggestion: The abstract could be more informative, particularly in the pharmacological depletion section, and would benefit from more clearly specifying the role of macrophages in teeth.

We thank this comment from the reviewer. We adapted the abstract to make more informative the section referring to the clodrosome experiment. Therefore, the new version of the abstract is as follows:

Page 2, lines 2-17: "*The role of macrophages in teeth, beyond their function in innate immunity, remains unexplored. This study demonstrates that macrophages populate dental tissues during early development and increase in number during pre-eruptive stages postnatally. In continuously growing teeth, they are associated with epithelial and mesenchymal stem cell niches throughout lifetime. To investigate their role in development, we genetically disrupted macrophage migration using neural-crest specific Wnt1*^{Cre}*/Csf1*$^{fl/fl}$ *and general Csf1R knockout mouse models. This resulted in abnormal dentin and enamel deposition, eruption defects, early tooth mispatterning, and impaired osteogenesis. Notably, the phenotype was partially rescued via bone marrow transplantation. In addition, the short-term pharmacological depletion of macrophages in wildtype adult mice caused striking disintegration of both dentin and enamel of the apical part of continuously-growing teeth, which was restored after withdrawal of clodronate. Following treatment, macrophages rapidly repopulated dental tissue and the presence of M2 reparative state phenotype was observed by single-cell RNA-seq analysis. Overall, our findings reveal essential role of macrophages in the dental development and patterning of both mesenchymal and epithelial tooth compartments. This previously unrecognized role of macrophages in teeth is reminiscent of their function in complex tissue regeneration, and requires future studies to dissect precise cellular and molecular mechanisms*."
